# Copper(i) reagent-promoted hydroxytrifluoromethylation of enamides: flexible synthesis of substituted-3-hydroxy-2-aryl-3-(2,2,2-trifluoro-1-arylethyl)isoindolin-1-one†

**DOI:** 10.1039/c8ra04088e

**Published:** 2018-07-19

**Authors:** Qing Wang, Peng Shi, Runsheng Zeng

**Affiliations:** Key Laboratory of Organic Synthesis of Jiangsu Province, College of Chemistry Chemical Engineering and Materials Science, Soochow University Suzhou Jiangsu 215123 China zengrunsheng@suda.edu.cn

## Abstract

A novel CuBr-catalyzed hydroxytrifluoromethylation reaction was investigated. Substituted 3-benzylidene-2-arylisoindolin-1-ones was reacted with sodium trifluoromethanesulfinate to afford substituted-3-hydroxy-2-aryl-3-(2,2,2-trifluoro-1-arylethyl)isoindolin-1-one. The reaction proceeded at 25 °C in air atmosphere in the absence of base and ligands. Our results indicate that trifluoromethyl free radical tends to attack a double bond rather than aryl in this reaction.

## Abstract 2

A novel CuBr-catalyzed hydroxytrifluoromethylation reaction was investigated. Substituted 3-benzylidene-2-arylisoindolin-1-ones were reacted with sodium trifluoromethanesulfinate to afford substituted-3-hydroxy-2-aryl-3-(2,2,2-trifluoro-1-arylethyl)isoindolin-1-one.

## Graphical abstract

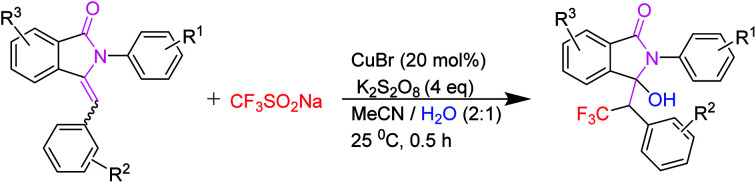

## Introduction

**Fig. 1:**
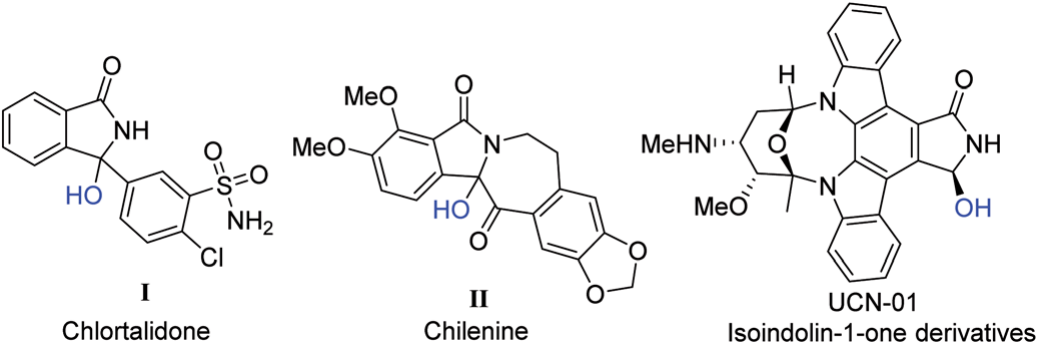
Bioactive and drug value compounds containing 3-hydroxyisoindolin-1-one motifs.

Isoindoles are a series of notable nitrogen-containing compounds known for their bioactivity in nature.^1^ In particular, 3-hydroxyisoindolin-1-ones such as I and II are the core structural motifs of several compounds of medicinal value (Fig. 1). 3-Hydroxy isoindolin-1-ones are known for their use as diuretic and anticancer drugs.^2^ As substituted 3-benzylidene-2-arylisoindolin-1-ones have double bonds, we try to find a catalytic system for direct hydroxytrifluoromethylation of substituted 3-benzylidene-2-arylisoindolin-1-one.

**Scheme 1:**
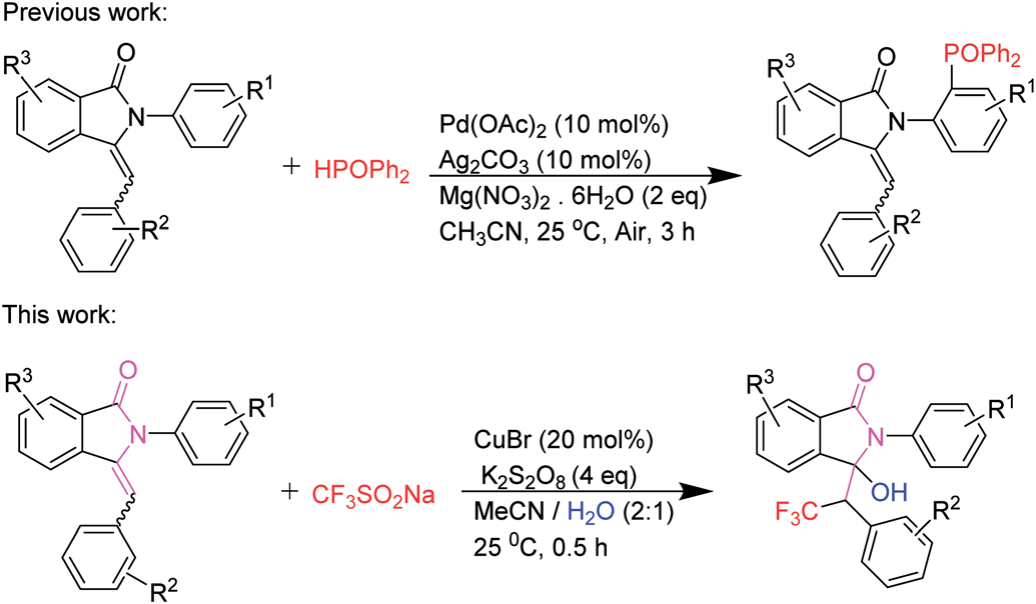


Hydroxytrifluoromethylation of organic molecules has become a research focus in the field of organic synthesis for its unique biological activities.^3^ In 1991, Langlois and co-workers reported the first use of CF_3_SO_2_Na as the trifluoromethyl radical source.^4^ Since then, a series of trifluoromethylation of olefins by using Langlois reagent has been published in the last twenty years.^5^ In these reactions, CF_3_SO_2_Na was excited by single electron oxidations to generate CF_3_ free radicals. The oxidative partners included TBHP,^6^ K_2_S_2_O_8_,^7^ PhI(OAc)_2_,^8^ DTBP,^9^ I_2_O_5_,^10^ metal (Cu, Mn),^11^ photoinducers^12^ and so on. There have only been several examples involving hydroxytrifluoromethylation of olefins to afford useful β-trifluoromethyl alcohols.^13^ Moreover, there were also studies of the metal-free-catalyzed hydroxytrifluoromethylation reactions of styrenes.^14^ Recently, manganese-catalyzed direct hydroxytrifluoromethylation reaction of styrene derivatives has been established.^15^ On the other hand, the CuCF_3_ system has been used for the synthesis of direct hydroxytrifluoromethylation reaction.^16^ Visible light promoted C–F functionalization has been developed under mild reaction condition.^17^ Until now, hydroxytrifluoromethylation of enamides has not been reported. As part of our research on the transition metal-catalyzed free radical reaction of substituted 3-benzylidene-2-arylisoindolin-1-one,^18^ this communication reports the first example of hydroxytrifluoromethylation reaction of 3-benzylidene-2-arylisoindolin-1-one (the special structure of enamide) with sodium trifluoromethanesulfinate catalyzed by CuBr in the presence of K_2_S_2_O_8_ (Scheme 1).

## Results and discussion

When the model reaction of 3-benzylidene-2-phenylisoindolin-1-one (1a) with sodium trifluoromethanesulfinate (2) was performed in CH_3_CN/H_2_O in the presence of oxidants such as TBHP, DTBP, Mn(OAc)_3_ and PhI(OAc)_2_, no desired products were obtained (Table 1, entries 1, 2, 3 and 4). After the addition of K_2_S_2_O_8_ (4 eq.), the reaction proceeded smoothly to afford the desired product, 3-hydroxy-2-phenyl-3-(2,2,2-trifluoro-1-phenylethyl)isoindolin-1-one (3a) in 48% yield (Table 1, entry 5). Further investigation of copper catalysts, the yield of 3a was improved to 72% when we used CuBr as the catalyst (Table 1, entries 9). On the other hand, when we use FeCl_3_ in place of CuBr, the reaction afforded the desired product in a lower yield (Table 1, entry 12). By screening polar mixed solvents such as DMSO/H_2_O DMF/H_2_O, THF/H_2_O, acetone/H_2_O, and a representative nonpolar solvent, toluene (Table 1, entries 15–19), we found that CH_3_CN/H_2_O (2 : 1) works best for the reaction. Apart from the above-mentioned factors, the effects of catalyst loading, reaction temperature and time were also investigated, and the optimal reaction conditions were determined to be room temperature reaction for 0.5 h in air atmosphere, with the addition of 20 mol% CuBr as catalyst, K_2_S_2_O_8_ as single electron oxidation regent and CH_3_CN/H_2_O as solvent (Table 1, entries 20–27).

**Table 1:** Optimization of the reaction conditions^a^

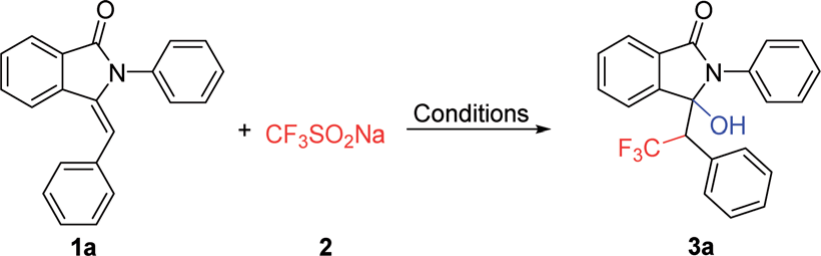					
Entry	Catalyst (%)	Oxidant	Temperature	Solvent (66.7%)	Yield^b^
1		TBHP	25 °C	CH_3_CN	N.D
2		DTBP	25 °C	CH_3_CN	N.D
3		Mn(OAc)_3_	25 °C	CH_3_CN	N.D
4		PhI(OAc)_2_	25 °C	CH_3_CN	N.D
5		K_2_S_2_O_8_	25 °C	CH_3_CN	48%
6	CuO (20)	K_2_S_2_O_8_	25 °C	CH_3_CN	30%
7	Cu(OAc)_2_ (20)	K_2_S_2_O_8_	25 °C	CH_3_CN	55%
8	CuCl (20)	K_2_S_2_O_8_	25 °C	CH_3_CN	60%
9	CuBr (20)	K_2_S_2_O_8_	25 °C	CH_3_CN	72%
10	CuI (20)	K_2_S_2_O_8_	25 °C	CH_3_CN	28%
11	CuBr_2_ (20)	K_2_S_2_O_8_	25 °C	CH_3_CN	50%
12	FeCl_3_ (20)	K_2_S_2_O_8_	25 °C	CH_3_CN	45%
13	Ag_2_CO_3_ (20)	K_2_S_2_O_8_	25 °C	CH_3_CN	48%
14	CuBr (20)	K_2_S_2_O_8_	25 °C	CH_3_CN	48%
15	CuBr (20)	K_2_S_2_O_8_	25 °C	DMSO	6%
16	CuBr (20)	K_2_S_2_O_8_	25 °C	DMF	10%
17	CuBr (20)	K_2_S_2_O_8_	25 °C	THF	37%
18	CuBr (20)	K_2_S_2_O_8_	25 °C	Acetone	55%
19	CuBr (20)	K_2_S_2_O_8_	25 °C	Toluene	0%
20	CuBr (20)	K_2_S_2_O_8_	50 °C	CH_3_CN	65%
21	CuBr (20)	K_2_S_2_O_8_	80 °C	CH_3_CN	60%
22	CuBr (20)	K_2_S_2_O_8_	10 °C	CH_3_CN	66%
23^c^	CuBr (20)	K_2_S_2_O_8_	25 °C	CH_3_CN	35%
23^d^	CuBr (20)	K_2_S_2_O_8_	25 °C	CH_3_CN	28%
23^e^	CuBr (20)	K_2_S_2_O_8_	25 °C	CH_3_CN	70%
24^f^	CuBr (20)	K_2_S_2_O_8_	25 °C	CH_3_CN	46%
25^g^	CuBr (20)	K_2_S_2_O_8_	25 °C	CH_3_CN	70%
26^h^	CuBr (20)	K_2_S_2_O_8_	25 °C	CH_3_CN	70%
27	CuBr (10)	K_2_S_2_O_8_	25 °C	CH_3_CN	60%

a Reaction conditions: 1a (1 mmol), 2, (3 mmol), CuBr, (0.2 mmol), K_2_S_2_O_8_ (4 mmol), solvent (10 ml), at 25 °C in air atmosphere, 30 min.

b Yields are given for isolated products.

c CH_3_CN/H_2_O = 5/1.

d CH_3_CN/H_2_O = 1/1.

e K_2_S_2_O_8_ (5 mmol) was added.

f K_2_S_2_O_8_ (3 mmol) was added.

g 1 h.

h In argon atmosphere.

With the promising results obtained in the model reaction, we subsequently examined the substrate scope of 3-benzylidene-2-arylisoindolin-1-one under the optimized reaction conditions (20 mol% CuBr as catalyst, and K_2_S_2_O_8_ as oxidant in CH_3_CN/H_2_O (2 : 1) at 25 °C, for 0.5 h in air atmosphere).

As shown in Table 2, electron-donating substituents such as methyl and methoxy groups on the aryl ring of substituted 3-benzylidene-2-arylisoindolin-1-one (1) facilitated the reaction to afford the hydroxytrifluoromethylation products (3) in moderate to good yields (Table 2, 65–83%, 3a–3d, 3h–3j, 3o–3p, and 3t). On the contrary, election-withdrawing groups such as F and Cl were unfavorable for the reaction and led to lower yields (Table 2, 34–67%, 3e–3g, 3k–3n and 3r–3s). We also found that when the substrate was 3-pentylidene-2-phenylisoindolin-1-one, the target product (3u) was in 85% yield but diastereomeric ratio is 1 : 1.

**Table 2:** Scope studies of 3-hydroxy-2-phenyl-3-(2,2,2-trifluoro-1-phenylethyl)isoindolin-1-one^a^

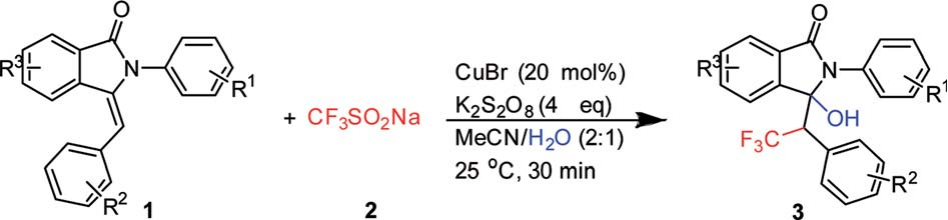
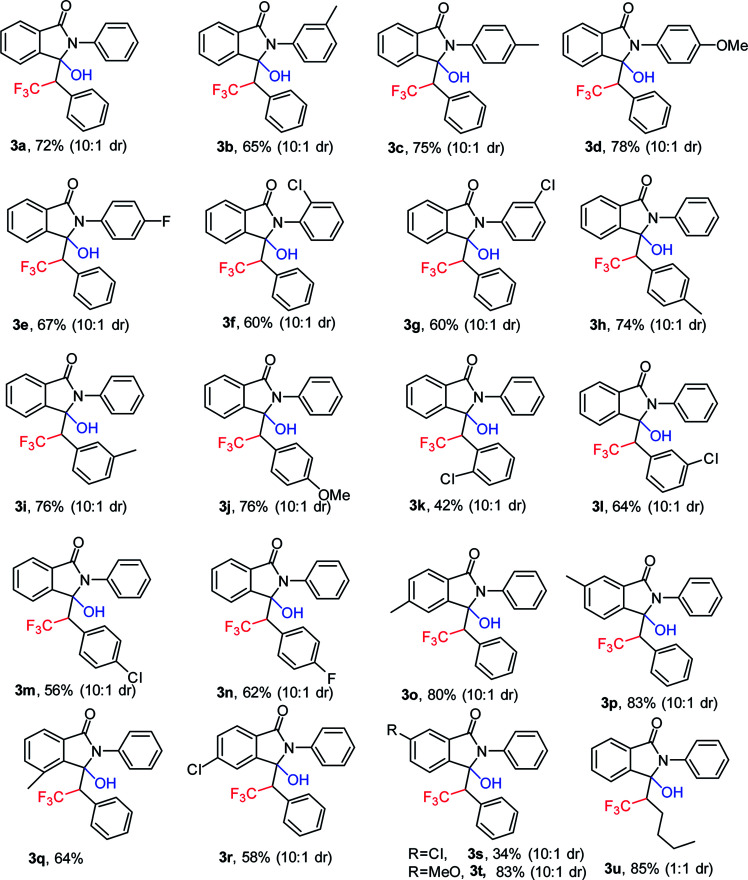

a Reaction conditions: 1 (1 mmol), 2 (3 mmol), CuBr, (0.2 mmol), K_2_S_2_O_8_ (4 mmol), CH_3_CN/H_2_O 2 : 1 (10 ml), at 25 °C in air atmosphere, 30 min. Yield of isolated products are given.

**Scheme 2:**
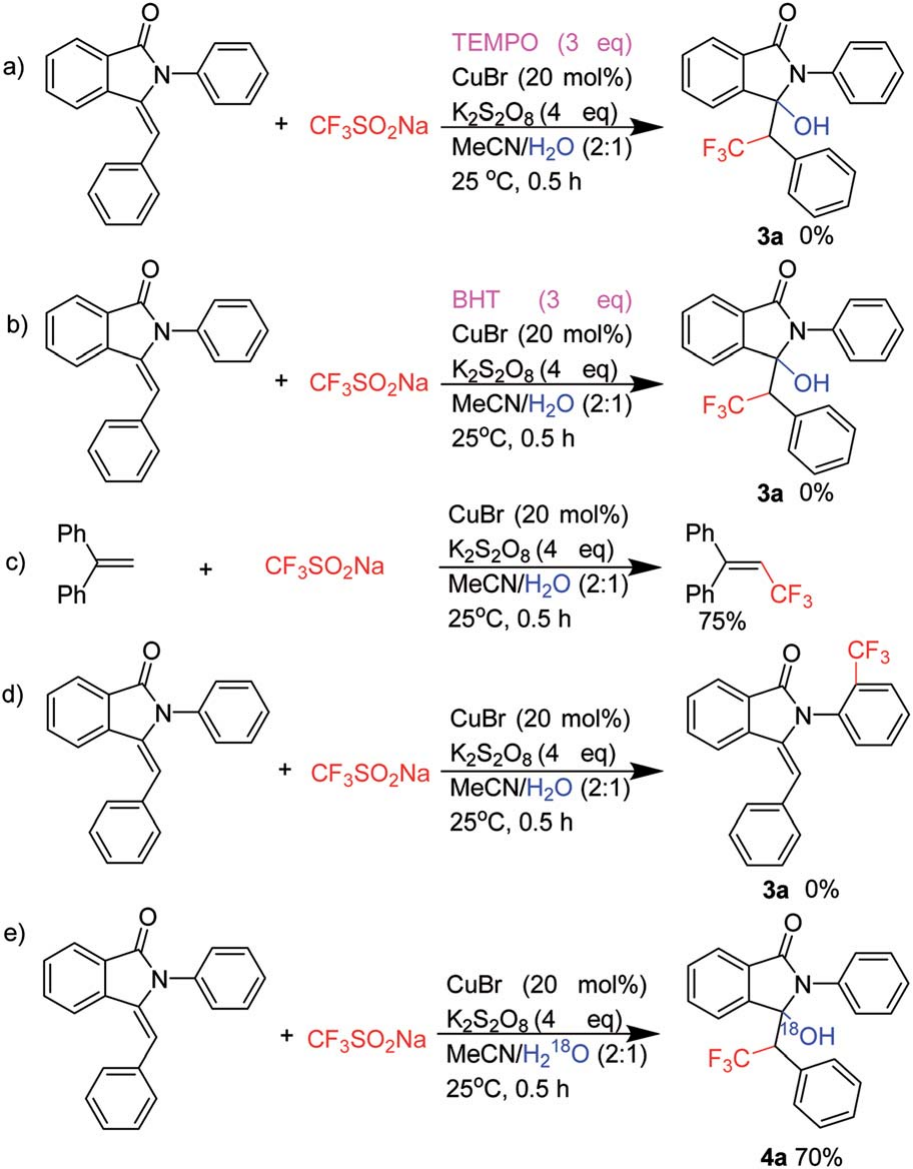
Control experiments.

In order to understand the reaction mechanism, following control experiments were carried out. We repeated the reaction in the presence of radical quencher 2,2,6,6-tetramethylpiperidin-1-oxyl (TEMPO) and none of 3a was obtained (Scheme 2a). The result suggested that free radical were probably generated during the reaction. Furthermore, 3a was also not detected when the reaction was performed with the addition of butylated hydroxytoluene (BHT, 3.0 equiv.) under the standard conditions (Scheme 2b). Trifluoromethylation products was obtained when 1,1-diyldibenzene and Langlois reagent were carried out in standard condition (Scheme 2c). On the other hand, neither aryl amine nor benzylamine substrate produced the *ortho*-position C–H activated products (Scheme 2d).^18^ These results indicated that the reaction is only suitable for enamine substrates which have enough electron cloud density.

As the hydroxytrifluoromethylation always took place under argon atmosphere in the above experiments, we wondered whether the reaction would proceed if isotopically labelled molecular H_2_^18^O was used. Hence, we did further reactions (Scheme 2e). Surprisingly, the corresponding ^18^O-containing product 4a was obtained in 70%. These results further indicated that the oxygen source of this reaction is derived from H_2_O rather than oxygen gas.

**Scheme 3:**
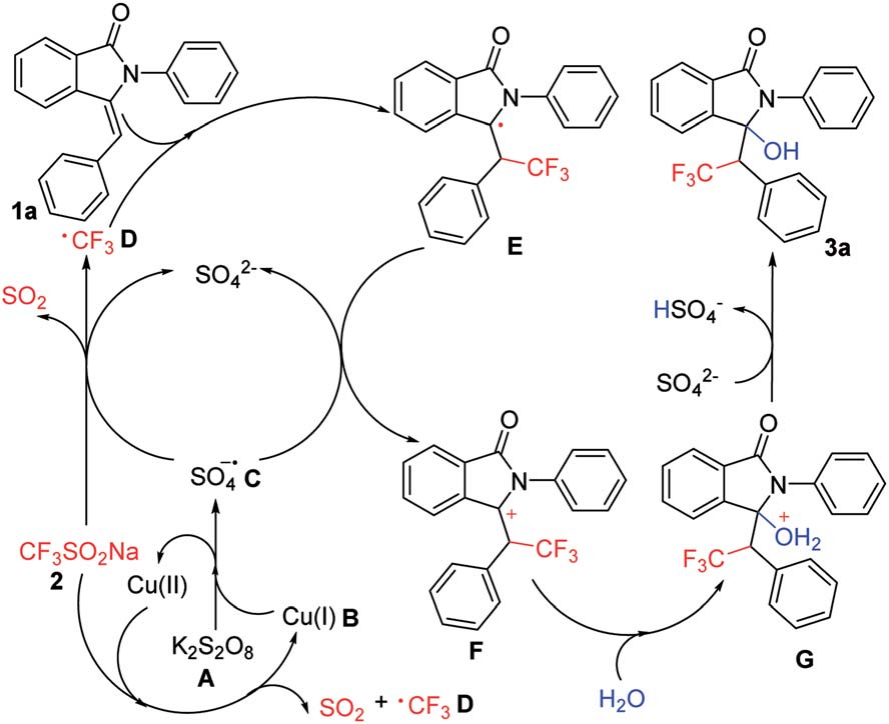
Proposed reaction mechanism.

On the basis of the mechanistic studies and experimental results, a plausible mechanism is proposed in Scheme 3.

Initially, the K_2_S_2_O_8_ (A) was excited by Cu(i) (B) to generate the intermediate SO_4_ radical anions (C), which then reacted with CF_3_SO_2_Na (2) to form trifluoromethyl free radical (D). D underwent addition with substrate (1a) to form key radical intermediate E. Thereafter, the radical intermediate E was oxidized by SO_4_ radical anions (C) which can regenerate SO_4_^2−^ to produce the cation intermediate F. Due to the presence of H_2_O, the cation intermediate F underwent nucleophilic addition to generated the corresponding intermediate G. The cation intermediate G underwent removing protons to generated the corresponding product 3a and HSO_4_^−^. Finally, Cu(ii) was reduced to Cu(i) by CF_3_SO_2_Na (2) to complete the catalytic cycle.

## Conclusions

In summary, we have developed a novel catalytic system for direct hydroxytrifluoromethylation of substituted 3-benzylidene-2-arylisoindolin-1-ones *via* a radical pathway. The reaction has a high regioselectivity as the CF_3_ free radical is prone to attacking a double bond rather than the aryl. The method has a broad scope and offers a good yield. The corresponding products are potentially useful in drug discovery.

## Conflicts of interest

There are no conflicts to declare.

## Supplementary Material

RA-008-C8RA04088E-s001
