# Supplementary material for: Copper(i) reagent-promoted hydroxytrifluoromethylation of enamides: flexible synthesis of substituted-3-hydroxy-2-aryl-3-(2,2,2-trifluoro-1-arylethyl)isoindolin-1-one
Source: RSC Adv. 2018 Jul 19;8(46):25961–5. doi: 10.1039/c8ra04088e (PMC9082868; doi:10.1039/c8ra04088e)
Supplement: RA-008-C8RA04088E-s001 [file RA-008-C8RA04088E-s001.pdf]

Electronic Supplementary Material (ESI) for RSC Advances.
This journal is © The Royal Society of Chemistry 2018

Copper (I) Reagent-Promoted Hydroxytrifluoromethylation of Enamides: Flexible Synthesis of Substituted-3-hydroxy-2-aryl-3-(2,2,2-trifluoro-1-arylethyl)isoindolin-1-one

Qing Wang,[a] Peng Shi,[a] and Runsheng Zeng*[a]

[a] Key Laboratory of Organic Synthesis of Jiangsu Province, College of Chemistry Chemical Engineering and Materials Science, Soochow University, Suzhou, Jiangsu 215123, China.

Supporting Information

Table of concents

1. General information. S2
2. Instrumentation. S2
3. Experimental Procedures S2
4. Characterization data S3
5. References:.......... S10
6. NMR spectra S11

1. General information.

Unless otherwise noted, all reactions were carried out open to air in the oven-dried glass tubes with magnetic stirring. All reagents and solvents were purchased for commercial suppliers. Analytical thin layer chromatography (TLC) was performed using Silica Gel 60 F254 aluminum plates and visualized with UV light (254nm). The pure products were obtained by means of column chromatography which was performed on silica gel (200-300 mesh).

2. Instrumentation.

The ^{1}H NMR (400 MHz), ^{13}C NMR (101 MHz), ^{19}F NMR (376MHz) spectra were recorded at 23℃ with DMSO-d6/$CDCl_3$ as solvents on a Bruker 400 spectrometer and tetramethylsilane (TMS) as internal standard. Chemical shifts were reported in ppm from internal TMS (δ), all coupling constants (J values) were reported in Hertz (Hz). High resolution mass spectra (HRMS) were obtained on a TOF machine (ESI-TOF). Multiplicities are recorded as s = singlet, d = doublet, t = triplet, dd = doublet of doublets, br s = broad singlet, m = multiplet.

3. Experimental Procedures

3.1 Preparation of 3-methyleneisoindolin-1-one (1a). [1]

A oven-dried Schlenk tube charged with a magnetic stirring bar was added 2-bromobenzylamide (1.380 g, 5 mmol), CuI (95 mg, 0.5 mmol), L-proline (180 mg, 1.5 mmol) and potassium carbonate (1.380 g, 1 0 mmol), and the tube was evacuated and backfilled with argon (3 times), and then phenylacetylene (830 µl, 7.5 mmol), and i-PrOH (10 mL) were added. The reaction mixture was stirred at 80 ^{0}C for 16h. After removal of i-PrOH, the residues were filtrated with 20 ml water (3 times). The products were purified by regular column chromatography.

This template for synthesis of other substituted 3-methyleneisoindolin-1-one.

3.2 Preparation of 3-hydroxy-2-phenyl-3-(2,2,2-trifluoro-1-phenylethyl)isoindolin-1-one (3a).

To a reaction tube equipped with a magnetic stirring bar were added 3-benzylidene-2-phenylisoindolin-1-one (**1a**) (297 mg, 1 mmol), CF_3SO_2Na (**2**) (474 mg, 3 mmol), CuBr (28.7 mg, 0.2 mmol) and $K_2S_2O_8$ (1082 mg, 4 mmol), and then CH_3CN (6 mL) and H_2O (3 mL) were added. The reaction mixture was stirred at room temperature for 30 min before being quenched by water (20 mL). Extract with ethyl acetate for three times (60 mL) and the organic phase was concentrated in vacuo and purified by column chromatography on silica gel (petroleum ether: ethyl acetate = 6:1) to give the corresponding product **3a**.

This template for synthesis of other substituted **3**-hydroxy-2-phenyl-3-(2,2,2-trifluoro-1-phenylethyl)isoindolin-1-one.

4. Characterization data

3-hydroxy-2-phenyl-3-(2,2,2-trifluoro-1-phenylethyl)isoindolin-1-one (3a).

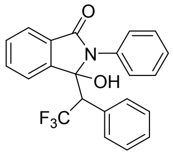

Diastereomeric ratio (major: minor = 10:1) was determined by ^{19}F NMR analysis of the unpurified product mixture. Faint yellow solid, 276 mg (72%); ^{1}H NMR (400 MHz, DMSO-d6) δ 7.98 (s, 1H), 7.91 (d, J = 7.7 Hz, 1H), 7.87 – 7.81 (m, 1H), 7.68 (dd, J = 8.8, 6.9 Hz, 2H), 7.40 (t, J = 7.0 Hz, 4H), 7.32 (d, J = 3.3 Hz, 1H), 7.21 (t, J = 7.4 Hz, 1H), 7.05 (t, J = 7.7 Hz, 2H), 6.41 (d, J = 7.5 Hz, 2H), 4.12 (q, J = 10.5 Hz, 1H); ^{19}F NMR (376 MHz, DMSO-d6) δ -59.31 (major), -59.89 (minor); ^{13}C NMR (101 MHz, DMSO-d6) δ165.39, 144.58, 135.78, 133.29, 132.04, 130.81, 130.48, 130.07, 129.06, 128.85, 128.44, 128.23, 127.05, 126.65, 124.90 (q, J = 6.6 Hz), 123.35, 92.96, 55.95 (q, J = 25.5 Hz); HRMS (ESI-TOF): $[M+Na]^+$ m/z calcd for $C_{22}H_{16}F_3NO_2Na^+$: 406.1031, found: 406.1029.

3-hydroxy-2-(m-tolyl)-3-(2,2,2-trifluoro-1-phenylethyl)isoindolin-1-one (3b)

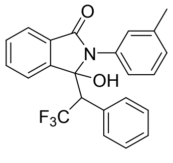

Diastereomeric ratio (major: minor = 10:1) was determined by ^{19}F NMR analysis of the unpurified product mixture. Faint yellow solid, 258 mg (65%); ^{1}H NMR (400 MHz, DMSO-d6) δ 7.94 (s, 1H), 7.84 (t, *J* = 7.2 Hz, 1H), 7.69 (dd, *J* = 13.5, 7.1 Hz, 2H), 7.35 – 7.28 (m, 2H), 7.24 (dd, *J* = 15.4, 7.9 Hz, 2H), 7.15 (d, *J* = 7.2 Hz, 1H), 7.10 – 7.02 (m, 3H), 6.42 (d, *J* = 7.4 Hz, 2H), 4.11 (q, *J* = 10.4 Hz, 1H), 2.29 (s, 3H); ^{19}F NMR (376 MHz, DMSO-d6) δ -59.43 (major), -60.01 (minor); ^{13}C NMR (101 MHz, DMSO-d6) δ164.95, 144.11, 137.43, 135.13, 132.74, 131.70, 130.50, 130.30, 130.06, 129.71, 128.57, 128.14, 127.66, 127.27, 127.21, 124.49 (q, *J* = 6.1 Hz), 123.29, 122.84, 92.34, 55.59 (q, *J* = 25.7 Hz), 21.12; HRMS (ESI-TOF): $[M+Na]^+$ m/z calcd for $C_{23}H_{18}F_3NO_2Na^+$: 420.1187, found: 420.1184.

3-hydroxy-2-(p-tolyl)-3-(2,2,2-trifluoro-1-phenylethyl)isoindolin-1-one (3c)

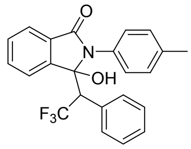

Diastereomeric ratio (major: minor = 10:1) was determined by ^{19}F NMR analysis of the unpurified product mixture. Faint yellow solid, 298 mg (75%); ^{1}H NMR (400 MHz, DMSO-d6) δ 7.92 (s, 1H), 7.85 – 7.80 (m, 1H), 7.66 (t, *J* = 6.5 Hz, 2H), 7.33 (d, *J* = 4.8 Hz, 1H), 7.29 (d, *J* = 8.2 Hz, 2H), 7.22 (dd, *J* = 12.4, 7.8 Hz, 3H), 7.06 (t, *J* = 7.5 Hz, 2H), 6.45 (d, *J* = 7.4 Hz, 2H), 4.09 (q, *J* = 10.5 Hz, 1H), 2.35 (s, 3H); ^{19}F NMR (376 MHz, DMSO-d6) δ -59.24 (major), -59.98 (minor); ^{13}C NMR (101 MHz, DMSO-d6) δ164.87, 144.15, 135.93, 132.67, 132.55, 131.71, 130.26, 130.06, 129.66, 129.12, 128.87, 128.56, 127.73, 126.27, 124.43 (q, *J* = 6.9 Hz), 122.79, 92.40, 55.41 (q, *J* = 25.7 Hz), 20.68 (s); HRMS (ESI-TOF): $[M+Na]^+$ m/z calcd for $C_{23}H_{18}F_3NO_2Na^+$: 420.1187, found: 420.1193.

3-hydroxy-2-(4-methoxyphenyl)-3-(2,2,2-trifluoro-1-phenylethyl)isoindolin-1-one (3d)

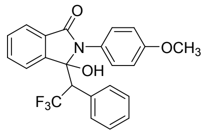

Diastereomeric ratio (major: minor = 10:1) was determined by ^{19}F NMR analysis of the unpurified product mixture. Faint yellow solid, 322 mg (78%); ^{1}H NMR (400 MHz, DMSO-d6) δ 7.89 (d, *J* = 7.4 Hz, 1H), 7.85 (s, 1H), 7.69 – 7.63 (m, 2H), 7.24 (dd, 4H), 7.07 (t, *J* = 7.7 Hz, 2H), 6.98 (d, *J* = 9.0 Hz, 2H), 6.46 (d, *J* = 7.5 Hz, 2H), 4.05 (q, *J* = 10.5 Hz, 1H), 3.80 (s, 3H); ^{19}F NMR (376 MHz, DMSO-d6) δ -59.29 (major), -59.98 (minor); ^{13}C NMR (101 MHz, DMSO-d6) δ164.90, 157.74, 144.15, 132.60, 131.78, 130.48, 130.25, 130.11, 129.73, 128.57, 128.05, 127.75, 127.56, 124.41 (q, *J* = 6.5 Hz), 122.75, 113.58, 92.16, 55.44 (q, *J* = 25.1 Hz), 55.23; HRMS (ESI-TOF): $[M+Na]^+$ m/z calcd for $C_{23}H_{18}F_3NO_3Na^+$: 436.1136, found: 436.1143.

2-(4-fluorophenyl)-3-hydroxy-3-(2,2,2-trifluoro-1-phenylethyl)isoindolin-1-one (3e)

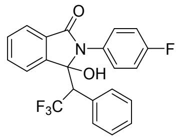

Diastereomeric ratio (major: minor = 10:1) was determined by ^{19}F NMR analysis of the unpurified product mixture. Faint yellow solid, 269 mg (67%);^{1}H NMR (400 MHz, DMSO-d6) δ 8.00 (s, 1H),

7.93 (d, J = 7.5 Hz, 1H), 7.87 – 7.83 (m, 1H), 7.72 (d, J = 6.8 Hz, 1H), 7.68 (d, J = 7.2 Hz, 1H), 7.37 (dd, J = 8.6, 5.1 Hz, 2H), 7.29 (d, J = 9.8 Hz, 1H), 7.26 – 7.22 (m, 2H), 7.07 (t, J = 7.6 Hz, 2H), 6.47 (d, J = 7.4 Hz, 2H), 4.15 (q, J = 10.4 Hz, 1H); ^{19}F NMR (376 MHz, DMSO-d6) δ -59.46 (major), -59.73 (minor), -114.98 (major), -115.21 (minor).; ^{13}C NMR (101 MHz, DMSO-d6) δ164.95, 160.31 (d, J = 244.0 Hz), 144.10, 132.86, 131.48, 131.40 (d, J = 2.7 Hz), 130.36, 130.05, 129.64, 128.62, 128.37 (d, J = 8.4 Hz), 127.91, 127.79, 124.52 (q, J =6.1 Hz), 122.91, 115.18 (d, J = 22.3 Hz), 92.31, 55.49 (q, J =27.3 Hz); HRMS (ESI-TOF): [M+Na]$^+$ m/z calcd for $C_{22}H_{15}F_4NO_2Na^+$: 424.0937, found: 424.0935.

2-(2-chlorophenyl)-3-hydroxy-3-(2,2,2-trifluoro-1-phenylethyl)isoindolin-1-one(3f)

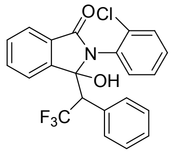

Diastereomeric ratio (major: minor = 10:1) was determined by ^{19}F NMR analysis of the unpurified product mixture. Faint yellow solid, 250 mg (60%); ^{1}H NMR (400 MHz, $CDCl_3$) δ 8.50 (d, J = 6.6 Hz, 1H), 8.05 (s, 1H), 7.60 (d, J = 7.3 Hz, 1H), 7.52 (t, J = 6.8 Hz, 1H), 7.46 – 7.41 (m, 2H), 7.41 – 7.37 (m, 4H), 7.33 (dd, J = 5.0, 1.7 Hz, 3H), 7.12 (td, J = 7.9, 1.4 Hz, 1H), 5.14 (q, J = 8.4 Hz, 1H); ^{19}F NMR (376 MHz, $CDCl_3$) δ -60.56 (minor), -65.94 (major); ^{13}C NMR (101 MHz, $CDCl_3$) δ194.22, 165.98, 137.76, 135.62, 134.38, 131.81, 130.89, 130.24, 129.22, 129.13, 129.09, 127.96, 127.29, 125.49, 125.31, 124.21, 123.24, 122.70, 122.00, 59.33 (q, J = 26.3 Hz); HRMS (ESI-TOF): [M+Na]$^+$ m/z calcd for $C_{22}H_{15}ClF_3NO_2Na^+$: 440.0641, found: 440.0631.

2-(3-chlorophenyl)-3-hydroxy-3-(2,2,2-trifluoro-1-phenylethyl)isoindolin-1-one (3g)

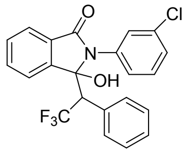

Diastereomeric ratio (major: minor = 10:1) was determined by ^{19}F NMR analysis of the unpurified product mixture. Faint yellow solid, 250 mg (60%); ^{1}H NMR (400 MHz, DMSO) δ 8.12 (s, 1H), 7.94 (d, J = 7.8 Hz, 1H), 7.89 – 7.85 (m, 1H), 7.75 (d, J = 7.2 Hz, 1H), 7.70 (d, J = 7.1 Hz, 1H), 7.48 – 7.44 (m, 2H), 7.40 (dd, J = 5.0, 2.3 Hz, 1H), 7.31 (s, 2H), 7.24 (t, J = 7.4 Hz, 1H), 7.07 (t, J = 7.7 Hz, 2H), 6.44 (d, J = 7.5 Hz, 2H), 4.26 (q, J = 10.3 Hz, 1H); ^{19}F NMR (376 MHz, DMSO) δ -59.55 (major), -59.62 (minor).; ^{13}C NMR (101 MHz, DMSO) δ165.40, 144.44, 137.25, 133.58, 133.05, 131.68, 130.96, 130.73, 130.44, 130.02, 129.47, 129.14, 128.23, 126.82, 126.21, 125.05, 124.68, 123.51, 93.06, 56.01 (q, J = 25.2 Hz); HRMS (ESI-TOF): [M+H]$^+$ m/z calcd for $C_{22}H_{16}ClF_3NO_2^+$: 418.0822, found: 418.0819.

3-hydroxy-2-phenyl-3-(2,2,2-trifluoro-1-(p-tolyl)ethyl)isoindolin-1-one (3h)

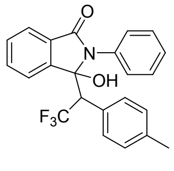

Diastereomeric ratio (major: minor = 10:1) was determined by ^{19}F NMR analysis of the unpurified product mixture. Faint yellow solid, 294 mg (74%); ^{1}H NMR (400 MHz, DMSO-d6) δ 7.95 (s, 1H),

7.90 (d, J = 7.5 Hz, 1H), 7.84 (d, J = 6.5 Hz, 1H), 7.67 (dd, J = 8.5, 6.8 Hz, 2H), 7.46 – 7.39 (m, 4H), 7.35 – 7.32 (m, 1H), 6.85 (d, J = 7.8 Hz, 2H), 6.29 (d, J = 7.6 Hz, 2H), 4.04 (q, J = 10.6 Hz, 1H), 2.17 (s, 3H); ^{19}F NMR (376 MHz, DMSO-d6) δ -59.34 (major), -59.99 (minor); ^{13}C NMR (101 MHz, DMSO-d6) δ165.41, 144.64, 138.45, 135.77, 133.24, 132.09, 130.76, 129.97, 129.04, 128.85, 128.80, 127.42, 127.08, 126.76, 124.91 (d, J = 6.1 Hz), 123.34, 93.04, 55.56 (q, J = 25.4 Hz), 20.98; HRMS (ESI-TOF): $[M+H]^+$ m/z calcd for $C_{23}H_{19}F_3NO_2^+$: 398.1368, found: 398.1327.

3-hydroxy-2-phenyl-3-(2,2,2-trifluoro-1-(m-tolyl)ethyl)isoindolin-1-one (3i)

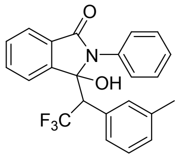

Diastereomeric ratio (major: minor = 10:1) was determined by ^{19}F NMR analysis of the unpurified product mixture. Faint yellow solid, 302 mg (76%); ^{1}H NMR (400 MHz, DMSO-d6) δ 8.02 (s, 1H), 7.95 (d, J = 7.5 Hz, 1H), 7.84 (t, J = 7.2 Hz, 1H), 7.72 (d, J = 7.2 Hz, 1H), 7.68 (d, J = 7.3 Hz, 1H), 7.48 – 7.41 (m, 4H), 7.36 – 7.32 (m, 1H), 7.04 (d, J = 7.5 Hz, 1H), 6.93 (t, J = 7.6 Hz, 1H), 6.29 – 6.12 (m, 2H), 4.10 (q, J = 10.5 Hz, 1H), 2.03 (s, 3H); ^{19}F NMR (376 MHz, DMSO-d6) δ -59.31 (major), -59.88 (minor).; ^{13}C NMR (101 MHz, DMSO-d6) δ164.94, 144.16, 136.79, 135.46, 132.70, 131.70, 130.24, 129.78, 129.08, 128.82, 128.56, 128.24, 127.55, 126.72, 126.50, 126.17, 124.48 (q, J = 6.5 Hz), 122.78, 92.57, 55.57 (q, J = 25.5 Hz), 20.66; HRMS (ESI-TOF): $[M+Na]^+$ m/z calcd for $C_{23}H_{18}F_3NO_2Na^+$: 420.1187, found: 420.1196.

3-hydroxy-2-phenyl-3-(2,2,2-trifluoro-1-(4-methoxyphenyl)ethyl)isoindolin-1-one (3j)

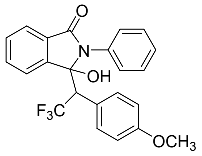

Diastereomeric ratio (major: minor = 10:1) was determined by ^{19}F NMR analysis of the unpurified product mixture. Faint yellow solid, 314 mg (76%); ^{1}H NMR (400 MHz, DMSO-d6) δ 7.93 (s, 1H), 7.89 (d, J = 7.7 Hz, 1H), 7.86 – 7.80 (m, 1H), 7.70 – 7.65 (m, 2H), 7.44 – 7.39 (m, 4H), 7.35 – 7.31 (m, 1H), 6.61 (d, J = 8.9 Hz, 2H), 6.31 (d, J = 8.2 Hz, 2H), 4.02 (q, J = 10.6 Hz, 1H), 3.64 (s, 3H); ^{19}F NMR (376 MHz, DMSO-d6) δ -59.53 (major), -60.12 (minor); ^{13}C NMR (101 MHz, DMSO-d6) δ164.95, 159.17, 144.15, 135.30, 132.76, 131.62, 130.82, 130.30, 128.38, 126.61, 126.30, 124.45 (q, J = 6.4 Hz), 122.88, 121.79, 119.76, 113.13, 92.59, 54.95, 54.67 (q, J = 25.4 Hz); HRMS (ESI-TOF): $[M+Na]^+$ m/z calcd for $C_{23}H_{18}F_3NO_3Na^+$: 436.1136, found: 436.1136.

3-(1-(2-chlorophenyl)-2,2,2-trifluoroethyl)-3-hydroxy-2-phenylisoindolin-1-one (3k)

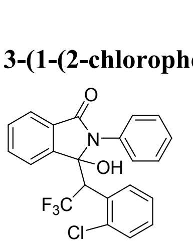

Diastereomeric ratio (major: minor = 10:1) was determined by ^{19}F NMR analysis of the unpurified product mixture. Faint yellow solid, 175 mg (42%); ^{1}H NMR (400 MHz, DMSO-d6) δ 8.06 (s, 1H), 7.89 (d, J = 3.8 Hz, 2H), 7.84 (d, J = 7.4 Hz, 1H), 7.76 (dd, J = 7.8, 3.9 Hz, 2H), 7.51 (dd, J = 20.8,

13.0 Hz, 1H), 7.31 (d, J = 7.5 Hz, 4H), 7.07 (d, J = 7.4 Hz, 2H), 6.22 (d, J = 7.8 Hz, 1H), 4.78 (q, J = 9.7 Hz, 1H); ^{19}F NMR (376 MHz, DMSO-d6) δ -60.81 (major), -61.20 (minor); ^{13}C NMR (101 MHz, DMSO-d6) δ164.84, 143.67, 136.56, 134.81, 133.16, 132.70, 131.72, 130.68, 130.51, 129.44, 128.66, 128.37, 127.68, 126.81, 126.13, 124.62 (q, J = 6.9 Hz), 123.15, 120.03, 92.04, 51.20 (q, J = 26.3 Hz); HRMS (ESI-TOF): $[M+Na]^+$ m/z calcd for $C_{22}H_{15}ClF_3NO_2Na^+$: 440.0641, found: 440.0645.

3-(1-(3-chlorophenyl)-2,2,2-trifluoroethyl)-3-hydroxy-2-phenylisoindolin-1-one (3l)

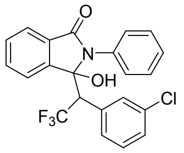

Diastereomeric ratio (major: minor = 10:1) was determined by ^{19}F NMR analysis of the unpurified product mixture. Faint yellow solid, 267 mg (64%); ^{1}H NMR (400 MHz, DMSO-d6) δ 8.07 (s, 1H), 7.93 (d, J = 7.6 Hz, 1H), 7.89 – 7.85 (m, 1H), 7.78 – 7.66 (m, 3H), 7.44 – 7.38 (m, 4H), 7.31 (d, J = 7.6 Hz, 1H), 7.10 (t, J = 7.9 Hz, 1H), 6.42 (d, J = 10.2 Hz, 2H), 4.28 (q, J = 10.3 Hz, 1H); ^{19}F NMR (376 MHz, DMSO-d6) δ -59.46 (major), -59.63 (minor); ^{13}C NMR (101 MHz, DMSO-d6) δ164.92, 143.93, 135.28, 132.94, 132.34, 132.21, 131.42, 130.72, 130.49, 129.91, 129.52, 128.56, 128.39, 126.81, 126.65, 126.08, 124.44 (q, J = 6.9 Hz), 122.94, 92.22, 54.98 (q, J = 25.2 Hz); HRMS (ESI-TOF): $[M+H]^+$ m/z calcd for $C_{22}H_{16}ClF_3NO_2^+$: 418.0822, found: 418.0823.

3-(1-(4-chlorophenyl)-2,2,2-trifluoroethyl)-3-hydroxy-2-phenylisoindolin-1-one (3m)

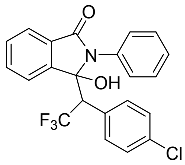

Diastereomeric ratio (major: minor = 10:1) was determined by ^{19}F NMR analysis of the unpurified product mixture. Faint yellow solid, 234 mg (56%); ^{1}H NMR (400 MHz, DMSO-d6) δ 8.04 (s, 1H), 7.91 (d, J = 7.6 Hz, 1H), 7.84 (dd, J = 11.5, 4.7 Hz, 1H), 7.72 (d, J = 6.9 Hz, 1H), 7.68 (d, J = 7.2 Hz, 1H), 7.43 (d, J = 4.2 Hz, 4H), 7.35 – 7.31 (m, 1H), 7.15 (d, J = 8.5 Hz, 2H), 6.46 (d, J = 8.2 Hz, 2H), 4.23 (q, J = 10.4 Hz, 1H); ^{19}F NMR (376 MHz, DMSO-d6) δ -59.45 (major), -59.98 (minor); ^{13}C NMR (101 MHz, DMSO-d6) δ164.91, 143.93, 135.24, 133.46, 132.94, 132.32, 131.42, 130.46, 129.03, 128.48, 127.99, 127.83, 126.64, 126.15, 124.44 (q, J = 6.3 Hz), 122.98, 92.30, 54.72 (q, J = 25.6 Hz); HRMS (ESI-TOF): $[M+Na]^+$ m/z calcd for $C_{22}H_{15}ClF_3NO_2Na^+$: 440.0641, found: 440.0629.

3-hydroxy-2-phenyl-3-(2,2,2-trifluoro-1-(4-fluorophenyl)ethyl)isoindolin-1-one (3n)

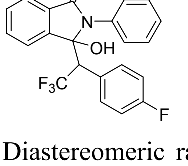

Diastereomeric ratio (major: minor = 10:1) was determined by ^{19}F NMR analysis of the unpurified product mixture. Faint yellow solid, 269 mg (67%); ^{1}H NMR (400 MHz, DMSO-d6) δ 8.04 (s, 1H), 7.94 (d, J = 7.5 Hz, 1H), 7.85 (d, J = 7.3 Hz, 1H), 7.83 – 7.80 (m, 1H), 7.74 (d, J = 7.2 Hz, 1H), 7.68 (d, J = 7.4 Hz, 1H), 7.44 (d, J = 6.8 Hz, 3H), 7.36 – 7.32 (m, 1H), 6.91 (t, J = 8.6 Hz, 2H), 6.53 – 6.44 (m,

2H), 4.23 (q, J = 10.3 Hz, 1H); ^{19}F NMR (376 MHz, DMSO-d6) δ -59.60 (major), -60.12 (minor), -112.97 (major), -113.91 (minor); ^{13}C NMR (101 MHz, DMSO-d6) δ164.94, 161.98 (d, J = 246.1 Hz), 143.98, 135.32, 132.87, 132.54, 131.68, 130.96 (d, J = 112.3 Hz), 128.43, 126.78, 126.59, 126.35, 126.13, 124.46 (q, J = 6.1 Hz), 122.95, 114.70 (d, J = 21.5 Hz), 92.43, 54.65 (q, J = 25.4 Hz); HRMS (ESI-TOF): $[M+Na]^+$ m/z calcd for $C_{22}H_{15}F_4NO_2Na^+$: 424.0937, found: 424.0935.

3-hydroxy-5-methyl-2-phenyl-3-(2,2,2-trifluoro-1-phenylethyl)isoindolin-1-one (3o)

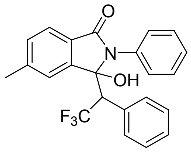

Diastereomeric ratio (major: minor = 10:1) was determined by ^{19}F NMR analysis of the unpurified product mixture. Faint yellow solid, 317 mg (80%); ^{1}H NMR (400 MHz, DMSO-d6) δ 7.92 (s, 1H), 7.70 (s, 1H), 7.58 (d, J = 7.7 Hz, 1H), 7.47 (d, J = 7.7 Hz, 1H), 7.38 (dd, J = 9.8, 7.2 Hz, 4H), 7.34 – 7.30 (m, 1H), 7.21 (t, J = 7.4 Hz, 1H), 7.05 (t, J = 7.7 Hz, 2H), 6.42 (d, J = 7.6 Hz, 2H), 4.10 (q, J = 10.5 Hz, 1H), 2.54 (s, 3H); ^{19}F NMR (376 MHz, DMSO-d6) δ -59.30 (major), -59.83 (minor); ^{13}C NMR (101 MHz, DMSO-d6) δ165.39, 144.98, 143.55, 135.91, 131.51, 130.52, 130.11, 129.62, 129.04, 128.78, 128.20, 126.90, 126.60, 125.22 (q, J = 6.4 Hz), 124.85 123.26, 92.73, 55.99 (q, J = 25.5 Hz), 22.16; HRMS (ESI-TOF): $[M+Na]^+$ m/z calcd for $C_{23}H_{18}F_3NO_2Na^+$: 420.1187, found: 420.1200.

3-hydroxy-6-methyl-2-phenyl-3-(2,2,2-trifluoro-1-phenylethyl)isoindolin-1-one (3p)

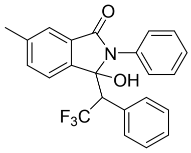

Diastereomeric ratio (major: minor = 10:1) was determined by ^{19}F NMR analysis of the unpurified product mixture. Faint yellow solid, 330 mg (83%); ^{1}H NMR (400 MHz, DMSO-d6) δ 7.90 (s, 1H), 7.80 (d, J = 8.0 Hz, 1H), 7.65 (d, J = 7.5 Hz, 1H), 7.53 (s, 1H), 7.40 (d, J = 5.3 Hz, 4H), 7.34 – 7.32 (m, 1H), 7.22 (t, J = 7.3 Hz, 1H), 7.06 (t, J = 7.6 Hz, 2H), 6.43 (d, J = 7.6 Hz, 2H), 4.12 (q, J = 10.5 Hz, 1H), 2.46 (s, 3H); ^{19}F NMR (376 MHz, DMSO-d6) δ -59.42 (major), -60.07 (minor).; ^{13}C NMR (101 MHz, DMSO-d6) δ165.52, 141.89, 140.65, 135.93, 134.00, 132.31, 130.62, 130.08, 129.03, 128.77, 128.21, 127.06, 126.93, 126.58, 124.74 (q, J = 6.2 Hz), 123.51, 92.84, 56.04 (q, J = 25.6 Hz), 21.31; HRMS (ESI-TOF): $[M+Na]^+$ m/z calcd for $C_{23}H_{18}F_3NO_2Na^+$: 420.1187, found: 420.1181.

3-hydroxy-4-methyl-2-phenyl-3-(2,2,2-trifluoro-1-phenylethyl)isoindolin-1-one (3q)

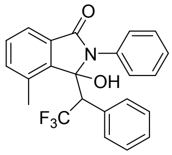

white solid, 254 mg (64%); ^{1}H NMR (400 MHz, DMSO-d6) δ 7.92 (s, 1H), 7.59 – 7.49 (m, 3H), 7.37 (d, J = 7.2 Hz, 2H), 7.30 – 7.21 (m, 3H), 7.13 (t, J = 7.4 Hz, 1H), 6.97 (t, J = 7.7 Hz, 2H), 6.64 (d, J = 7.5 Hz, 2H), 4.41 (q, J = 10.3 Hz, 1H), 2.70 (s, 3H); ^{19}F NMR (376 MHz, DMSO-d6) δ -60.35 (s); ^{13}C NMR (101 MHz, DMSO-d6) δ165.72, 143.58, 136.53, 135.90, 134.82, 134.08, 131.73, 131.09, 130.41,

130.18, 128.39, 128.20, 127.51, 126.78, 124.58(q, J = 6.7 Hz), 120.92, 92.32, 56.05 (q, J = 24.7 Hz), 18.17; HRMS (ESI-TOF): $[M+H]^+$ m/z calcd for $C_{23}H_{19}F_3NO_2^+$: 398.1368, found: 398.1365.

5-chloro-3-hydroxy-2-phenyl-3-(2,2,2-trifluoro-1-phenylethyl)isoindolin-1-one (3r)

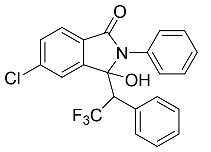

Diastereomeric ratio (major: minor = 10:1) was determined by ^{19}F NMR analysis of the unpurified product mixture. Faint yellow solid, 175 mg (42%); ^{11}H NMR (400 MHz, DMSO-d6) δ 8.16 (s, 1H), 7.87 (s, 1H), 7.75 (d, J = 9.8 Hz, 2H), 7.41 (d, J = 5.6 Hz, 4H), 7.33 (d, J = 2.6 Hz, 1H), 7.23 (t, J = 7.3 Hz, 1H), 7.08 (t, J = 7.5 Hz, 2H), 6.49 (d, J = 7.5 Hz, 2H), 4.20 (q, J = 10.4 Hz, 1H); ^{19}F NMR (376 MHz, DMSO-d6) δ -59.35 (major), -59.42 (minor).; ^{13}C NMR (101 MHz, DMSO-d6) δ163.90, 146.18, 137.61, 135.09, 130.69, 130.31, 129.73, 129.62, 128.67, 128.44, 127.88, 126.76, 126.58, 126.11, 124.83, 124.38 (q, J = 6.8 Hz), 92.01, 55.30 (q, J = 25.9 Hz); HRMS (ESI-TOF): $[M+H]^+$ m/z calcd for $C_{22}H_{16}ClF_3NO_2^+$: 418.0822, found: 418.0835.

6-chloro-3-hydroxy-2-phenyl-3-(2,2,2-trifluoro-1-phenylethyl)isoindolin-1-one (3s)

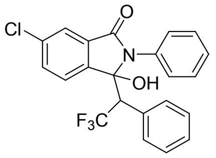

Diastereomeric ratio (major: minor = 10:1) was determined by ^{19}F NMR analysis of the unpurified product mixture. white solid, 142 mg (34%); ^{1}H NMR (400 MHz, $CDCl_3$) δ 7.82 (dd, J = 8.2, 1.1 Hz, 1H), 7.65 (dd, J = 8.2, 2.0 Hz, 1H), 7.20 – 7.11 (m, 5H), 7.11 – 7.05 (m, 2H), 6.95 (t, J = 7.8 Hz, 2H), 6.21 (d, J = 7.7 Hz, 2H), 4.92 (s, 1H), 3.95 (q, J = 10.0 Hz, 1H). ^{19}F NMR (376 MHz, $CDCl_3$) δ -60.58 (major), -61.62 (minor). ^{13}C NMR (101 MHz, $CDCl_3$) δ165.07, 141.65, 136.79, 134.21, 133.13, 132.80, 129.27, 128.88, 128.77, 128.36, 128.26, 127.94, 127.00, 125.73 (q, J = 6.7 Hz), 125.62, 123.21, 93.13, 55.60 (q, J = 26.7 Hz); HRMS (ESI-TOF): $[M+Na]^+$ m/z calcd for $C_{22}H_{15}ClF_3NO_2Na^+$: 440.0641, found: 440.0631.

3-hydroxy-6-methoxy-2-phenyl-3-(2,2,2-trifluoro-1-phenylethyl)isoindolin-1-one (3t)

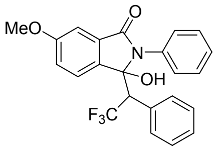

Diastereomeric ratio (major: minor = 10:1) was determined by ^{19}F NMR analysis of the unpurified product mixture. white solid, 343 mg (83%); ^{1}H NMR (400 MHz, $CDCl_3$) δ 7.74 (d, J = 8.3 Hz, 1H), 7.37 – 7.31 (m, 1H), 7.18 – 7.10 (m, 6H), 6.91 (t, J = 7.7 Hz, 2H), 6.66 (d, J = 2.4 Hz, 1H), 6.20 (d, J = 7.7 Hz, 2H), 4.95 (s, 1H), 3.94 (q, J = 10.1 Hz, 1H), 3.77 (s, 3H). ^{19}F NMR (376 MHz, $CDCl_3$) δ -60.63 (major), -61.85 (minor). ^{13}C NMR (101 MHz, $CDCl_3$) δ166.21, 161.18, 135.44, 134.82, 132.99, 129.72, 129.22, 129.01, 128.63, 128.25, 127.74, 126.54, 125.95, 125.51, 125.38 (q, J = 6.4 Hz), 106.12, 93.05, 55.65 (q, J = 26.4 Hz), 55.52; HRMS (ESI-TOF): $[M+Na]^+$ m/z calcd for $C_{23}H_{18}F_3NO_3Na^+$: 436.1136, found: 436.1131.

3-hydroxy-2-phenyl-3-(1,1,1-trifluorohexan-2-yl)isoindolin-1-one(3u)

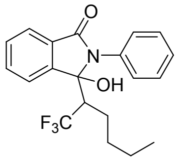

Diastereomeric ratio (major: minor = 1:1) was determined by ^{19}F NMR analysis of the unpurified product mixture. white solid, 309 mg (85%); ^{1}H NMR (400 MHz, $CDCl_3$) δ 7.68 (d, J = 7.7 Hz, 1H), 7.63 (d, J = 7.6 Hz, 1H), 7.59 (dd, J = 6.7, 1.1 Hz, 1H), 7.57 – 7.54 (m, 1H), 7.52 (d, J = 7.6 Hz, 1H), 7.43 (dd, J = 6.6, 3.2 Hz, 2H), 7.40 (dd, J = 6.1, 3.5 Hz, 2H), 7.33 – 7.27 (m, 5H), 7.25 – 7.19 (m, 3H), 7.15 (d, J = 7.5 Hz, 1H), 4.97 (major, 1H), 4.76 (minor, 1H), 2.74 – 2.59 (m, 2H), 2.36 (dt, J = 13.7, 6.0 Hz, 1H), 1.78 (ddd, J = 22.3, 15.0, 7.8 Hz, 2H), 1.11 – 0.76 (m, 12H), 0.67 (t, J = 7.2 Hz, 3H). ^{19}F NMR (376 MHz, $CDCl_3$) δ -61.30 (major), -65.54 (minor). ^{13}C NMR (101 MHz, $CDCl_3$) δ 167.03 (major), 166.93 (manor), 144.36 (major), 144.15 (minor), 134.71 (major), 134.69 (minor), 132.92 (major), 132.36 (minor), 130.76 (major), 130.29 (minor), 130.05 (major), 130.00 (minor), 128.92 (major), 128.72 (minor), 127.32 (major), 126.88 (minor), 126.63 (major), 126.40 (minor), 123.74 – 123.55 (m), 123.44 (major), 123.17 (minor), 93.39 (major, d, J = 1.7 Hz), 91.45 (minor, d, J = 2.0 Hz), 49.33 – 46.47 (m), 31.94 (major), 31.60 (minor), 30.37 (major), 29.71 (minor), 25.49 (major), 24.91 (minor), 22.63 (major), 22.60 (minor), 13.77 (major), 13.60 (minor); HRMS (ESI-TOF): $[M+Na]^+$ m/z calcd for $C_{20}H_{20}F_3NO_2Na^+$: 386.1344, found: 386.1350.

(3,3,3-trifluoroprop-1-ene-1,1-diyl)dibenzene

Oil, 75%, ^{1}H NMR (400 MHz, $CDCl_3$) δ 7.46 (dd, J = 6.1, 2.6 Hz, 3H), 7.43 – 7.37 (m, 3H), 7.33 (ddd, J = 6.2, 3.9, 2.1 Hz, 4H), 6.21 (q, J = 8.3 Hz, 1H). ^{19}F NMR (376 MHz, $CDCl_3$) δ -55.56 (s). ^{13}C NMR (101 MHz, $CDCl_3$) δ 152.69 (q, J = 5.5 Hz), 140.34 (s), 137.48 (s), 129.61 (s), 129.32 (q, J = 1.7 Hz), 128.70 (s), 128.69 (s), 128.25 (s), 128.18 (s), 123.32 (q, J = 270.6 Hz), 115.65 (q, J = 33.9 Hz).

5. References:

[1]. Li,L.; Wang,M.; Zhang,X,J.; Jiang,Y,W.; Ma,D,W. *Org. Lett.***2009**, *11*, 1309.

6. NMR spectra.

3a

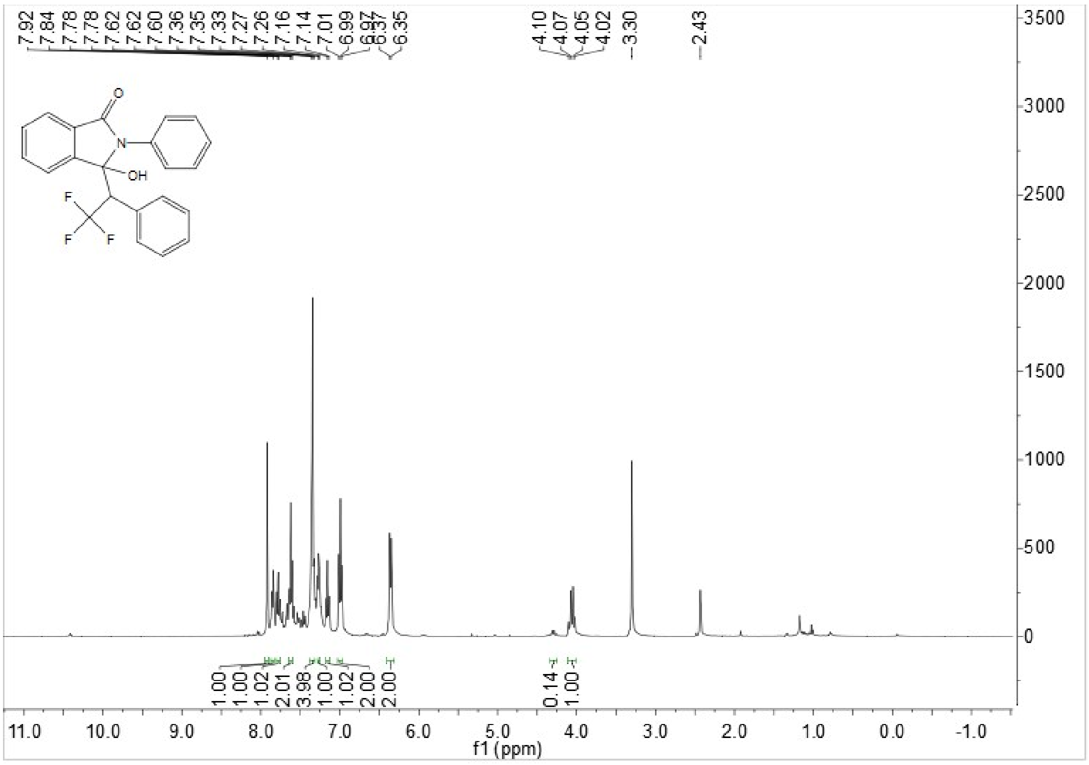

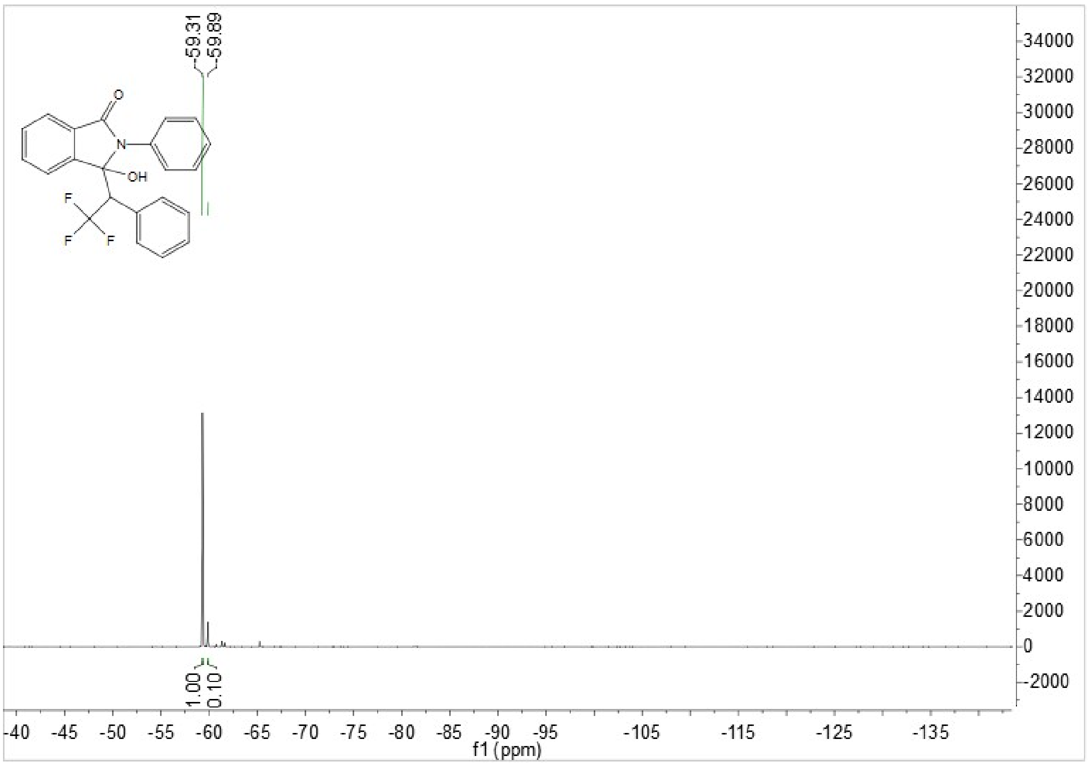

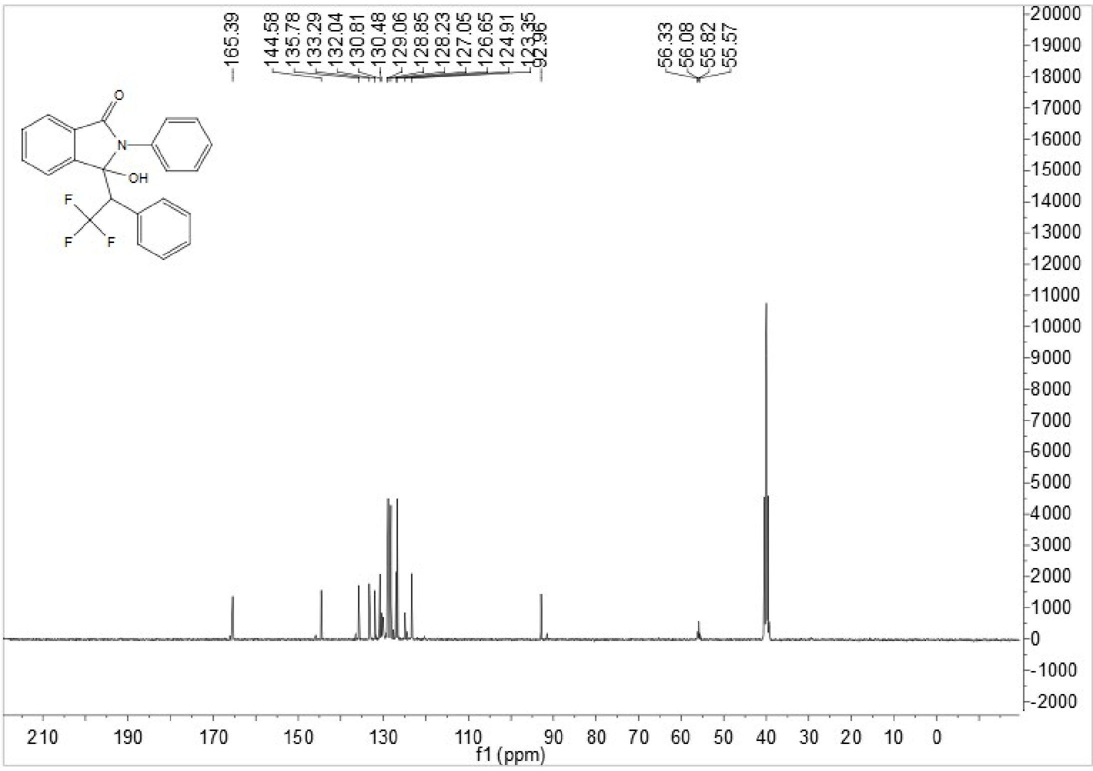

3b

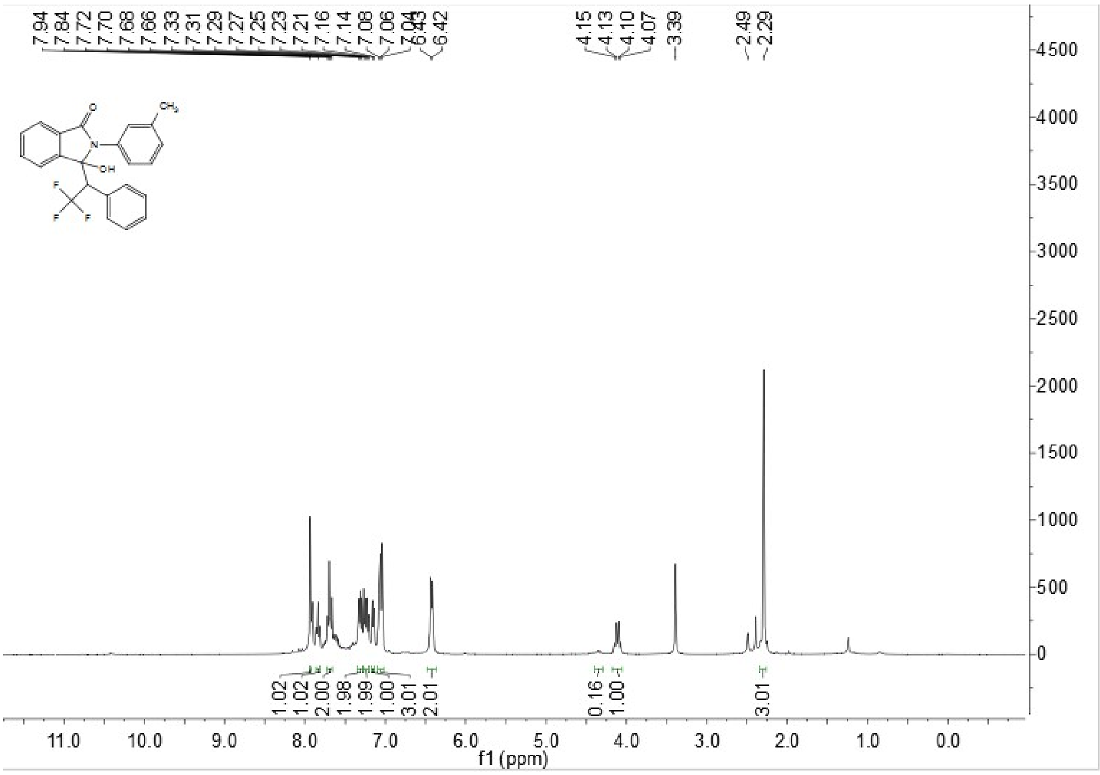

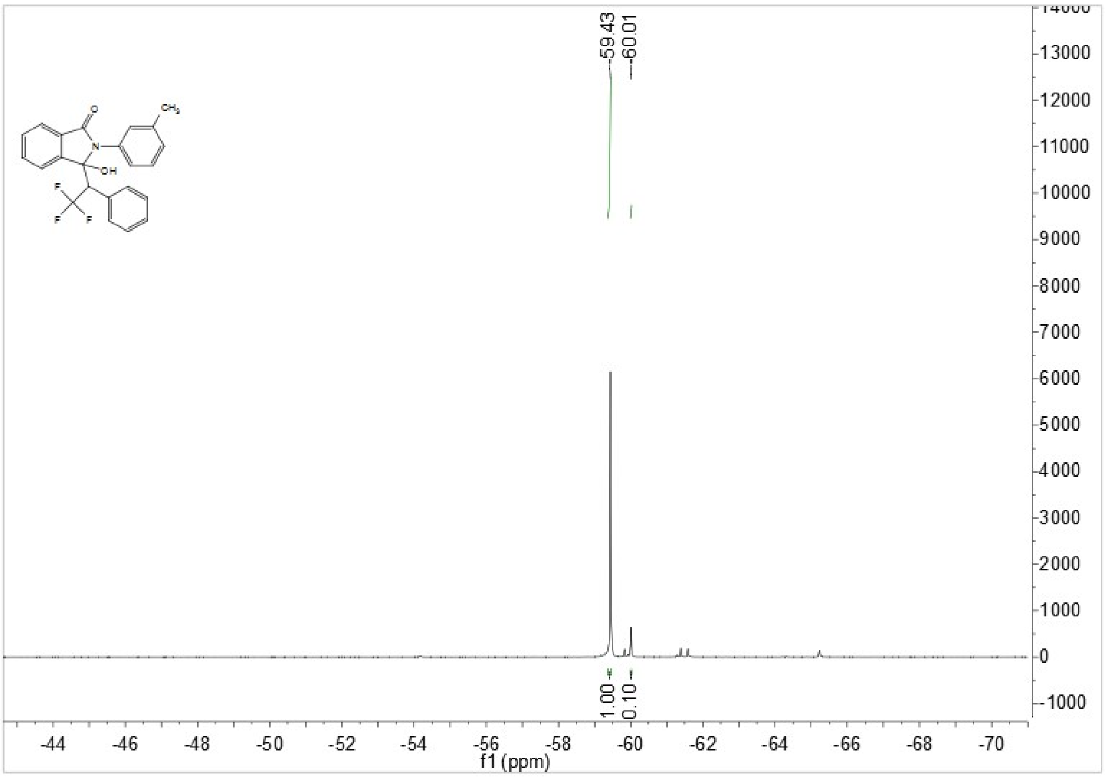
-59.43
-60.01
1.00
0.10
f1 (ppm)

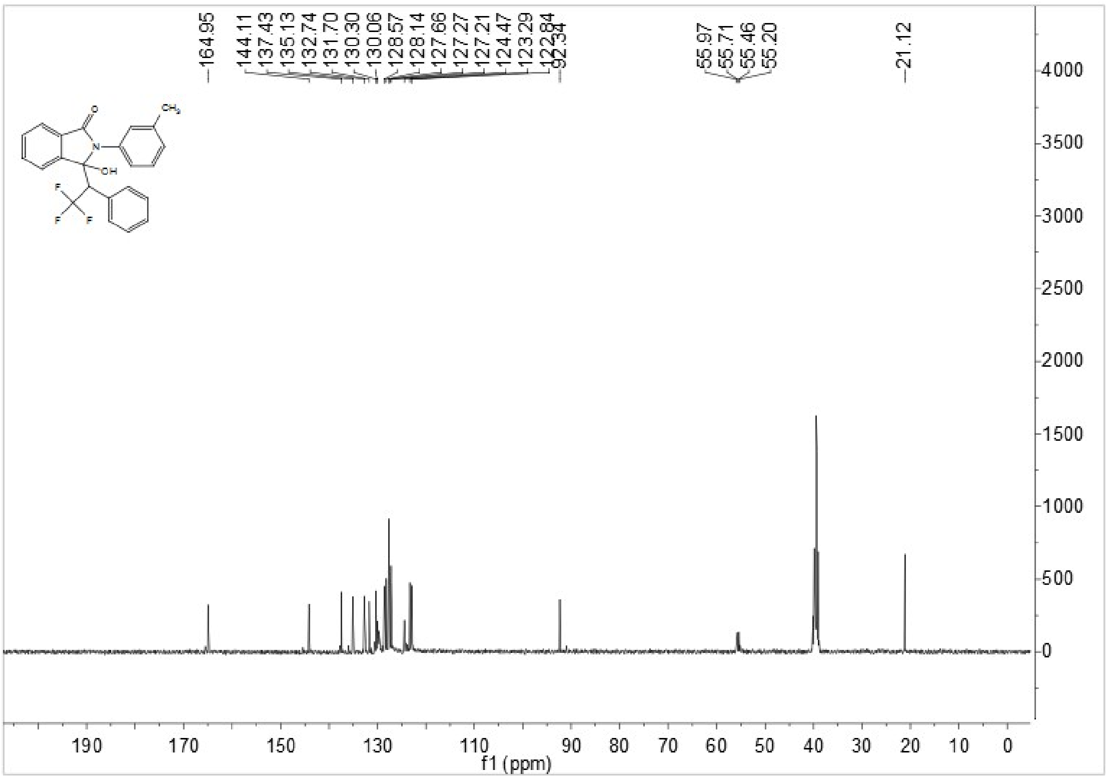
164.95
144.11
137.43
135.13
132.74
131.70
130.30
130.06
128.57
128.14
127.66
127.27
127.21
124.47
123.29
122.84
92.34
55.97
55.71
55.46
55.20
21.12
f1 (ppm)

3c

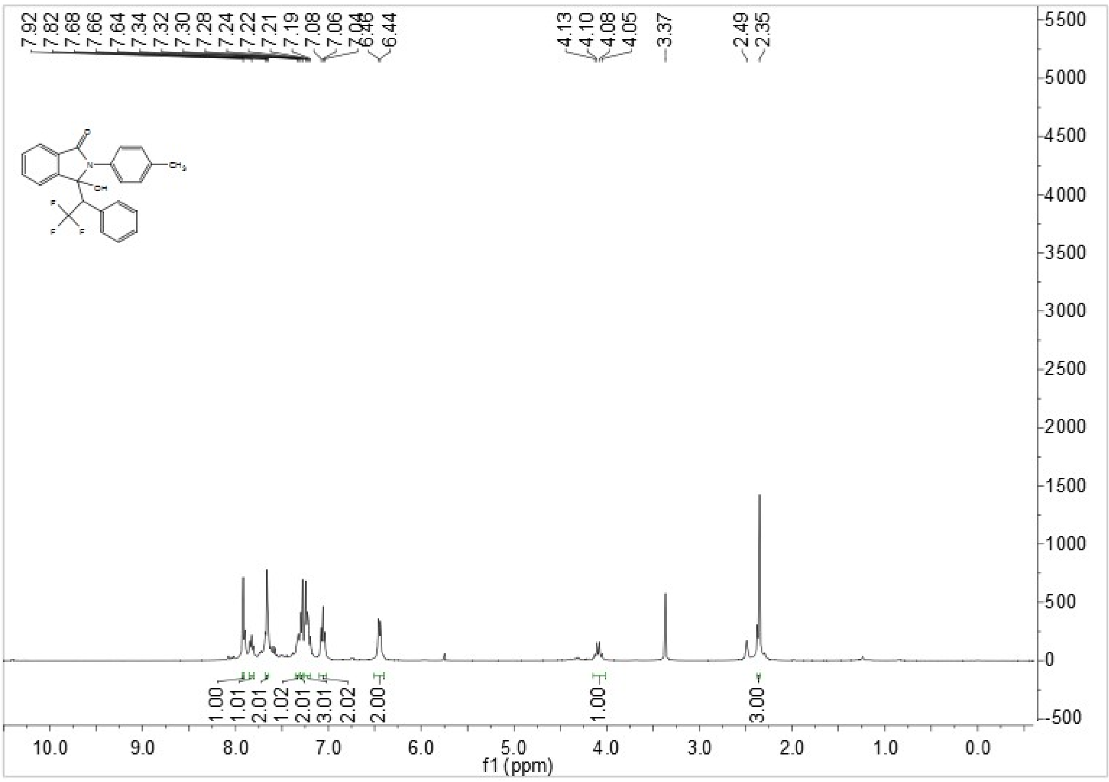

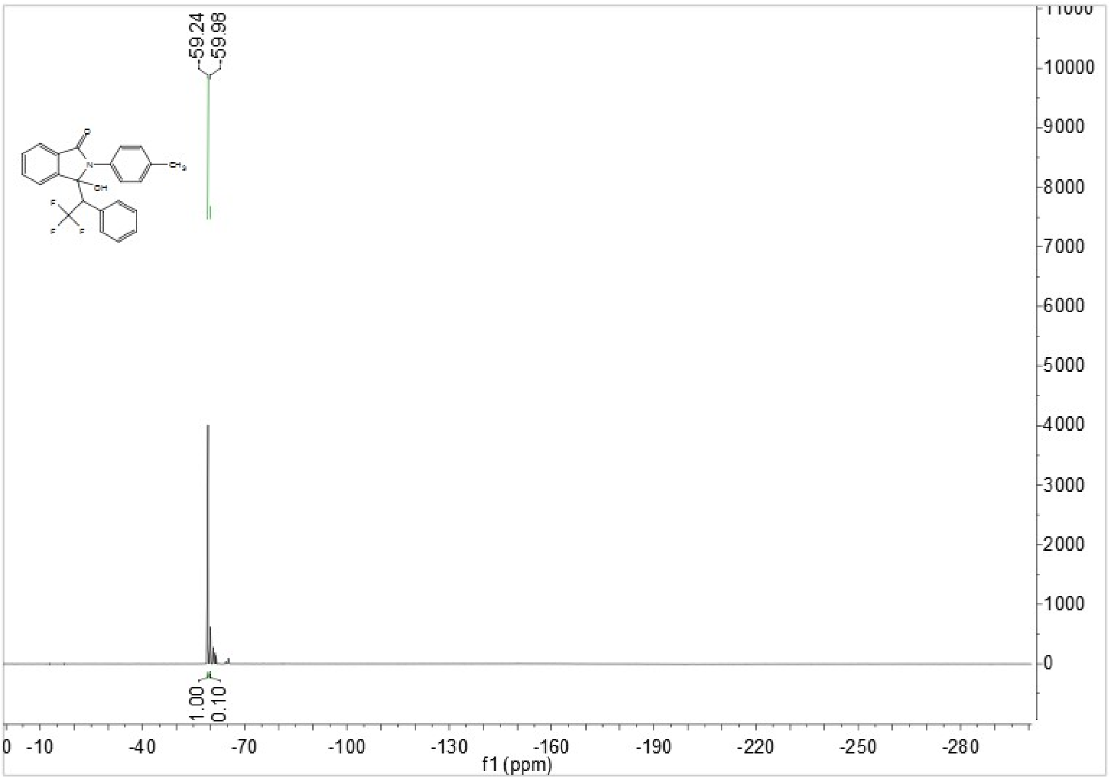

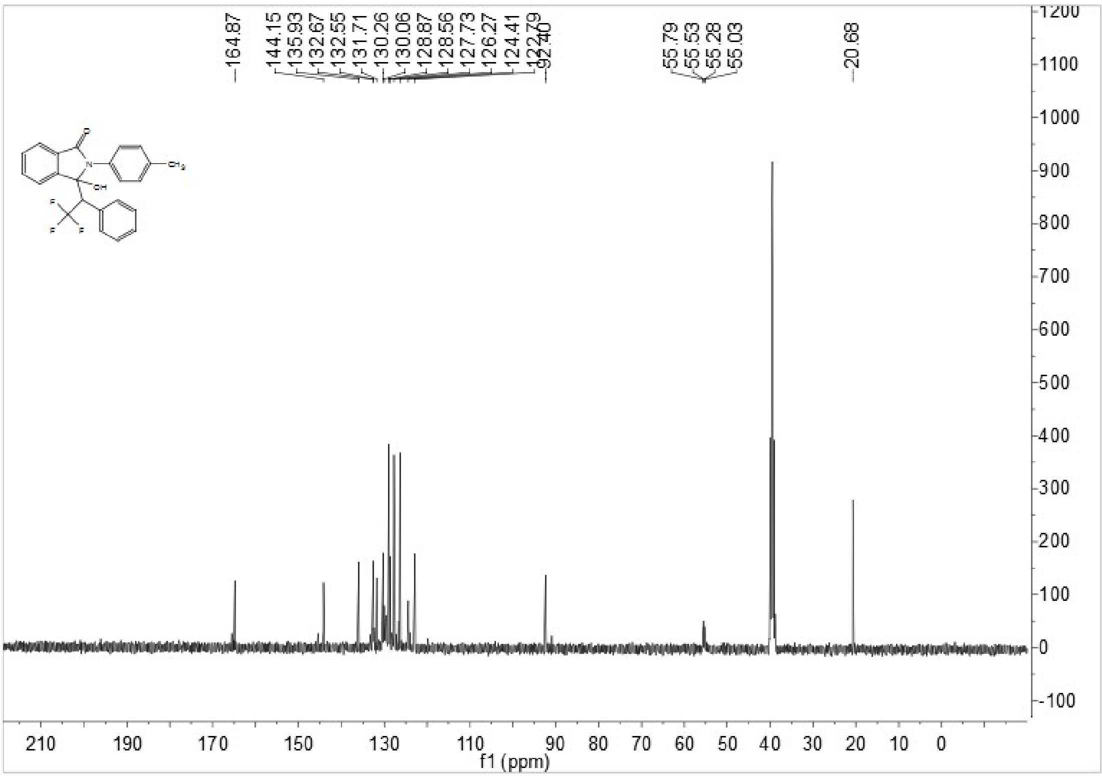

3d

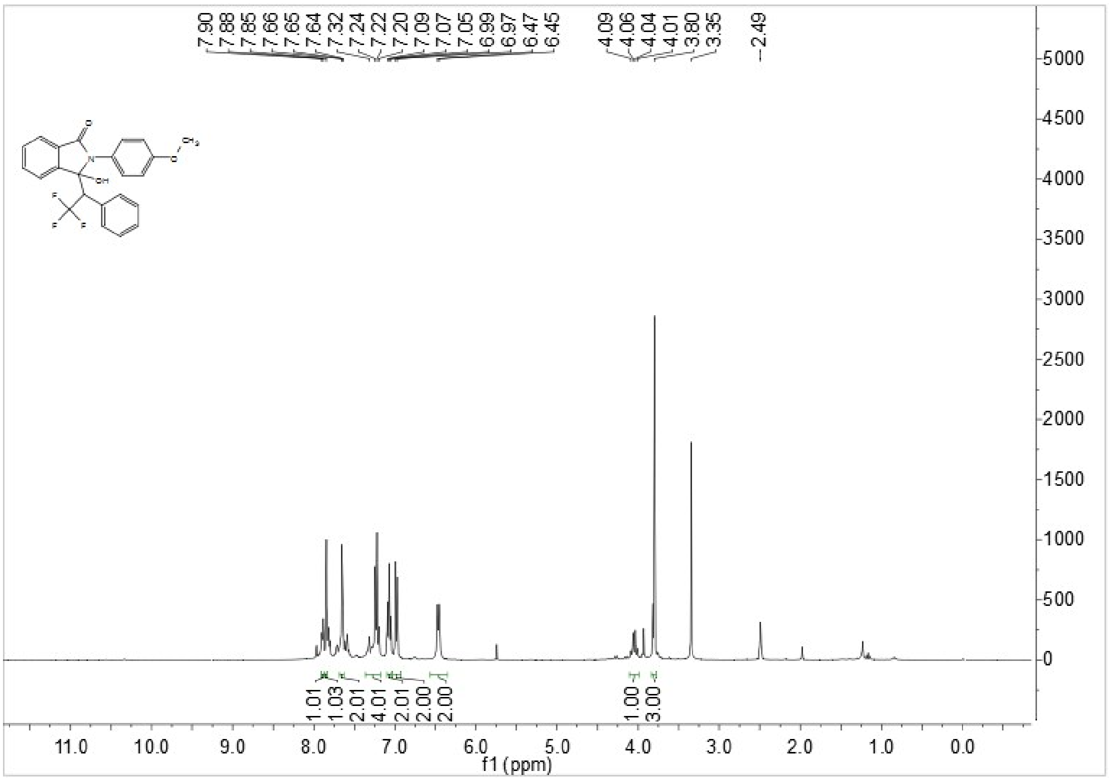

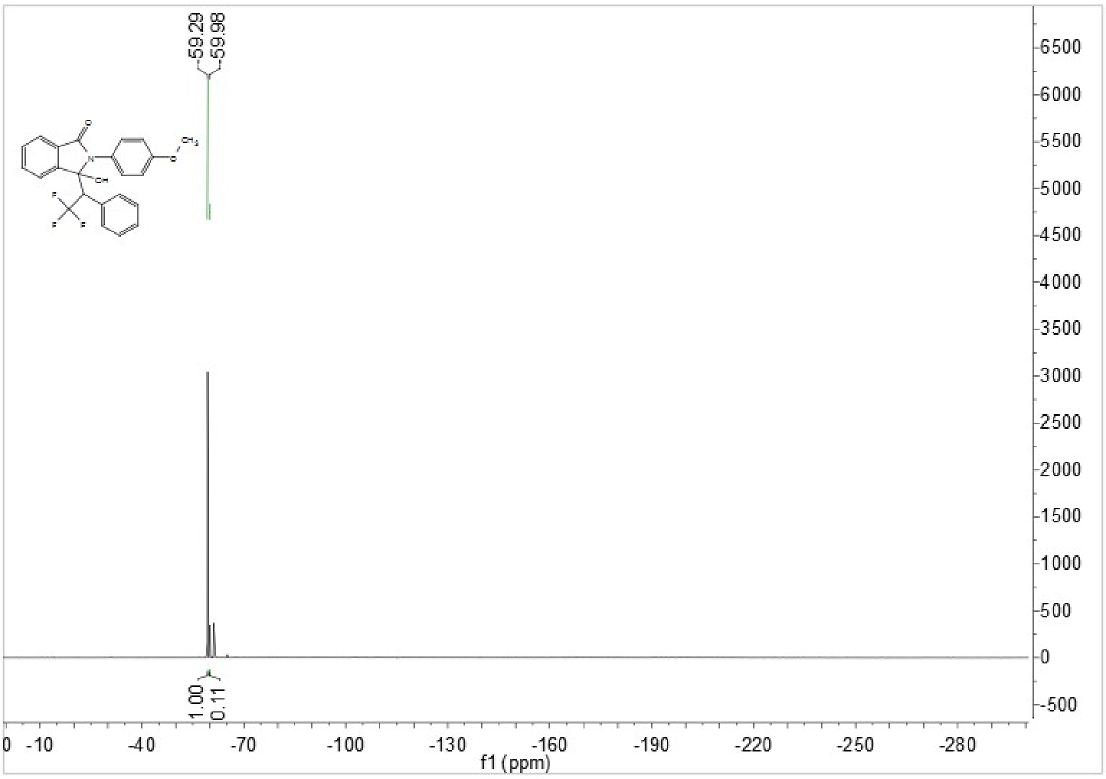
-59.29
-59.98
1.00
0.11
6500
6000
5500
5000
4500
4000
3500
3000
2500
2000
1500
1000
500
0
-500
0 -10 -40 -70 -100 -130 -160 -190 -220 -250 -280
f1 (ppm)

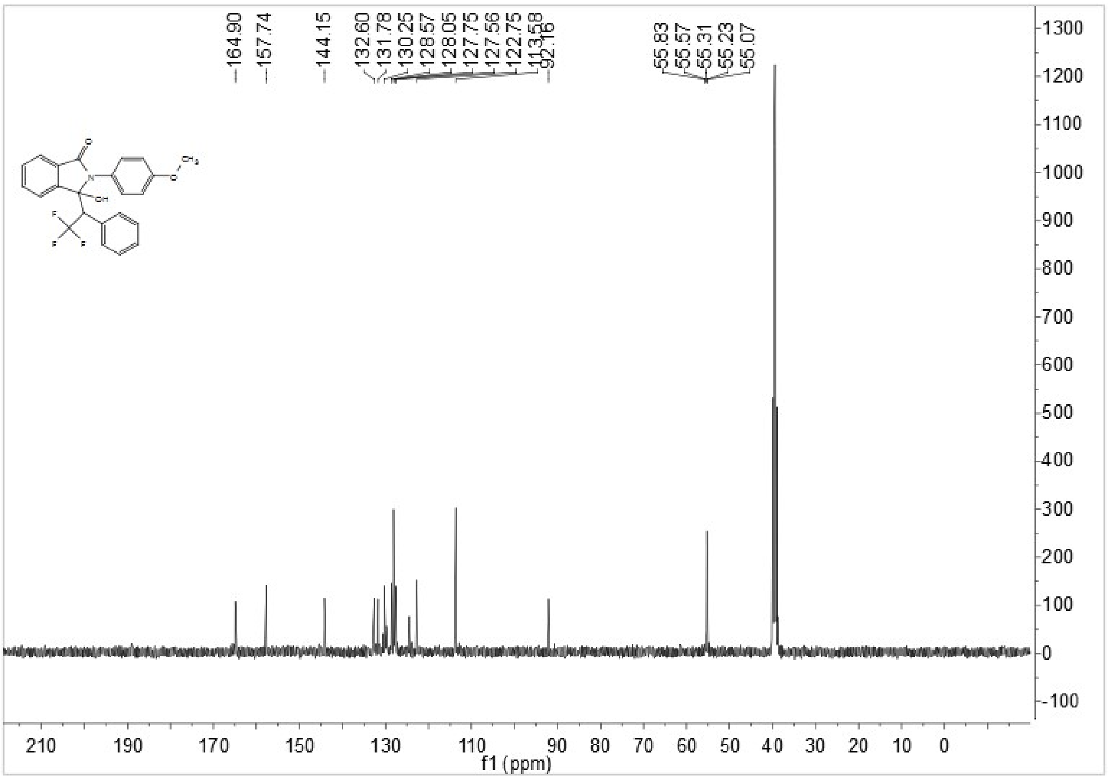
164.90
157.74
144.15
132.60
131.78
130.25
128.57
128.05
127.75
127.56
122.75
113.58
92.16
55.83
55.57
55.31
55.23
55.07
1300
1200
1100
1000
900
800
700
600
500
400
300
200
100
0
-100
210 190 170 150 130 110 90 80 70 60 50 40 30 20 10 0
f1 (ppm)

3e

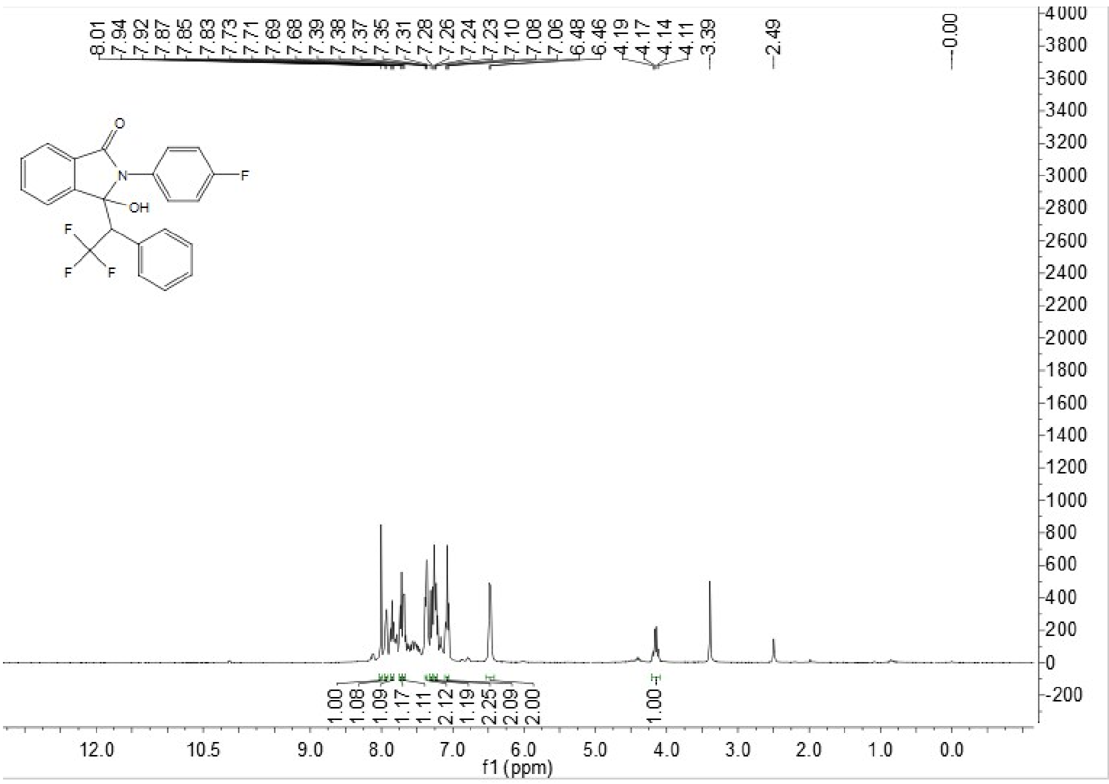

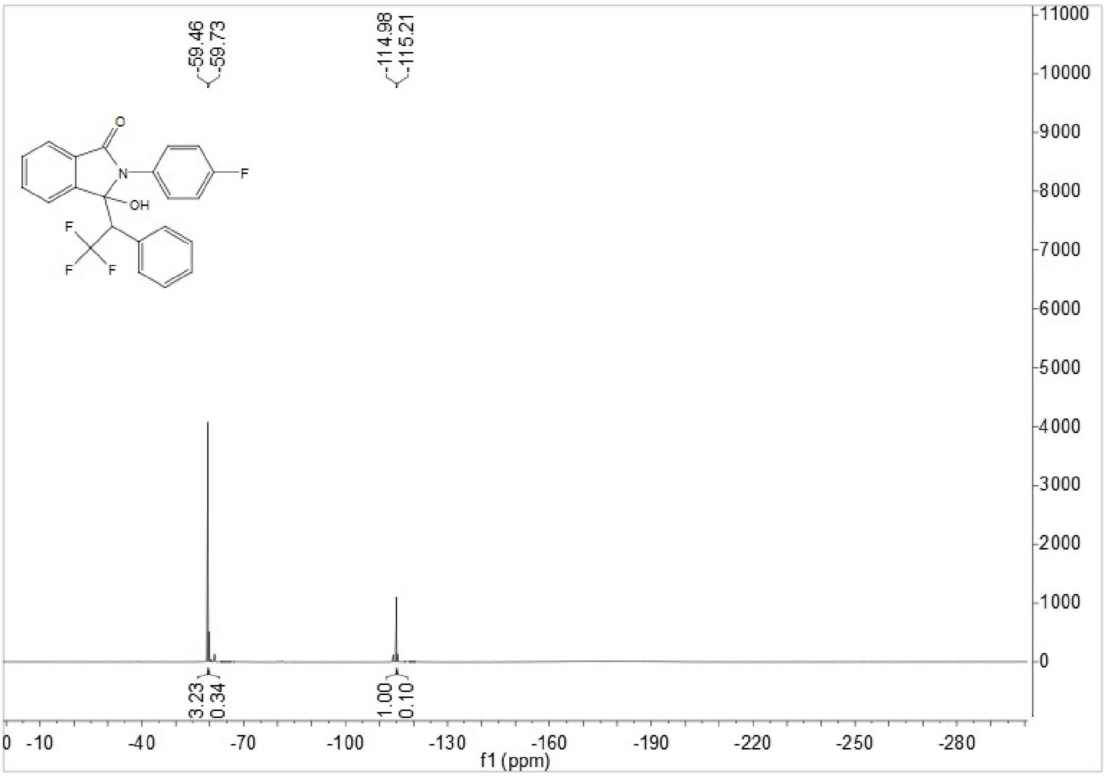

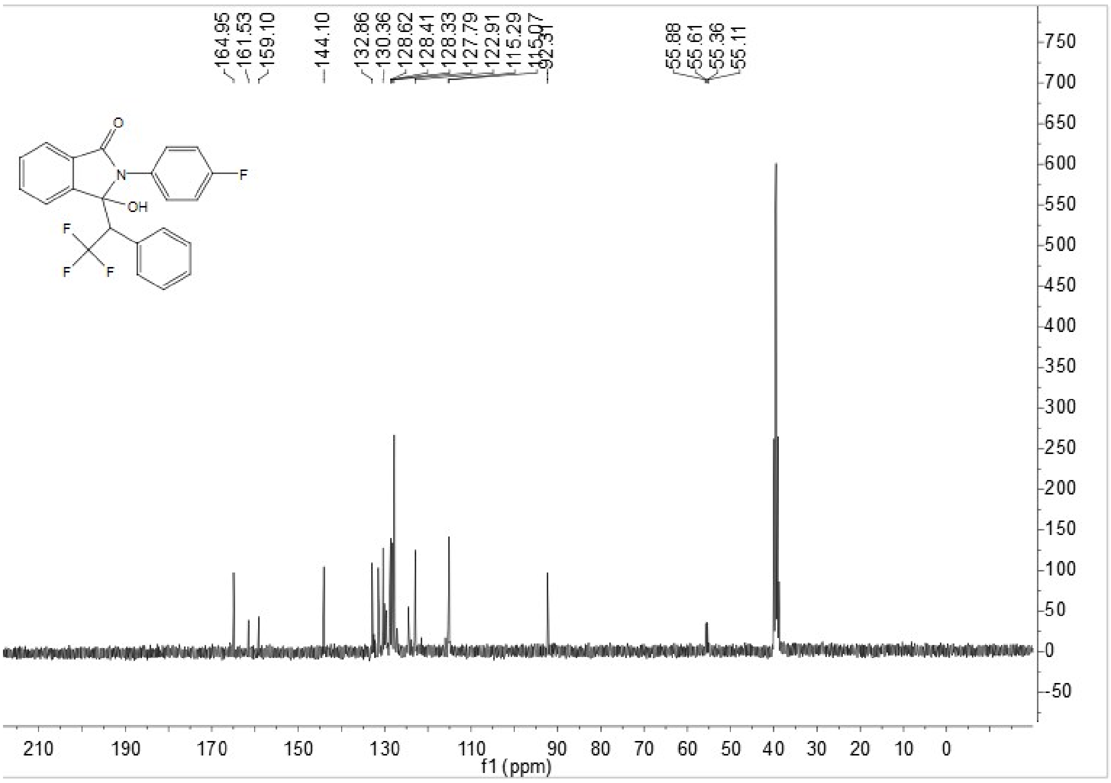
164.95
161.53
159.10
144.10
132.86
130.36
128.62
128.41
128.33
127.79
122.91
115.29
115.07
92.31
55.88
55.61
55.36
55.11
OH
N
F
f1 (ppm)

3f

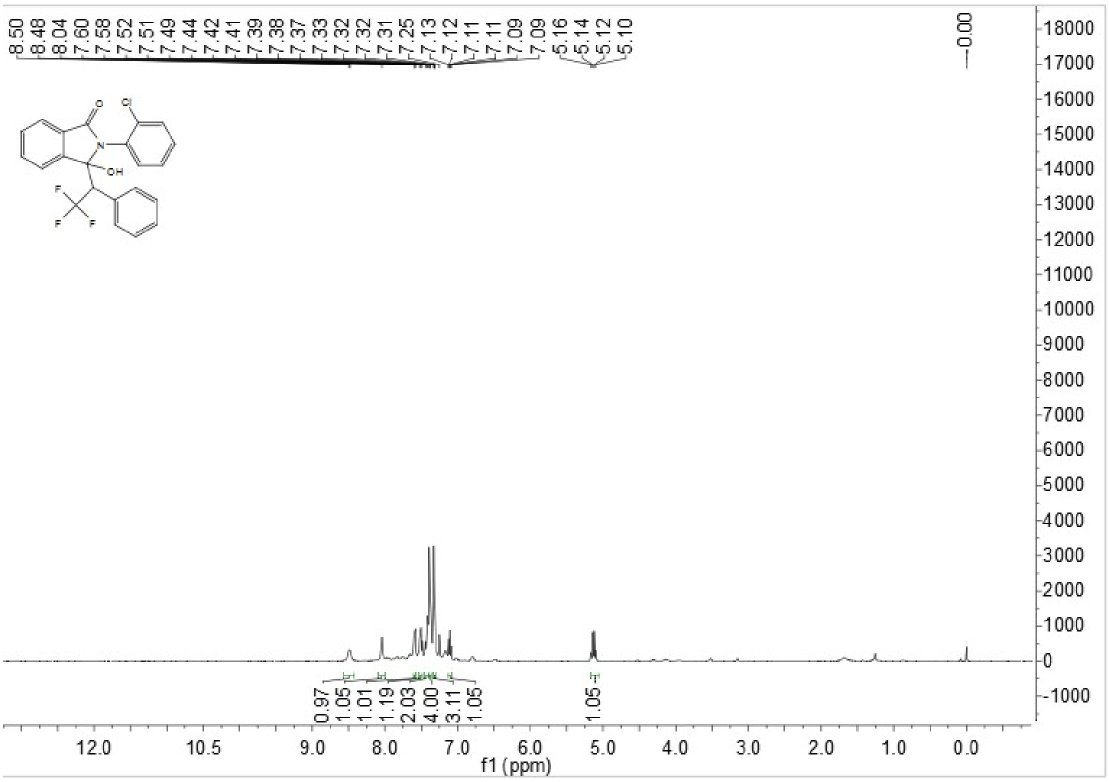
8.50
8.48
8.04
7.60
7.58
7.52
7.51
7.49
7.44
7.42
7.41
7.39
7.38
7.37
7.33
7.32
7.32
7.31
7.25
7.13
7.12
7.11
7.11
7.09
7.09
5.16
5.14
5.12
5.10
0.00
0.97
1.05
1.01
1.19
2.03
4.00
3.11
1.05
1.05
Cl
OH
f1 (ppm)

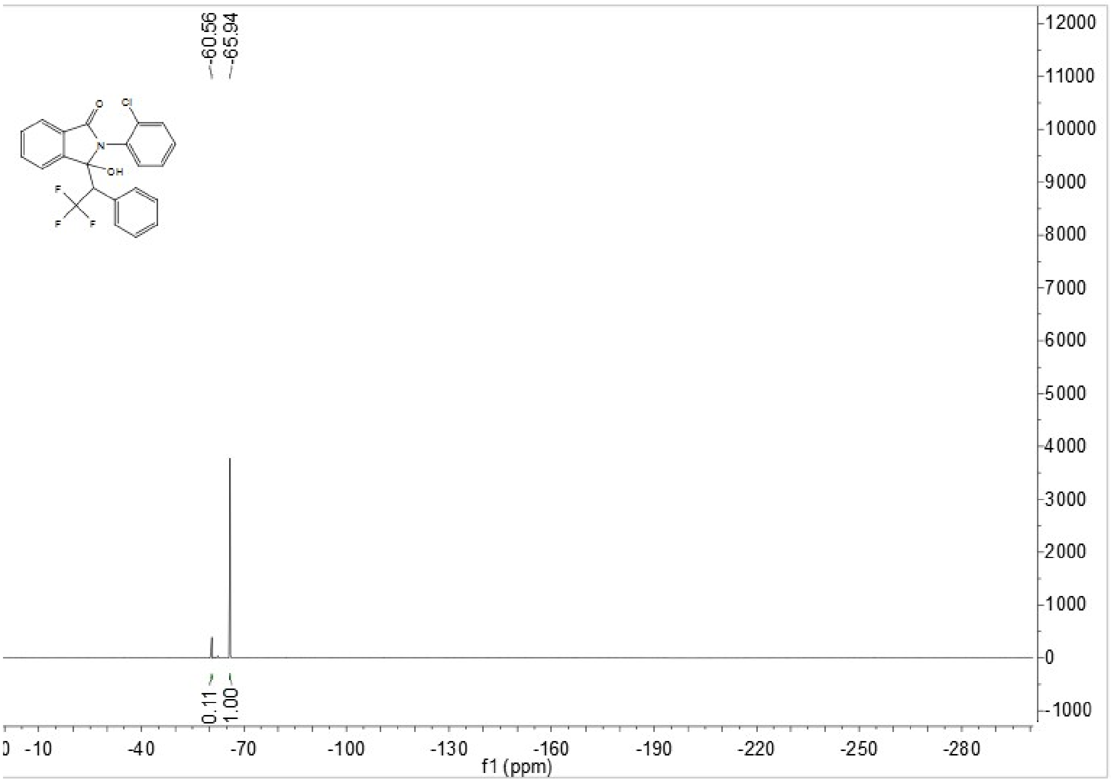
-60.56
-65.94
Cl
O
N
OH
F
F
F
0.11
1.00
f1 (ppm)

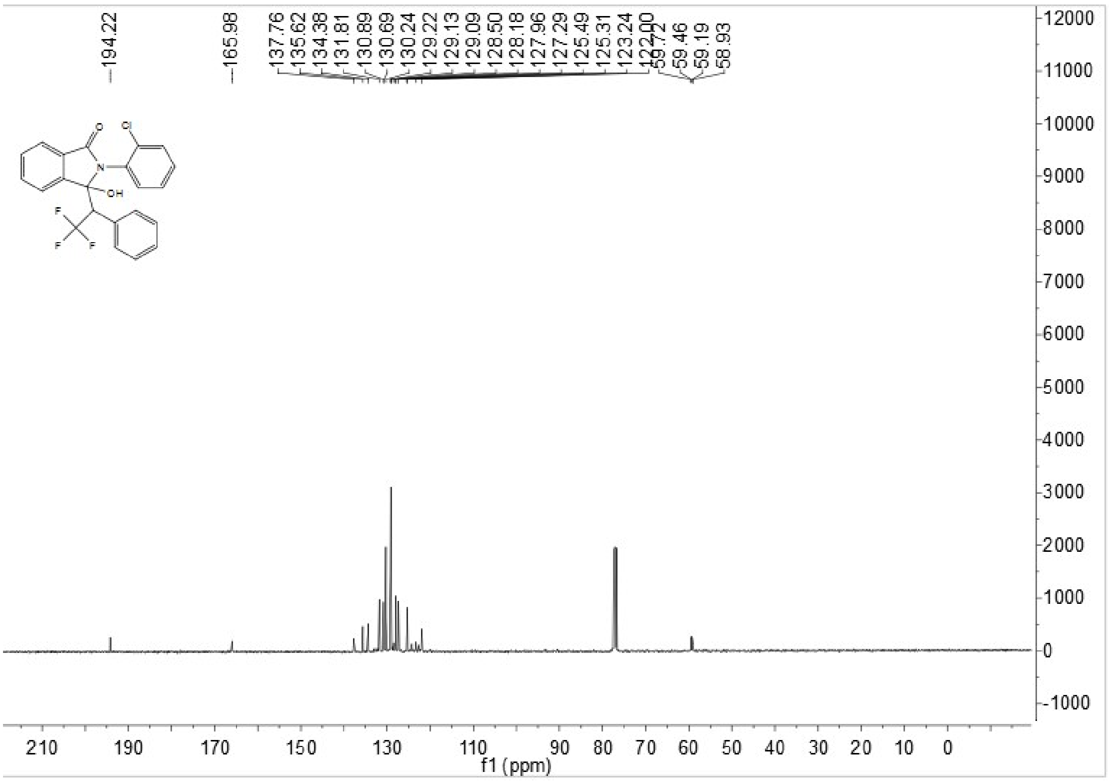
194.22
165.98
137.76
135.62
134.38
131.81
130.89
130.69
130.24
129.22
129.13
129.09
128.50
128.18
127.96
127.29
125.49
125.31
123.24
122.00
59.72
59.46
59.19
58.93
Cl
O
N
OH
F
F
F
f1 (ppm)

3g

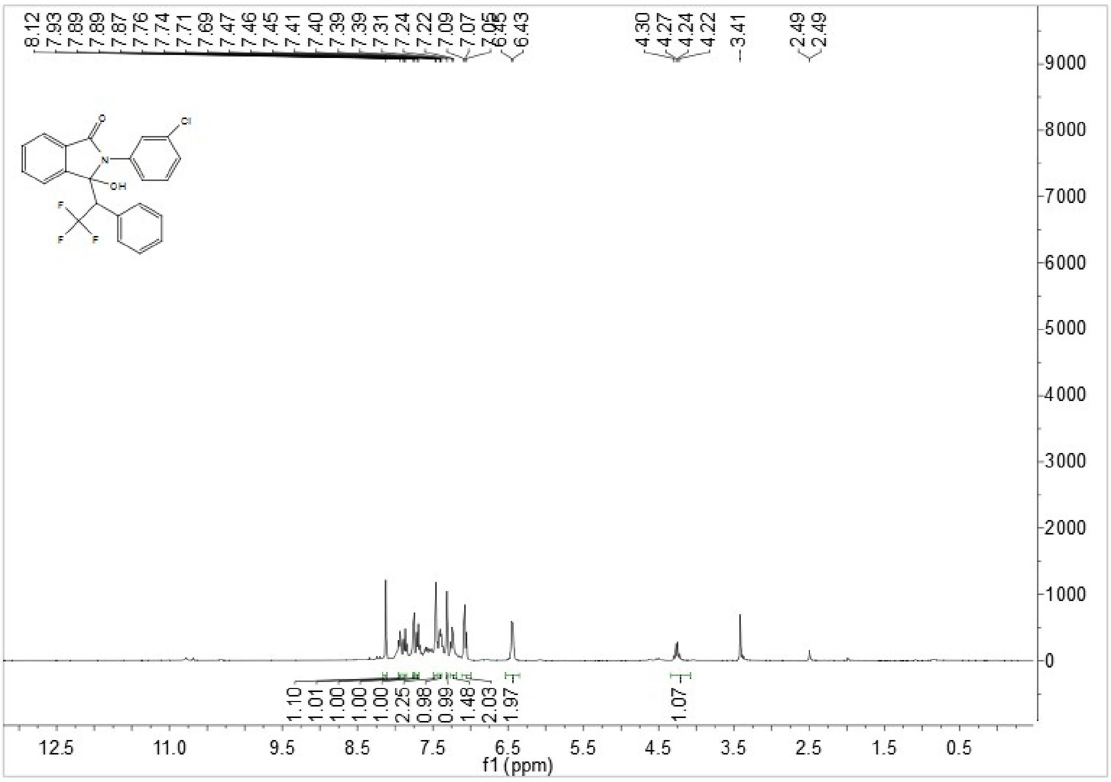

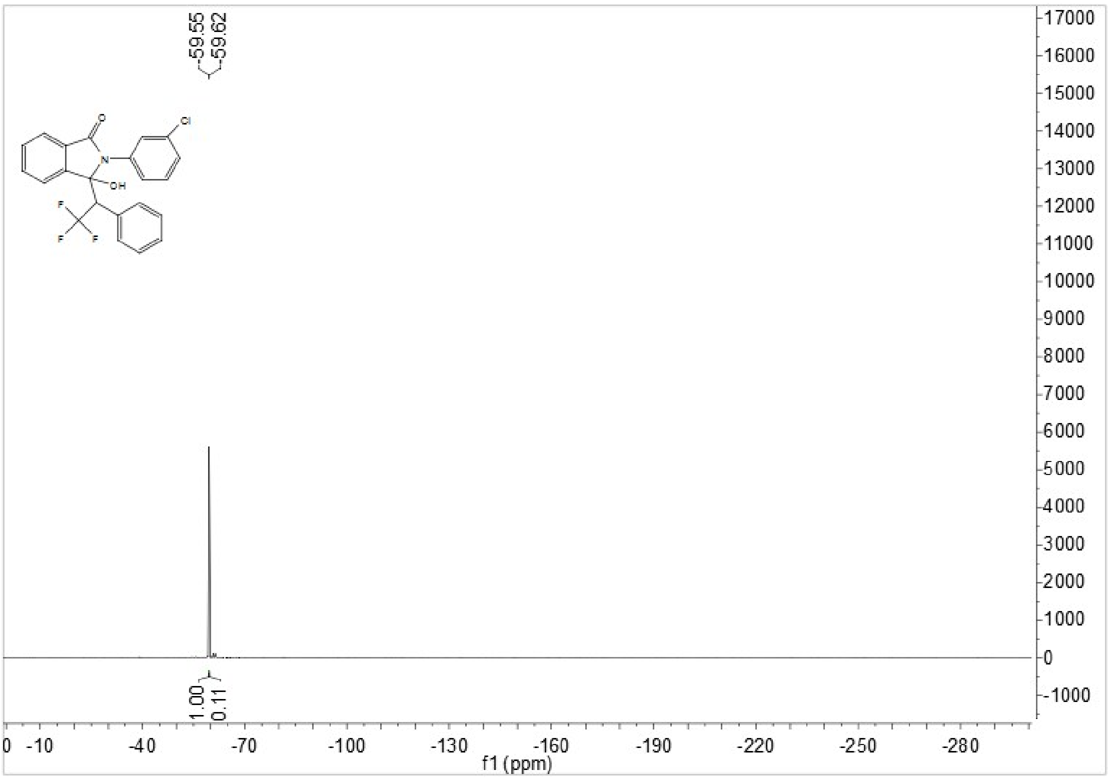

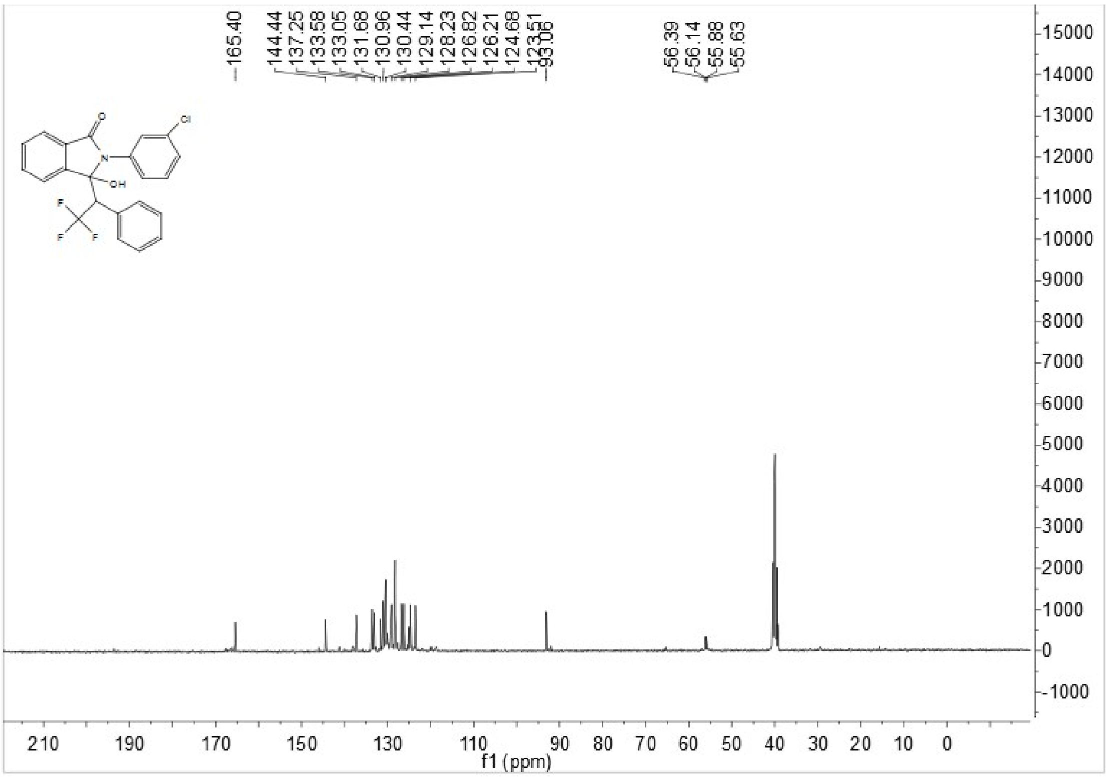
165.40
144.44
137.25
133.58
133.05
131.68
130.96
130.44
129.14
128.23
126.82
126.21
124.68
123.51
93.06
56.39
56.14
55.88
55.63
Cl
O
N
OH
F
F
F
f1 (ppm)

3h

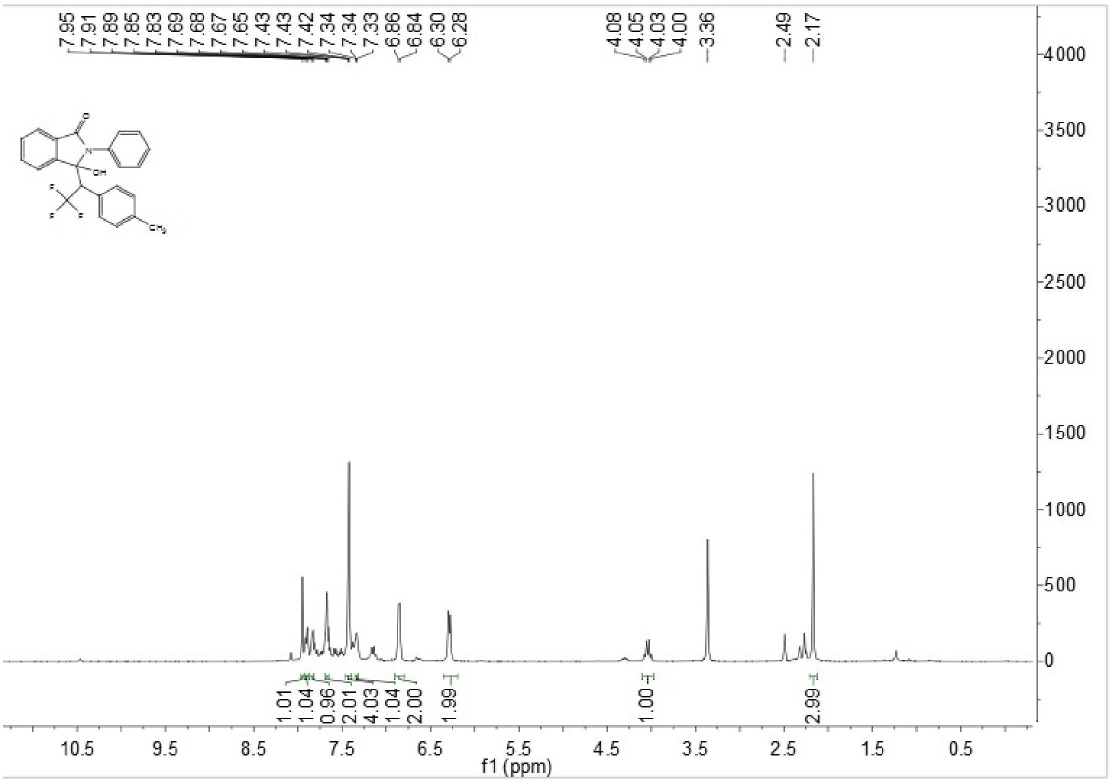
7.95
7.91
7.89
7.85
7.83
7.69
7.68
7.67
7.65
7.43
7.42
7.34
7.34
7.33
6.86
6.84
6.30
6.28
4.08
4.05
4.03
4.00
3.36
2.49
2.17
1.01
1.04
0.96
2.01
4.03
1.04
2.00
1.99
1.00
2.99
f1 (ppm)

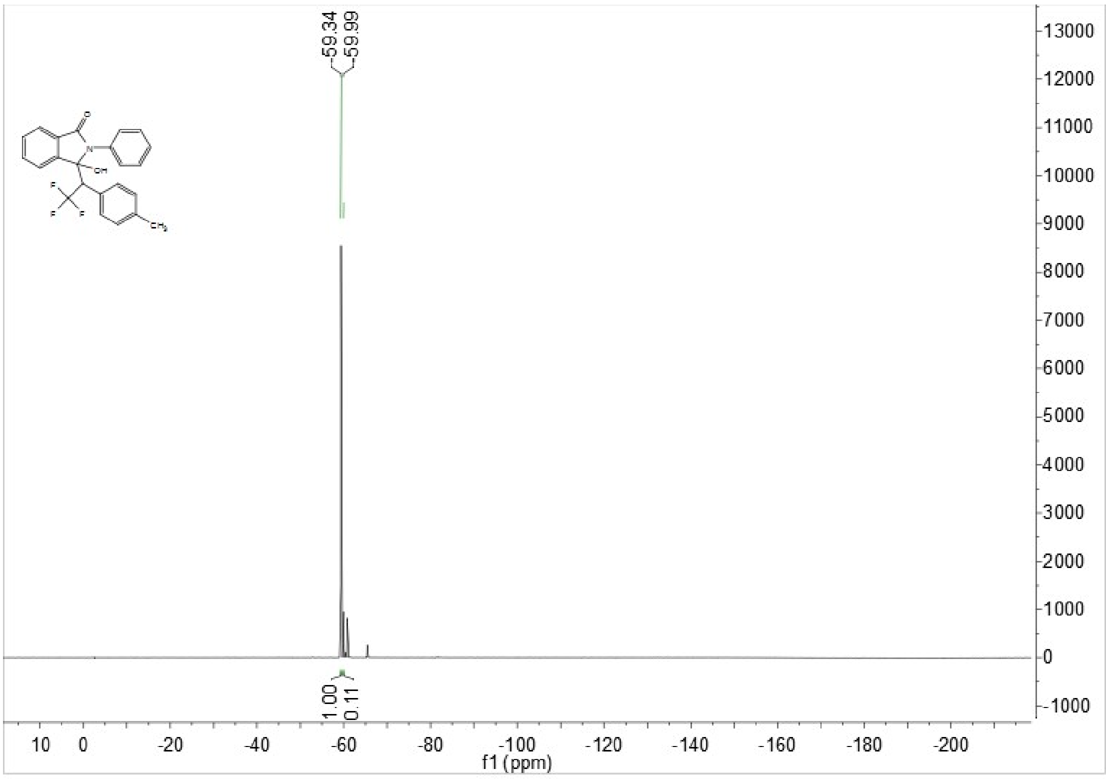
-59.34
-59.99
1.00
0.11
f1 (ppm)

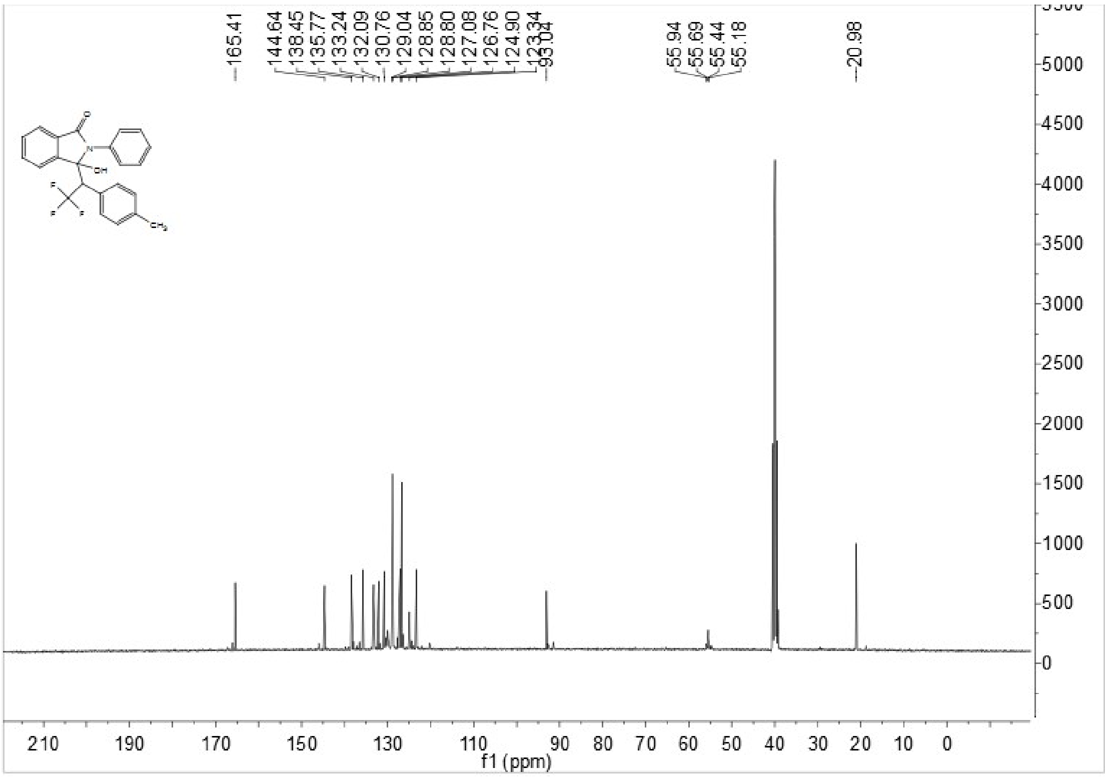
165.41
144.64
138.45
135.77
133.24
132.09
130.76
129.04
128.85
128.80
127.08
126.76
124.90
123.34
93.04
55.94
55.69
55.44
55.18
20.98
f1 (ppm)

3i

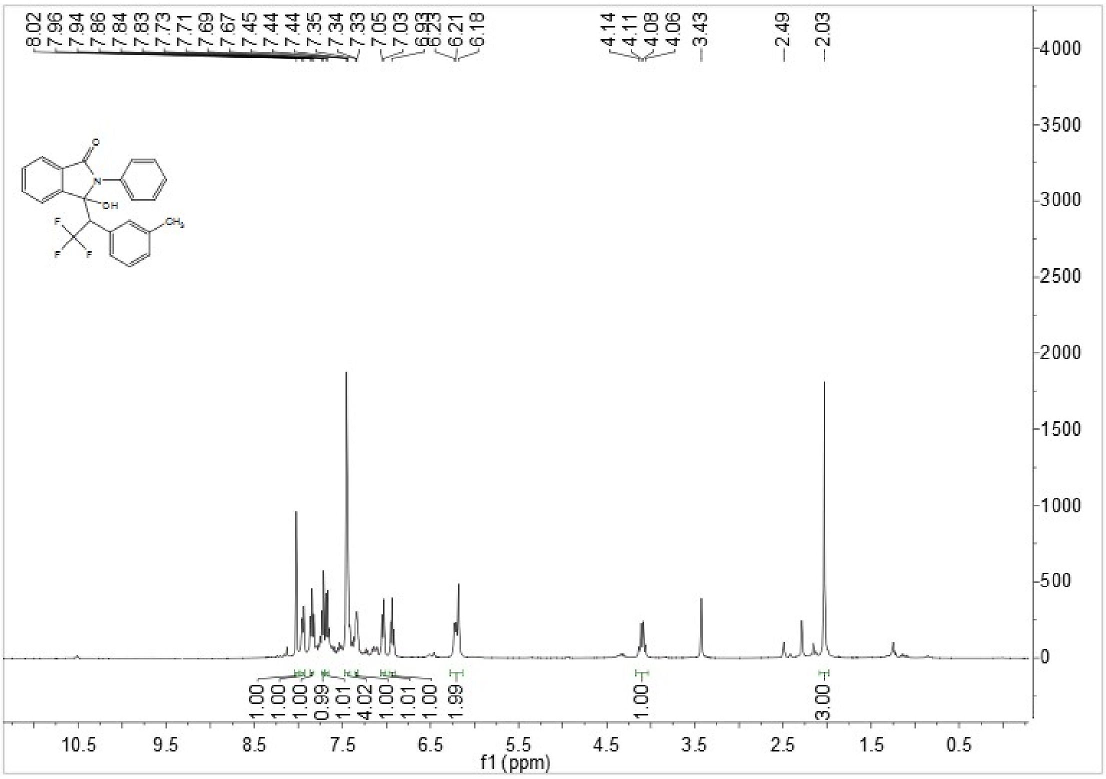
8.02 7.96 7.94 7.86 7.84 7.83 7.73 7.71 7.69 7.67 7.45 7.44 7.44 7.35 7.34 7.33 7.05 7.03 6.93 6.23 6.21 6.18
4.14 4.11 4.08 4.06 3.43
2.49
2.03
1.00 1.00 1.00 0.99 1.01 4.02 1.00 1.01 1.00 1.99
1.00
3.00
f1 (ppm)

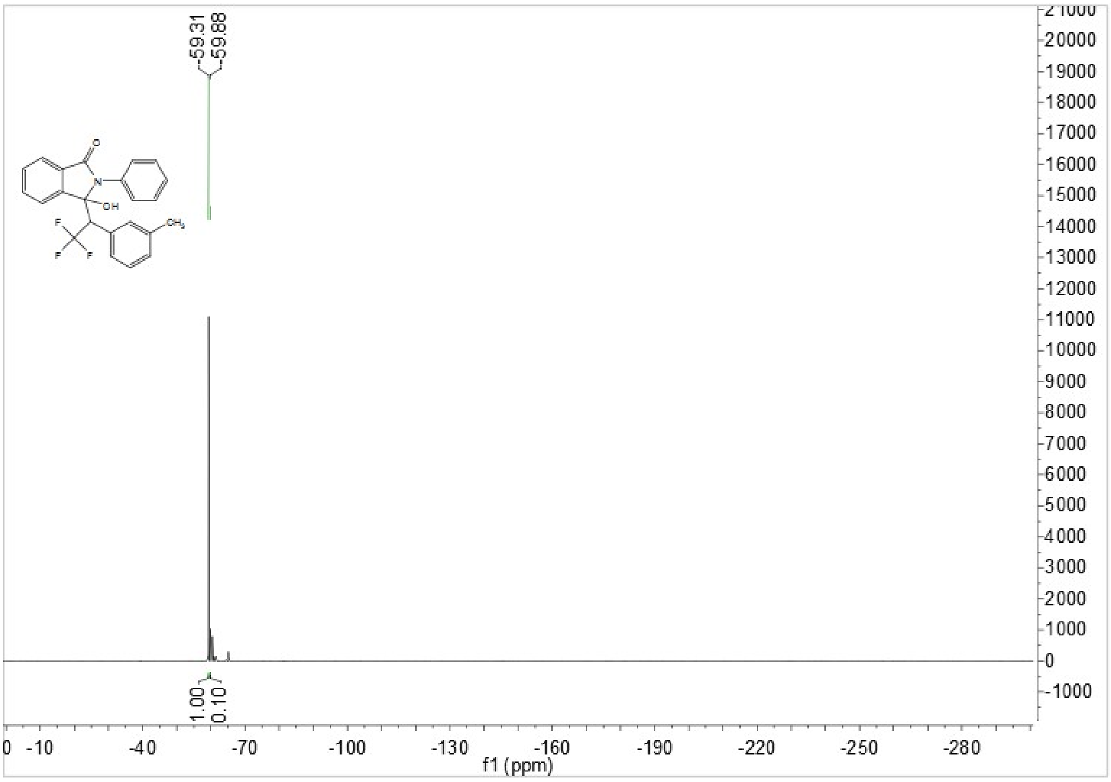
-59.31
-59.88
1.00
0.10
f1 (ppm)

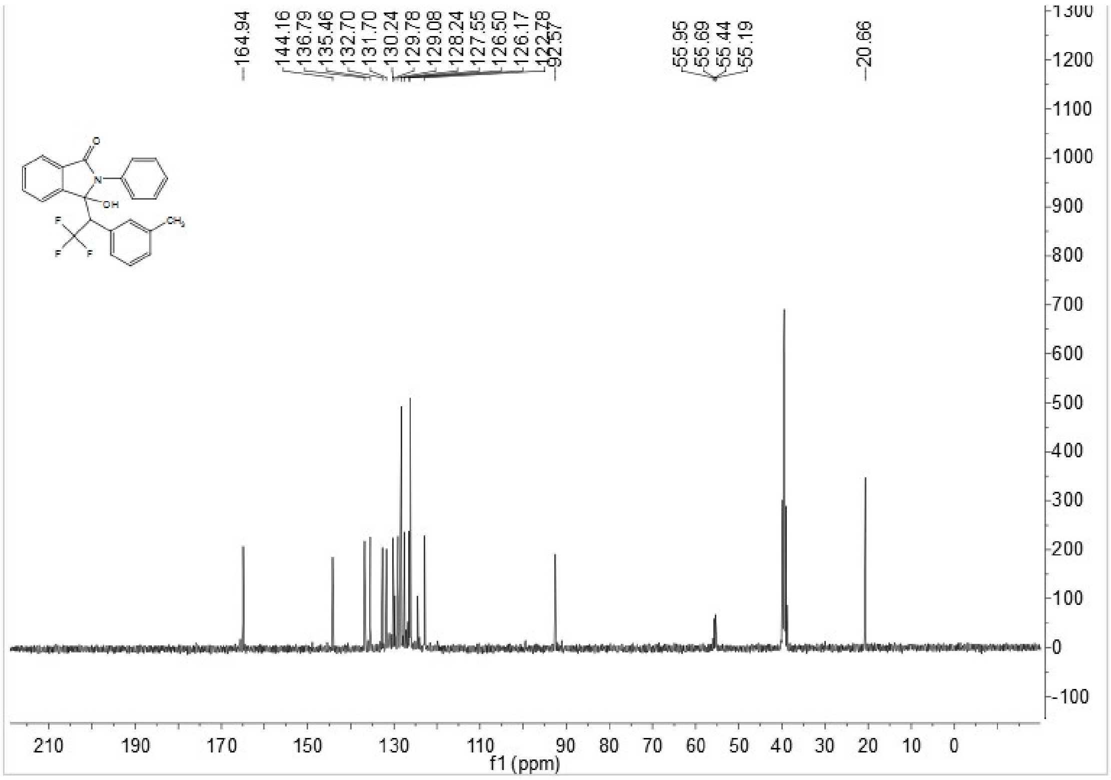

3j

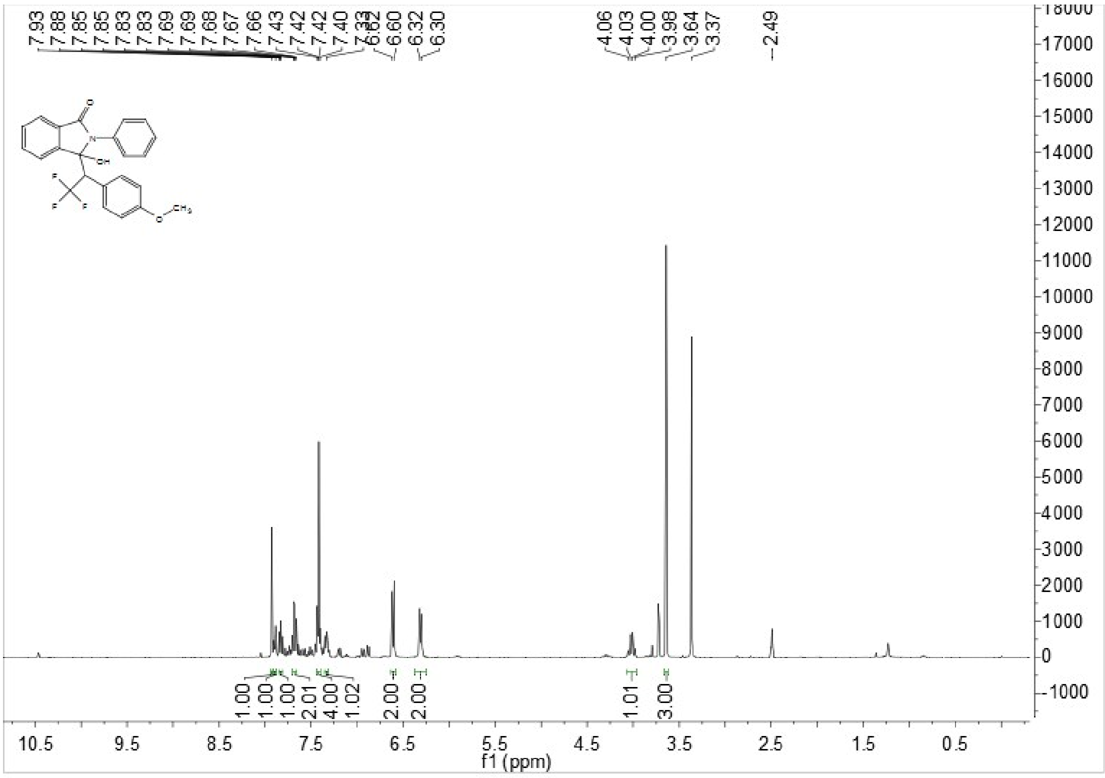

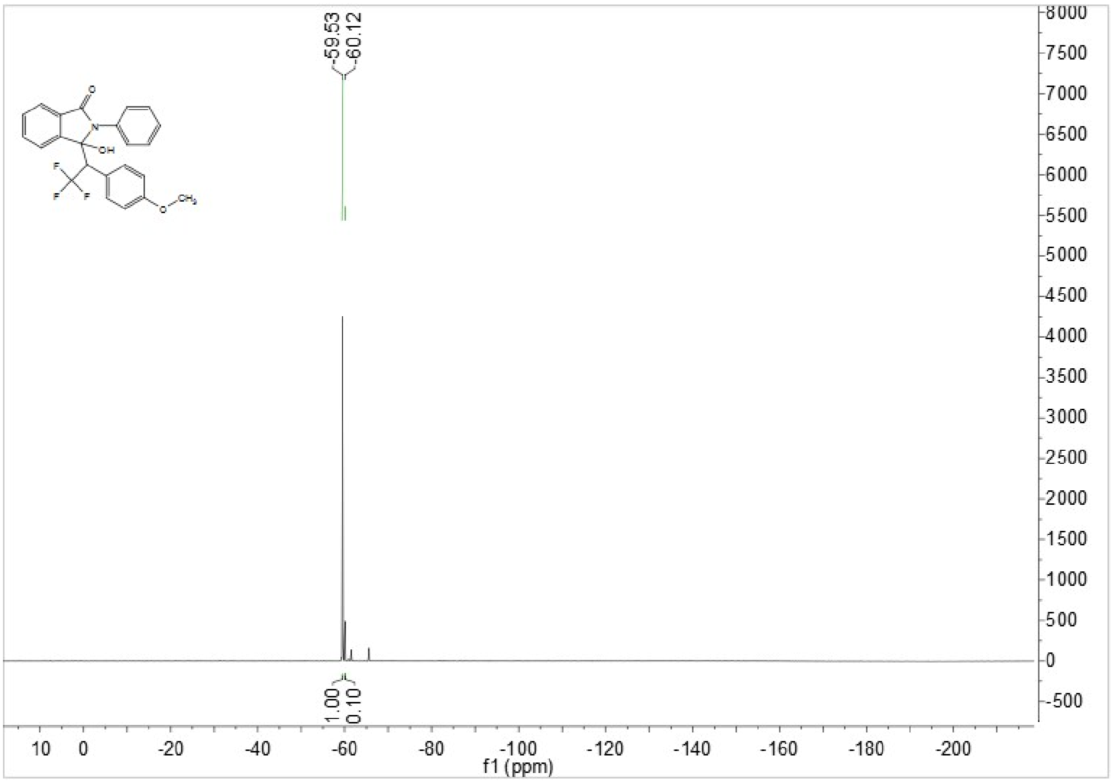

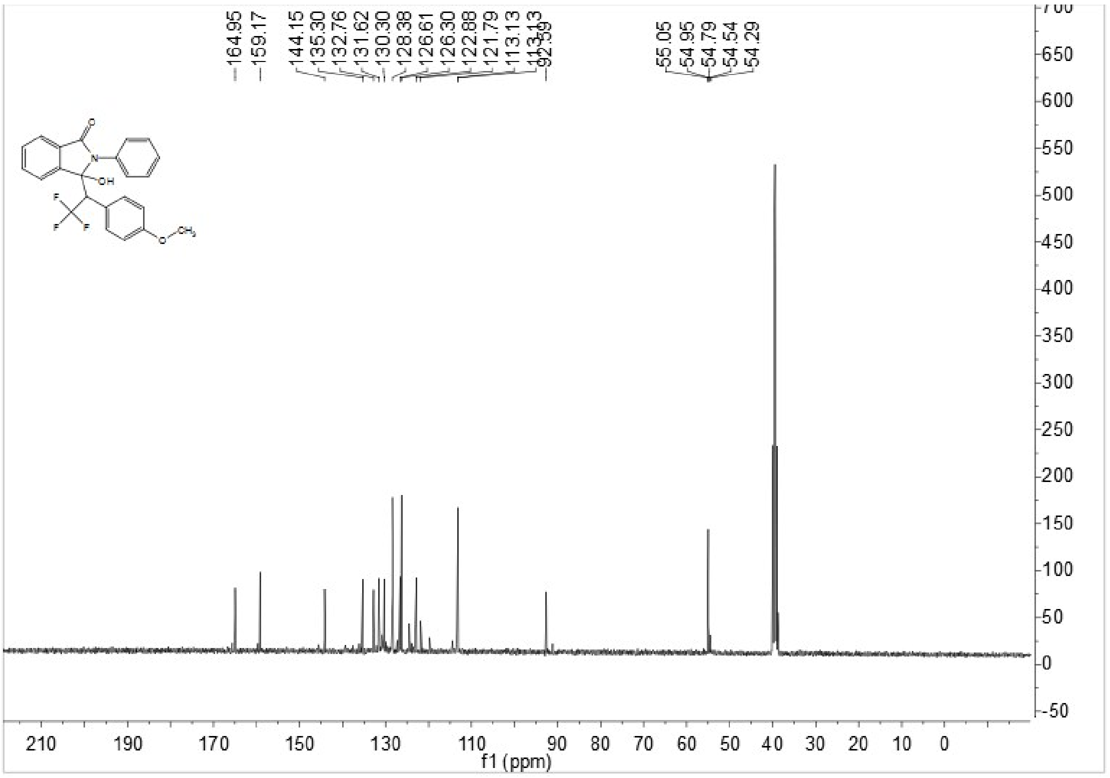

3k

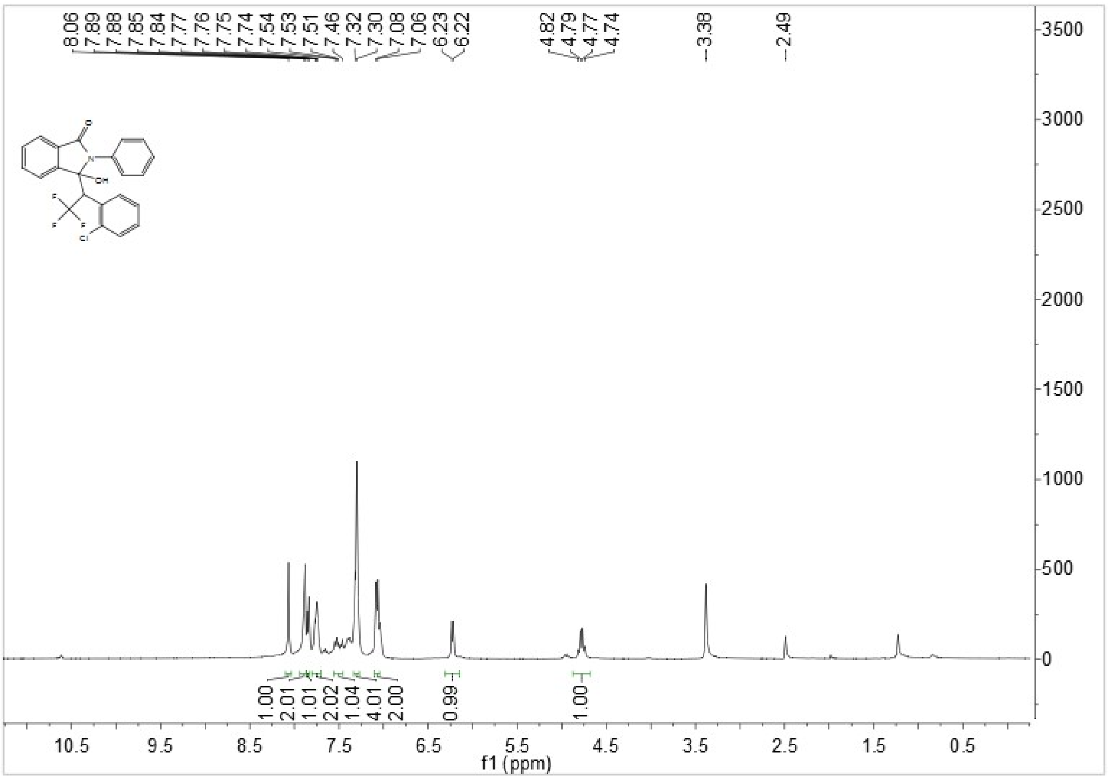
8.06
7.89
7.88
7.85
7.84
7.77
7.76
7.75
7.74
7.54
7.53
7.51
7.46
7.32
7.30
7.08
7.06
6.23
6.22
4.82
4.79
4.77
4.74
3.38
2.49
1.00
2.01
1.01
2.02
1.04
4.01
2.00
0.99
1.00
f1 (ppm)

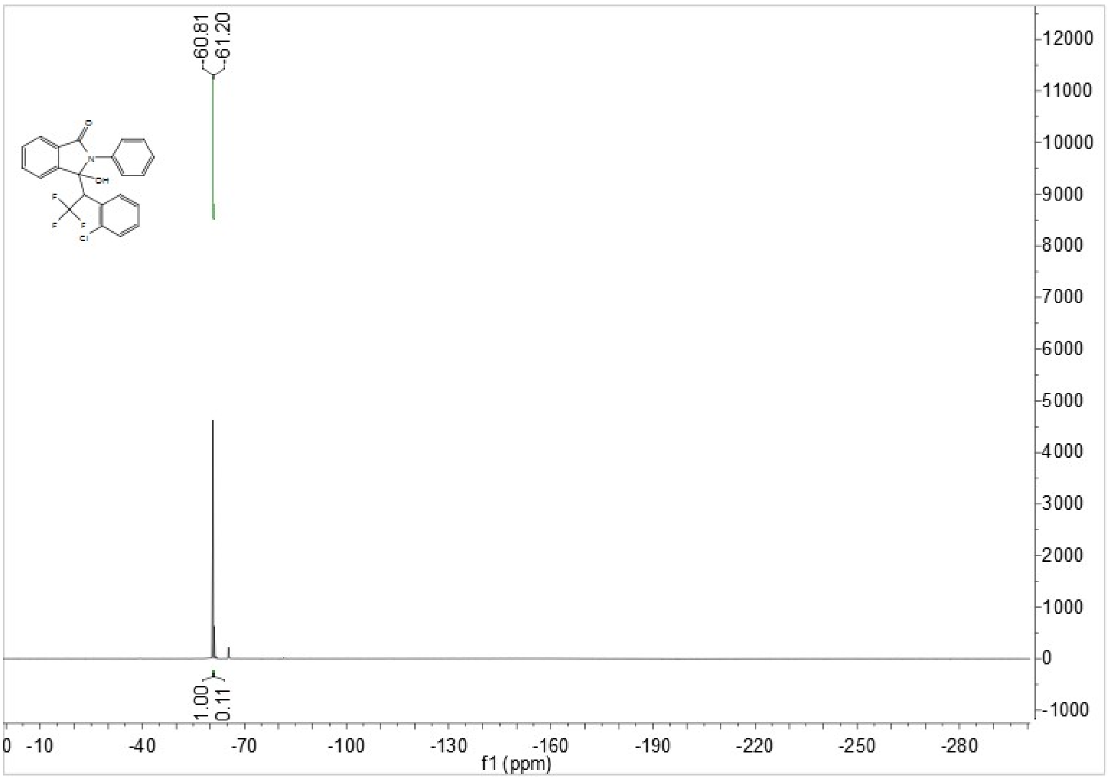
-60.81
-61.20
1.00
0.11
f1 (ppm)

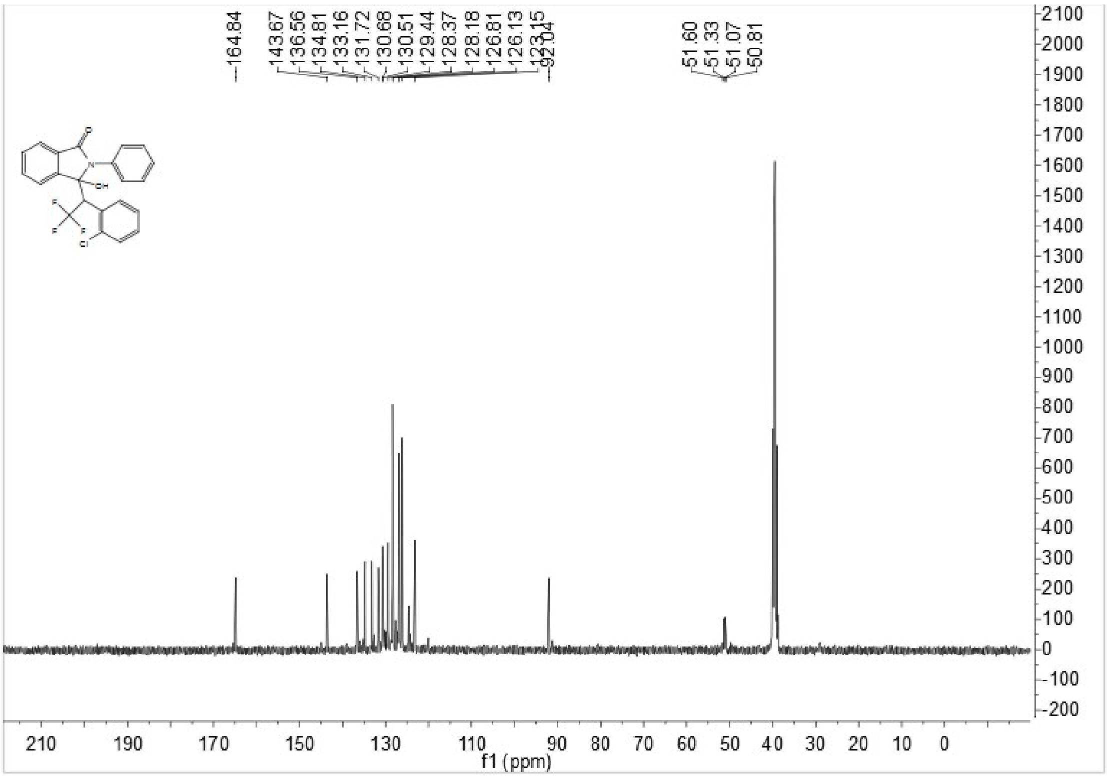

3l

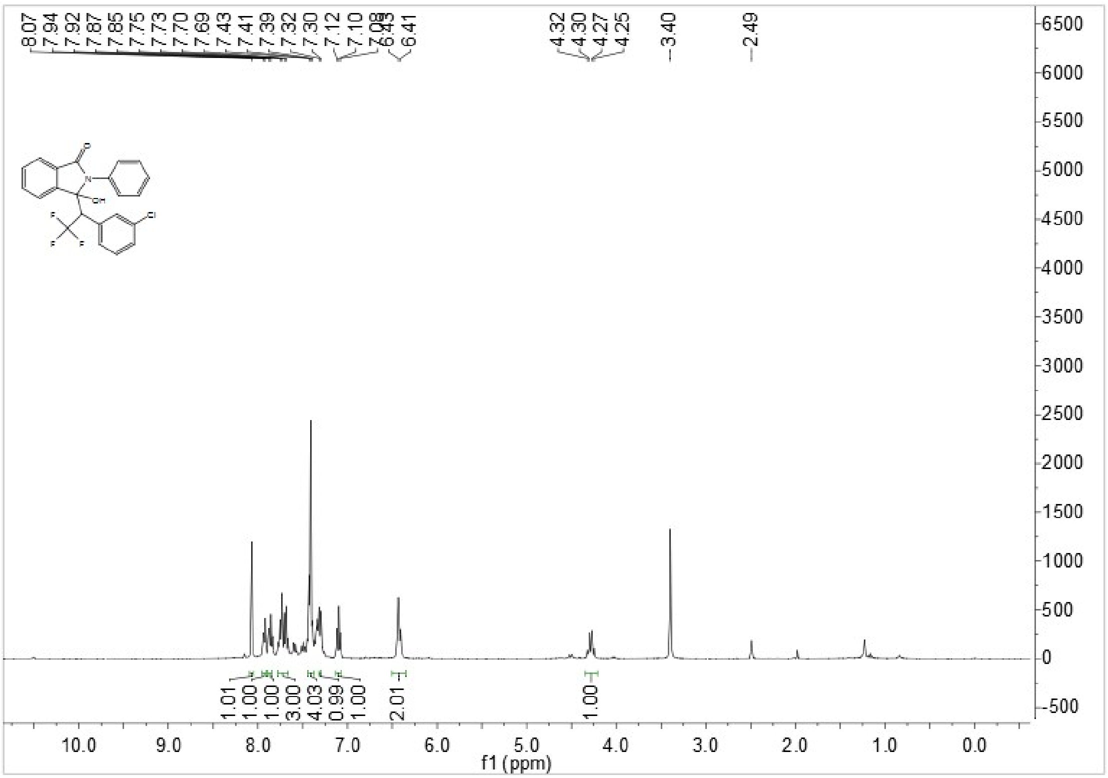

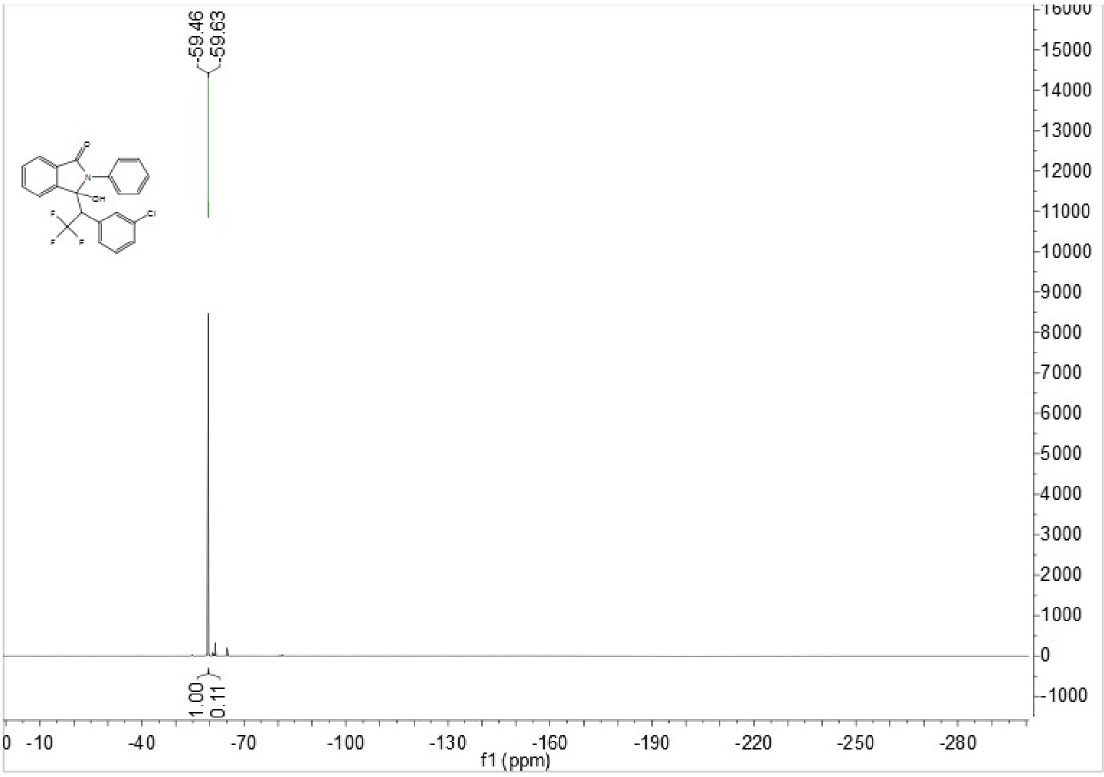
-59.46
-59.63
1.00
0.11
f1 (ppm)

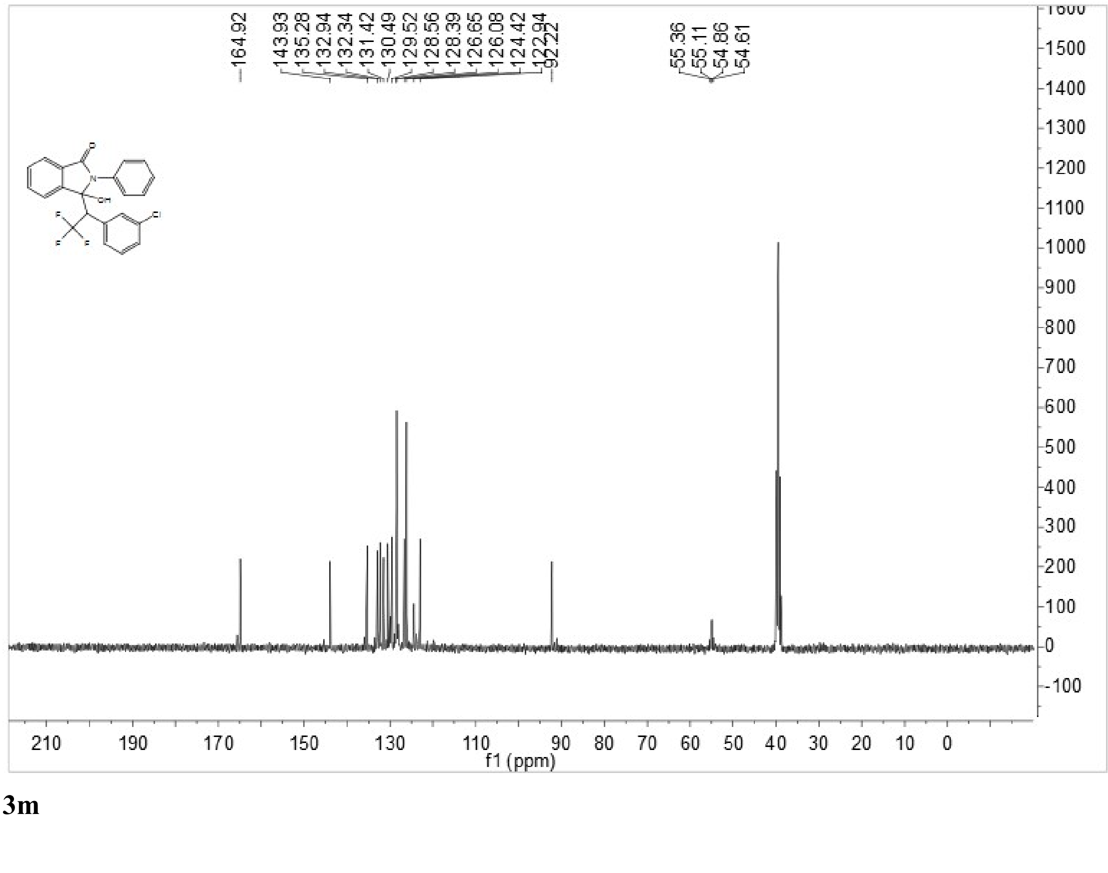
164.92
143.93
135.28
132.94
132.34
131.42
130.49
129.52
128.56
128.39
126.65
126.08
124.42
122.94
92.22
55.36
55.11
54.86
54.61
f1 (ppm)

3m

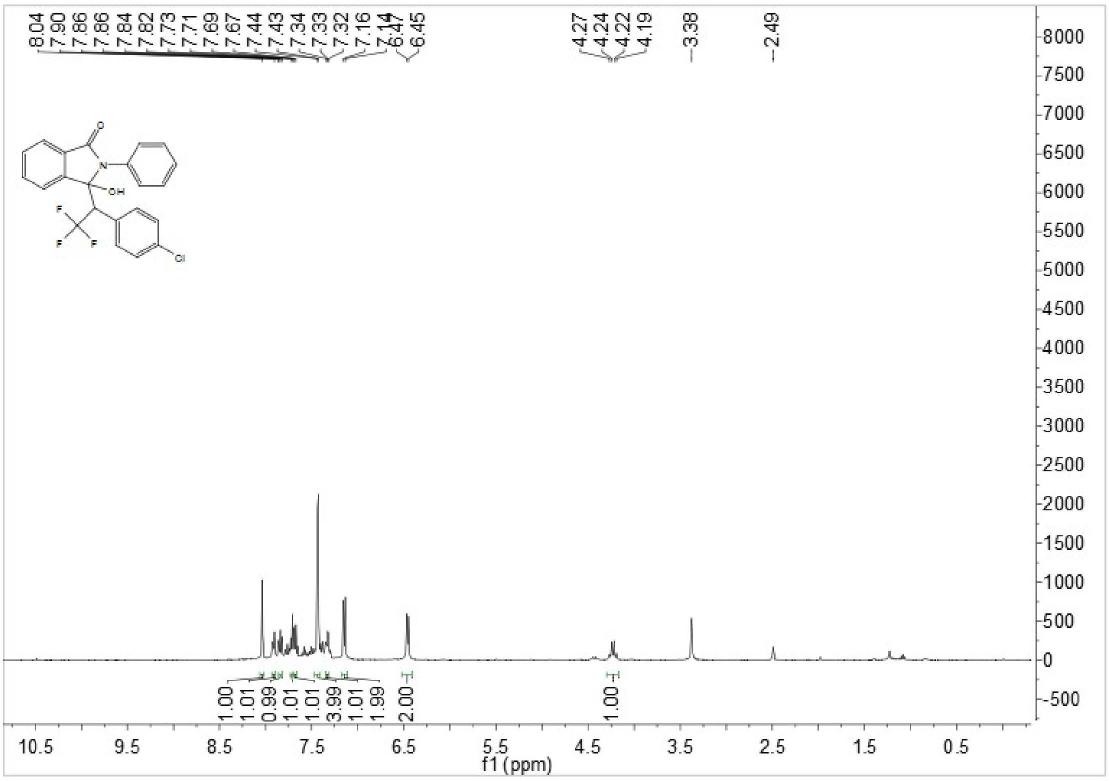

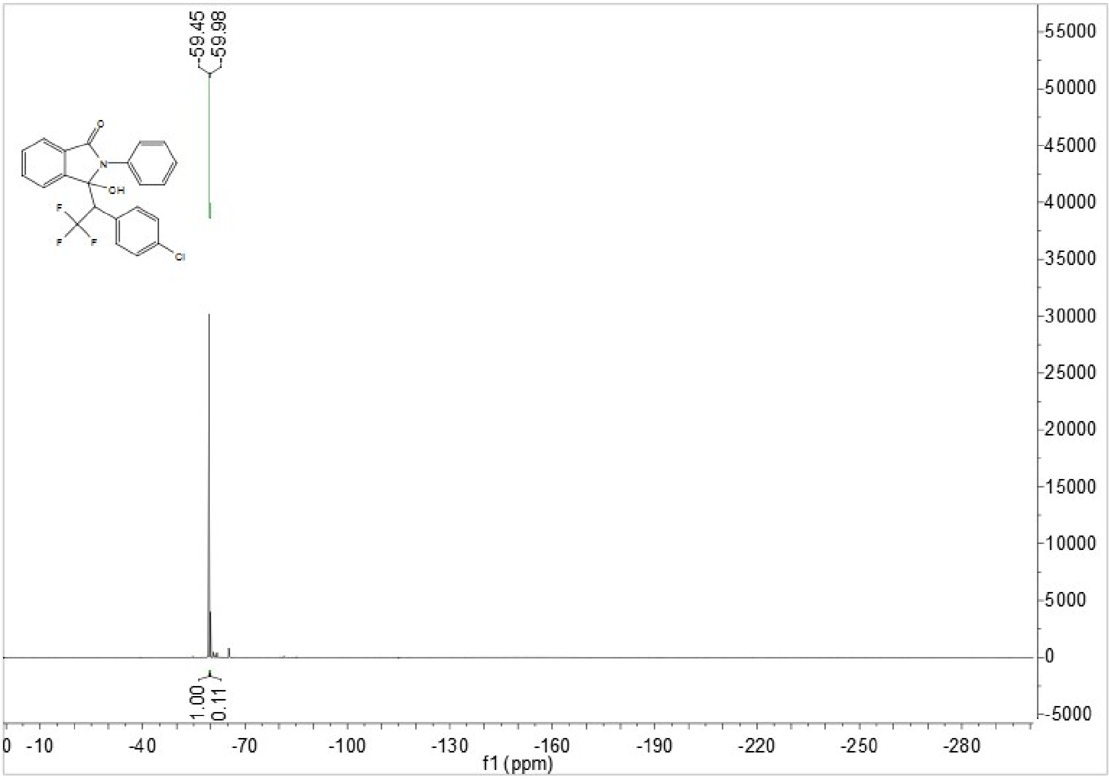

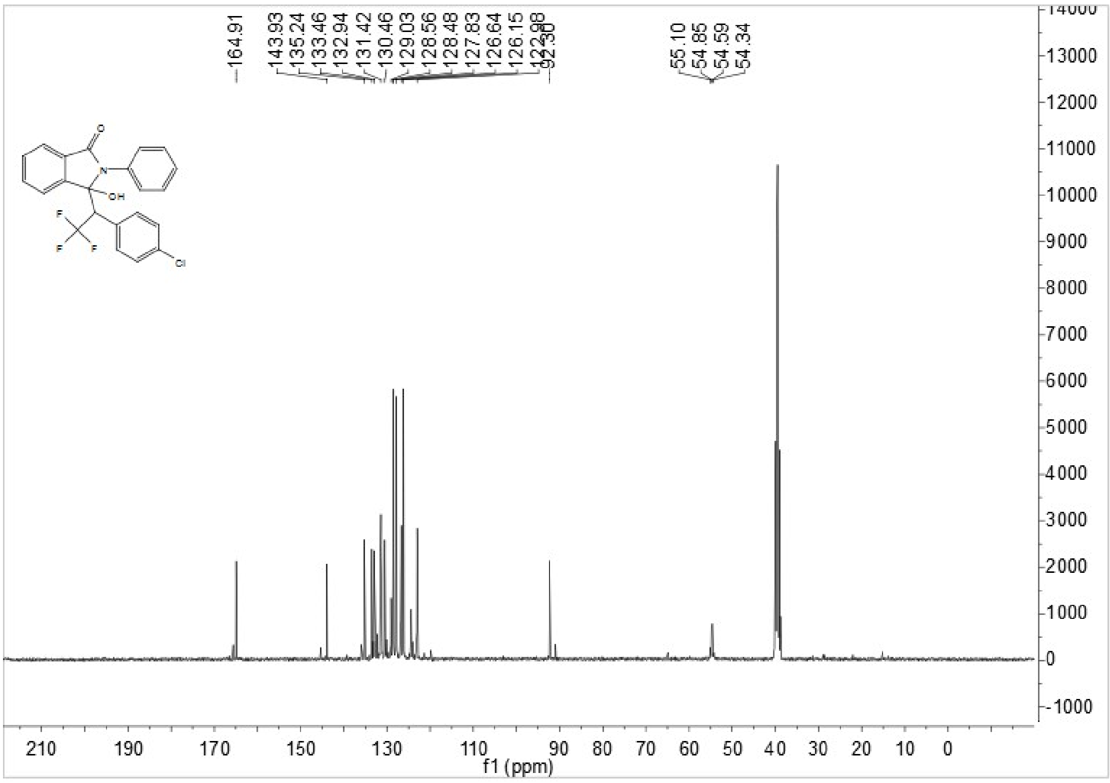
164.91
143.93
135.24
133.46
132.94
131.42
130.46
129.03
128.56
128.48
127.83
126.64
126.15
122.98
92.30
55.10
54.85
54.59
54.34
f1 (ppm)

3n

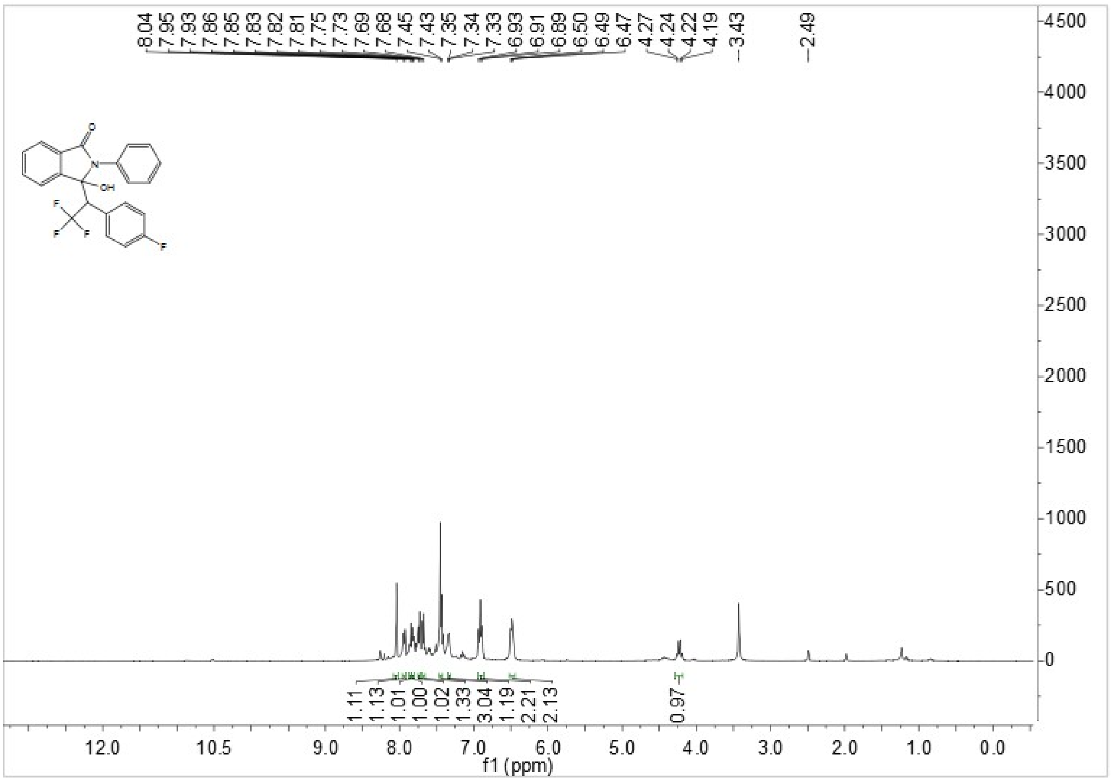
8.04
7.95
7.93
7.86
7.85
7.83
7.82
7.81
7.75
7.73
7.69
7.68
7.45
7.43
7.35
7.34
7.33
6.93
6.91
6.89
6.50
6.49
6.47
4.27
4.24
4.22
4.19
3.43
2.49
1.11
1.13
1.01
1.00
1.02
1.33
3.04
1.19
2.21
2.13
0.97
f1 (ppm)

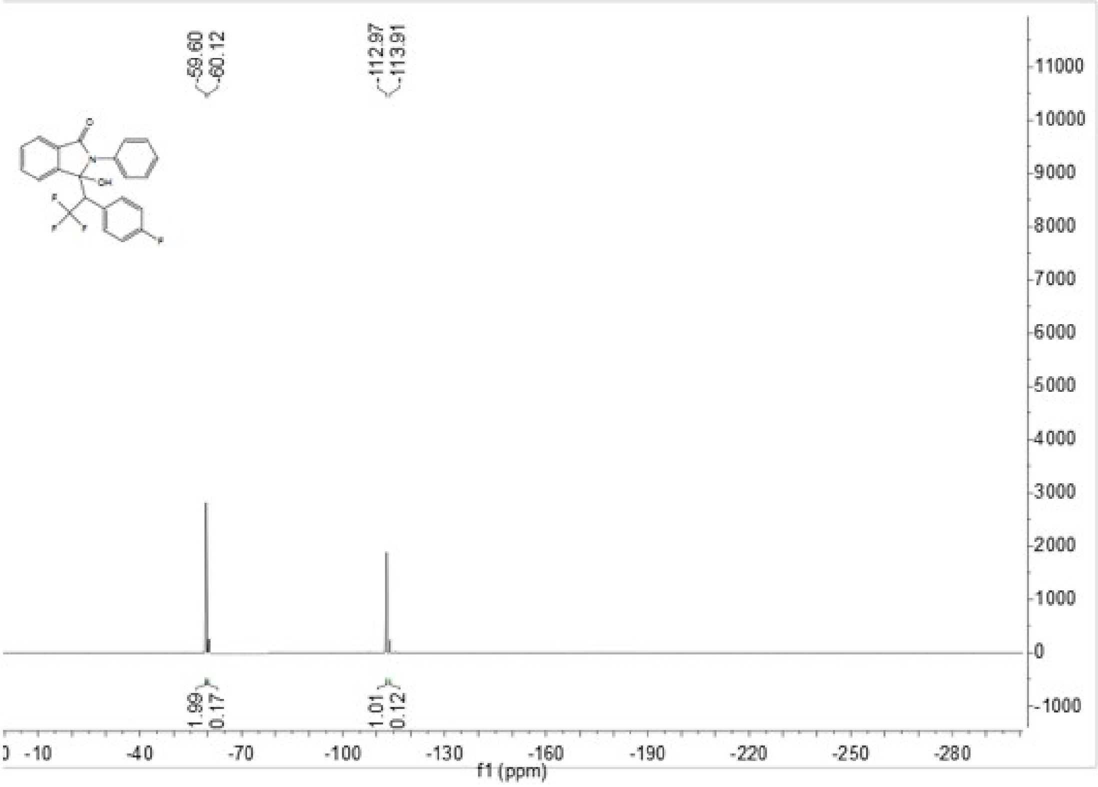
-59.60
-60.12
-112.97
-113.91
1.99
0.17
1.01
0.12
f1 (ppm)
OH
F

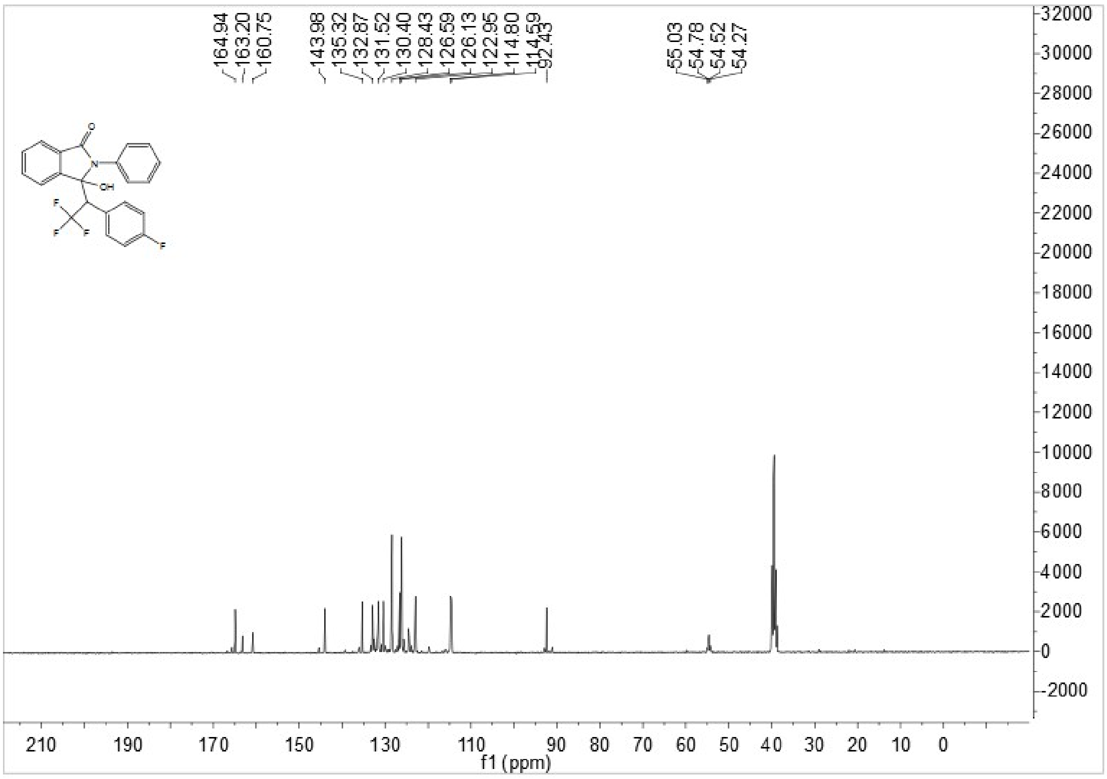
164.94
163.20
160.75
143.98
135.32
132.87
131.52
130.40
128.43
126.59
126.13
122.95
114.80
114.59
92.43
55.03
54.78
54.52
54.27
f1 (ppm)
OH
F

3o

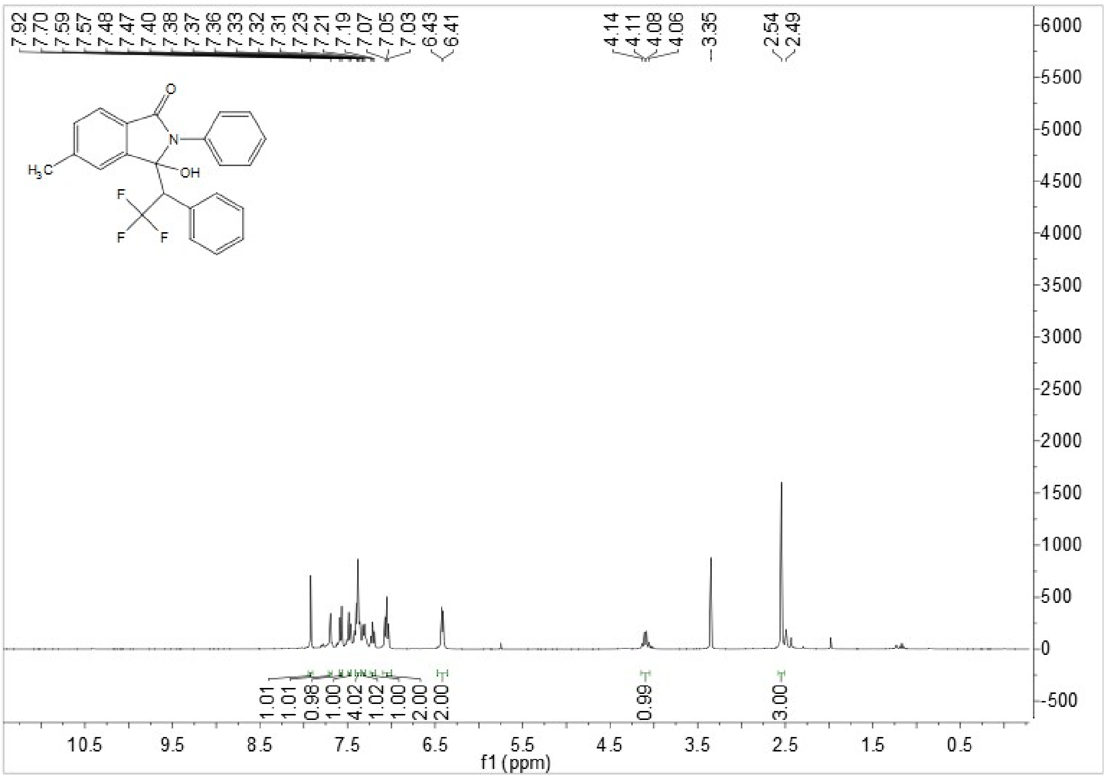
7.92
7.70
7.59
7.57
7.48
7.47
7.40
7.38
7.37
7.36
7.33
7.32
7.31
7.23
7.21
7.19
7.07
7.05
7.03
6.43
6.41
4.14
4.11
4.08
4.06
3.35
2.54
2.49
1.01
1.01
0.98
1.00
4.02
1.02
1.00
2.00
2.00
0.99
3.00
f1 (ppm)

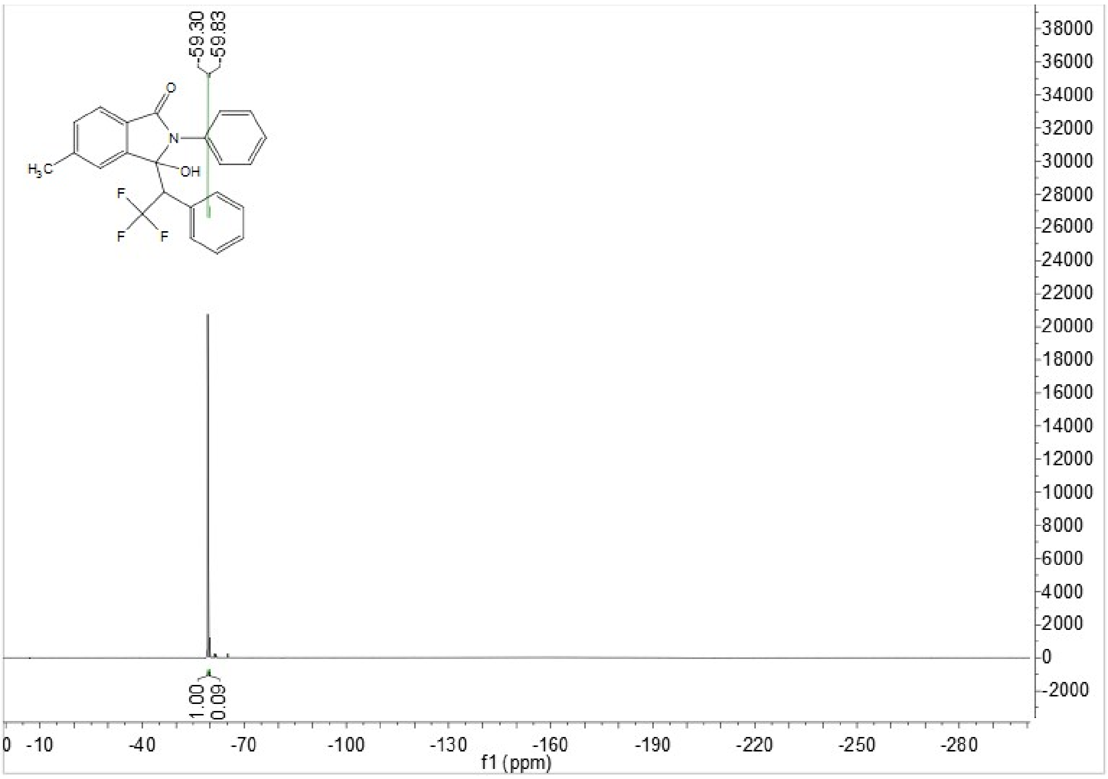
-59.30
-59.83
1.00
0.09
f1 (ppm)

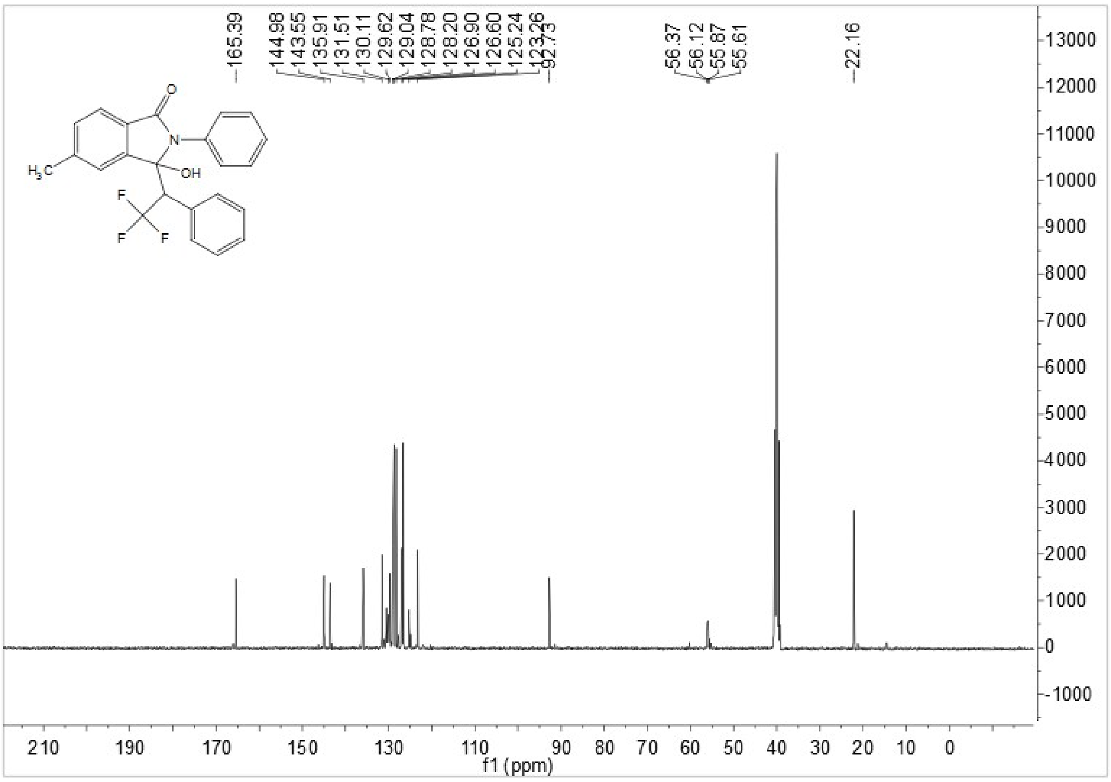

3p

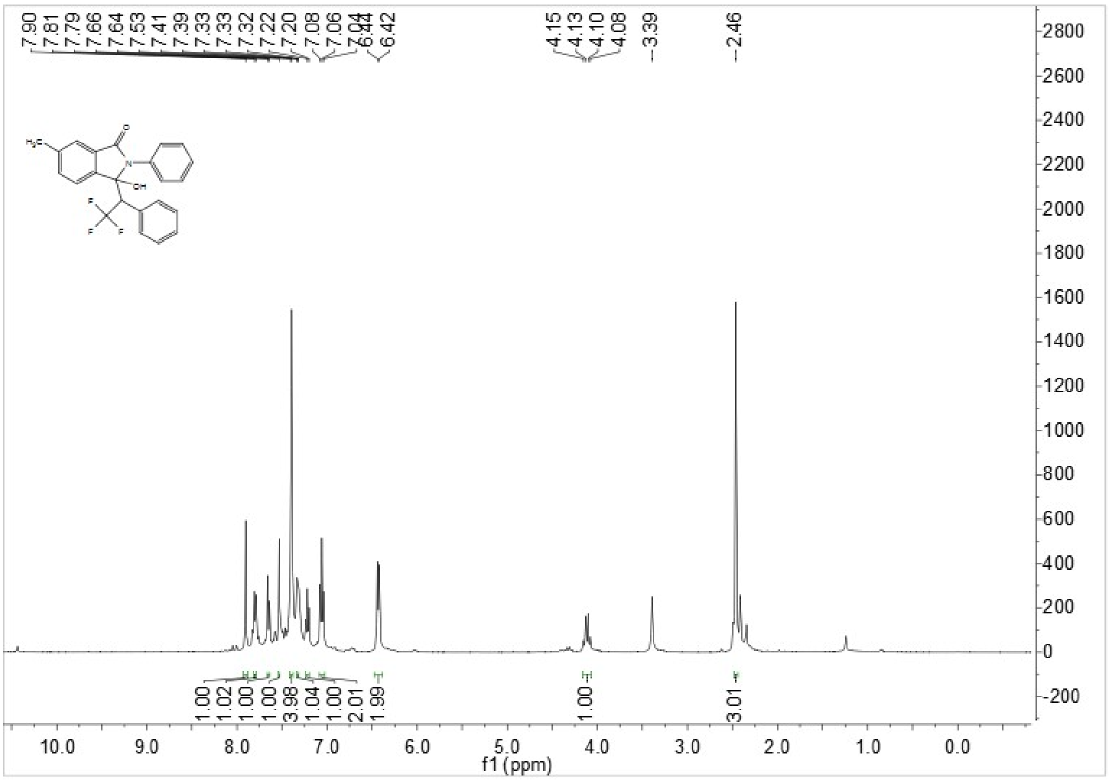

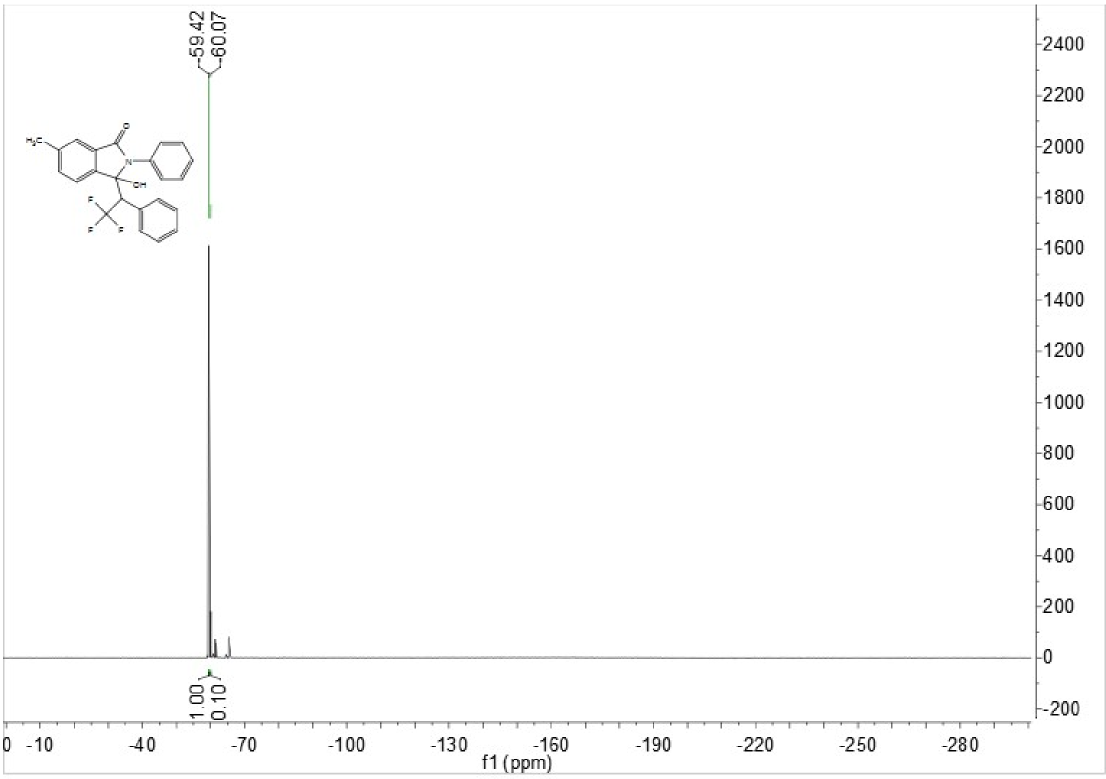

59.42
60.07
1.00
0.10
f1 (ppm)

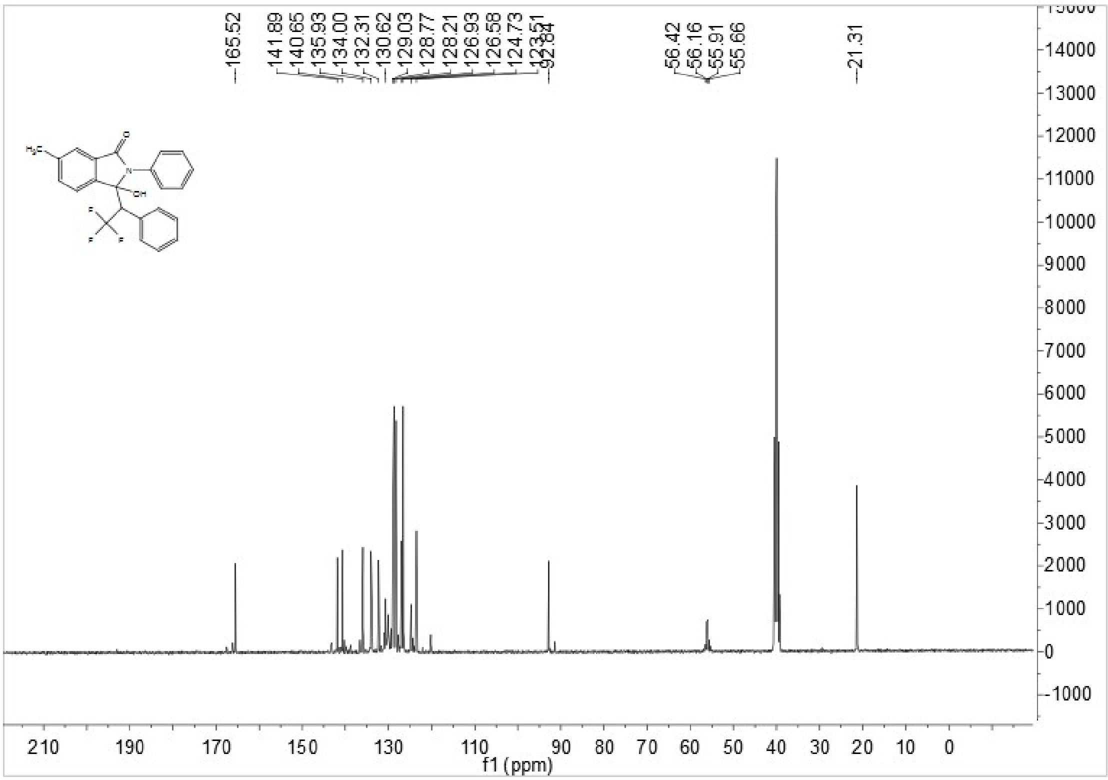

165.52
141.89
140.65
135.93
134.00
132.31
130.62
129.03
128.77
128.21
126.93
126.58
124.73
123.51
92.84
56.42
56.16
55.91
55.66
21.31
f1 (ppm)

3q

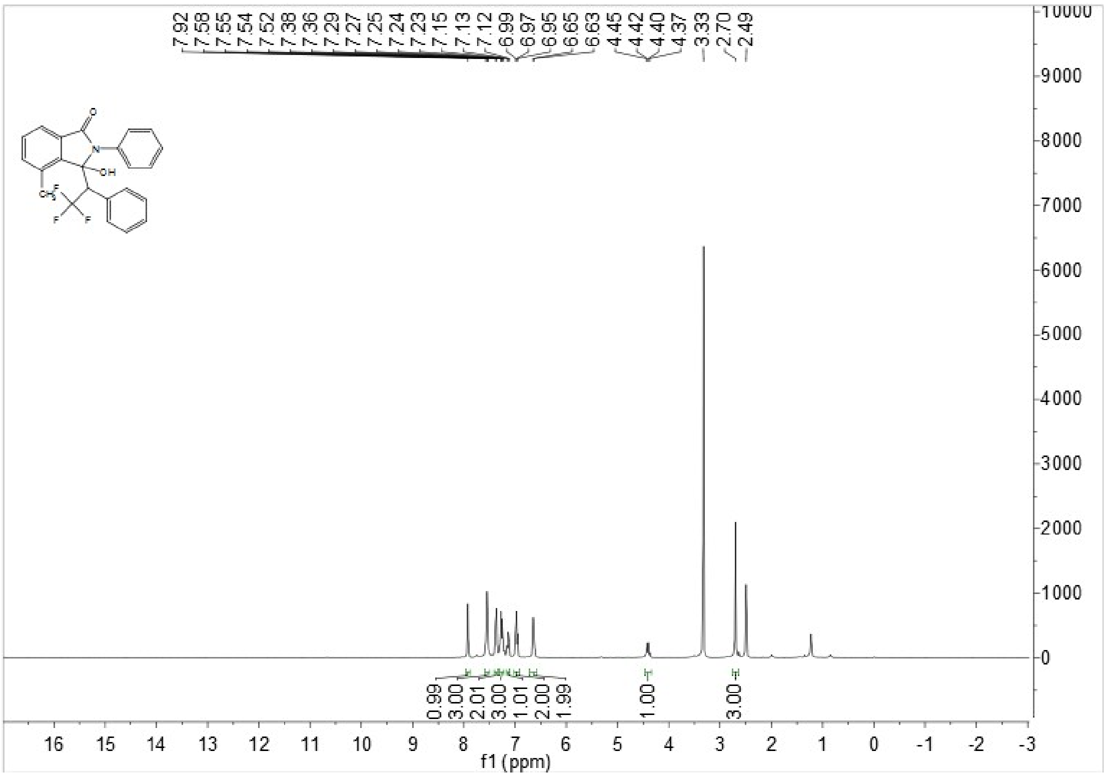
7.92
7.58
7.55
7.54
7.52
7.38
7.36
7.29
7.27
7.25
7.24
7.23
7.15
7.13
7.12
6.99
6.97
6.95
6.65
6.63
4.45
4.42
4.40
4.37
3.33
2.70
2.49
0.99
3.00
2.01
3.00
1.01
2.00
1.99
1.00
3.00
f1 (ppm)

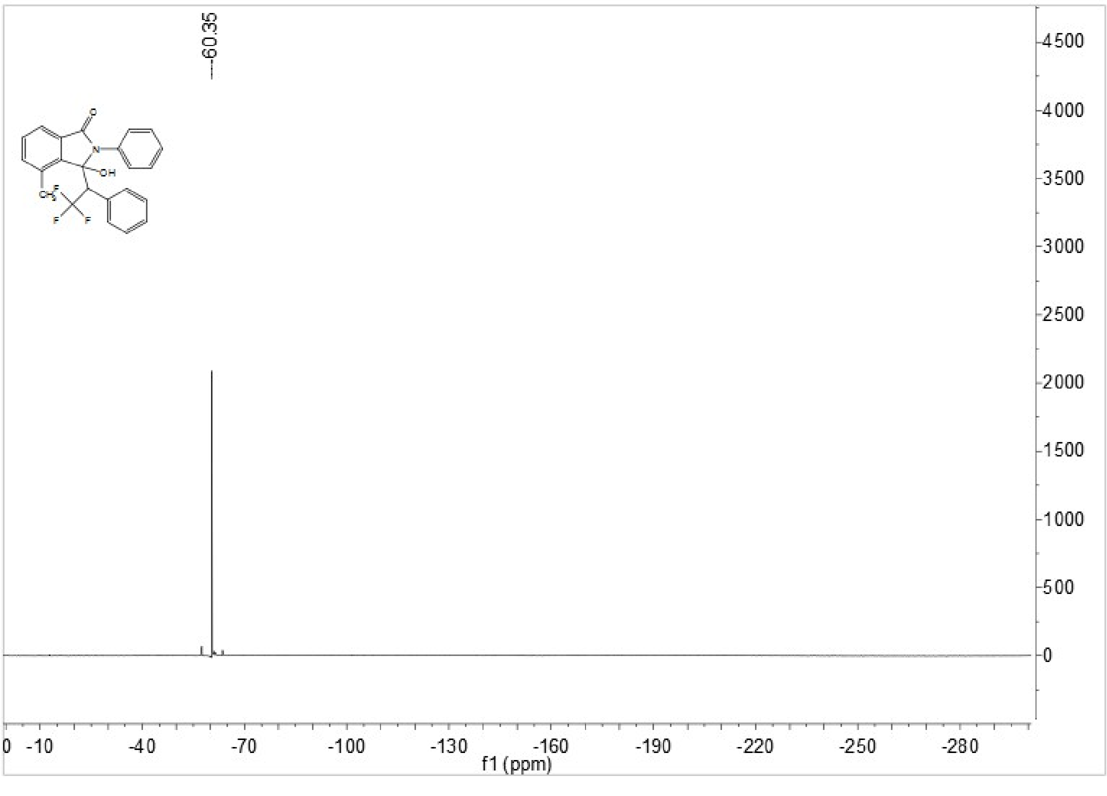
-60.35
f1 (ppm)

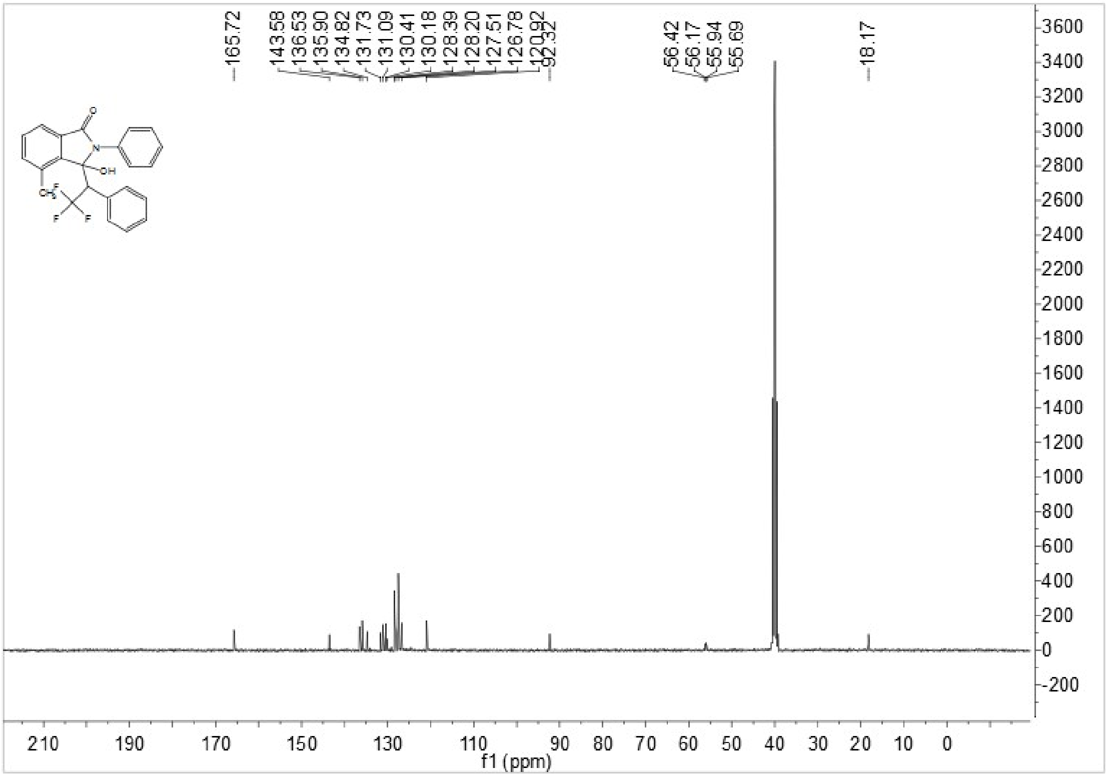

3r

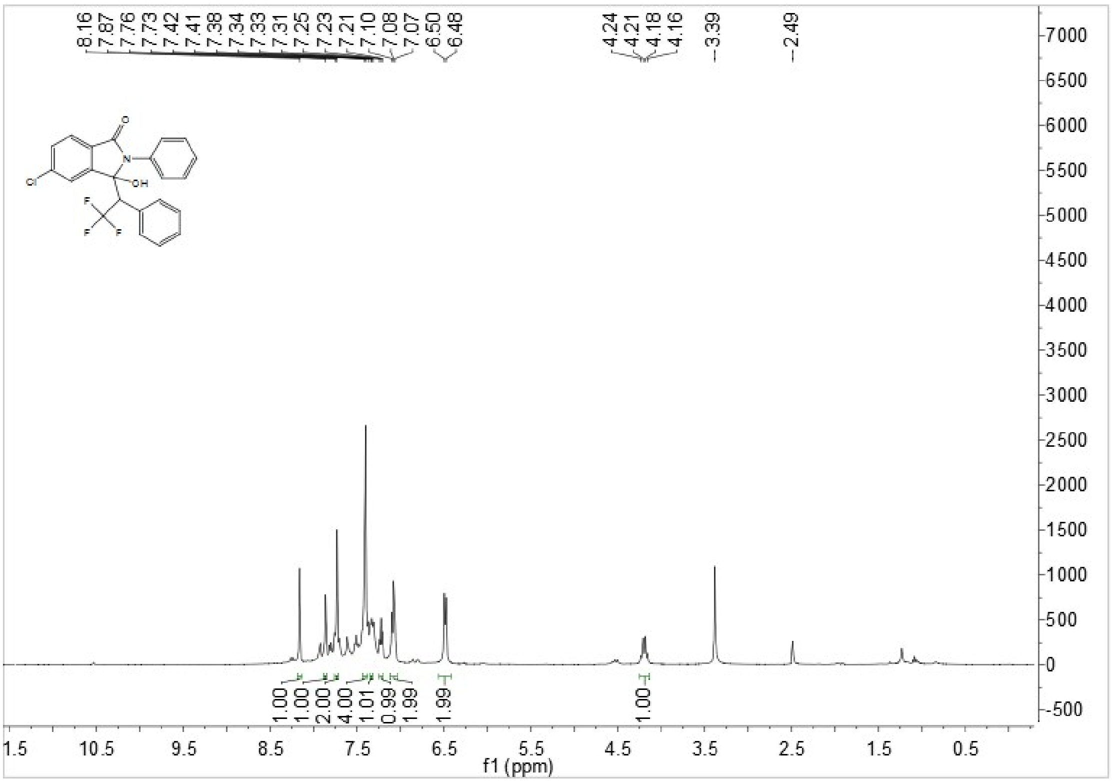

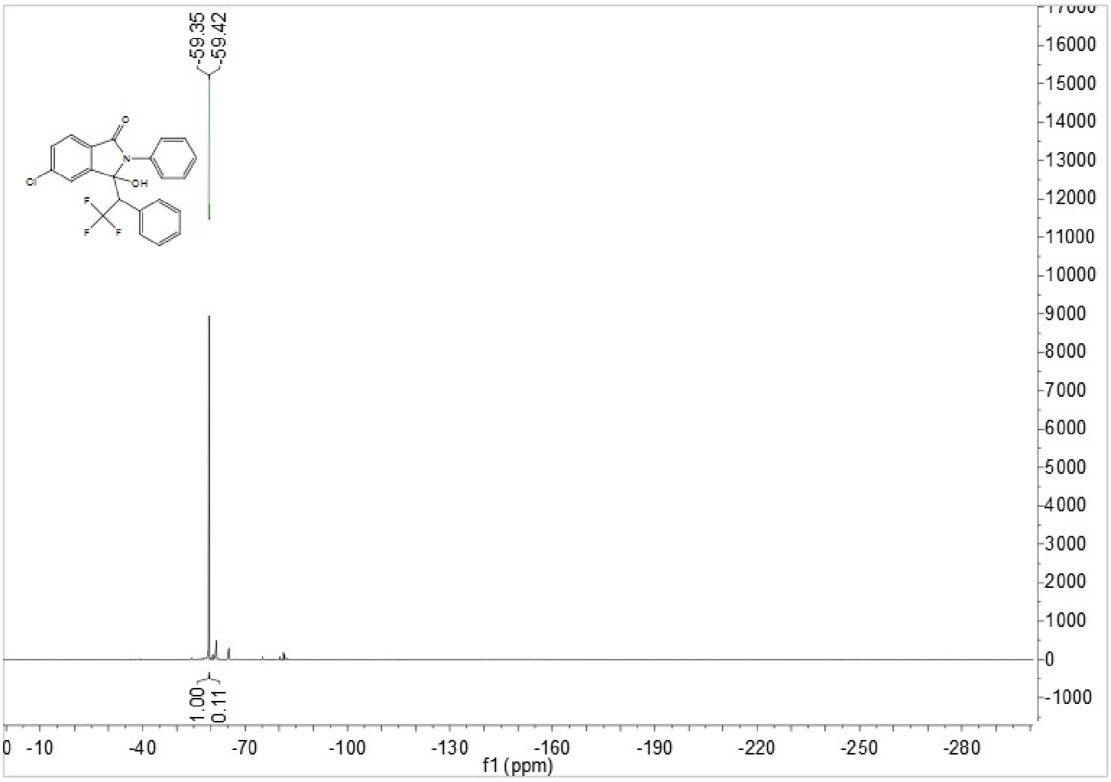
-59.35
-59.42
1.00
0.11
f1 (ppm)

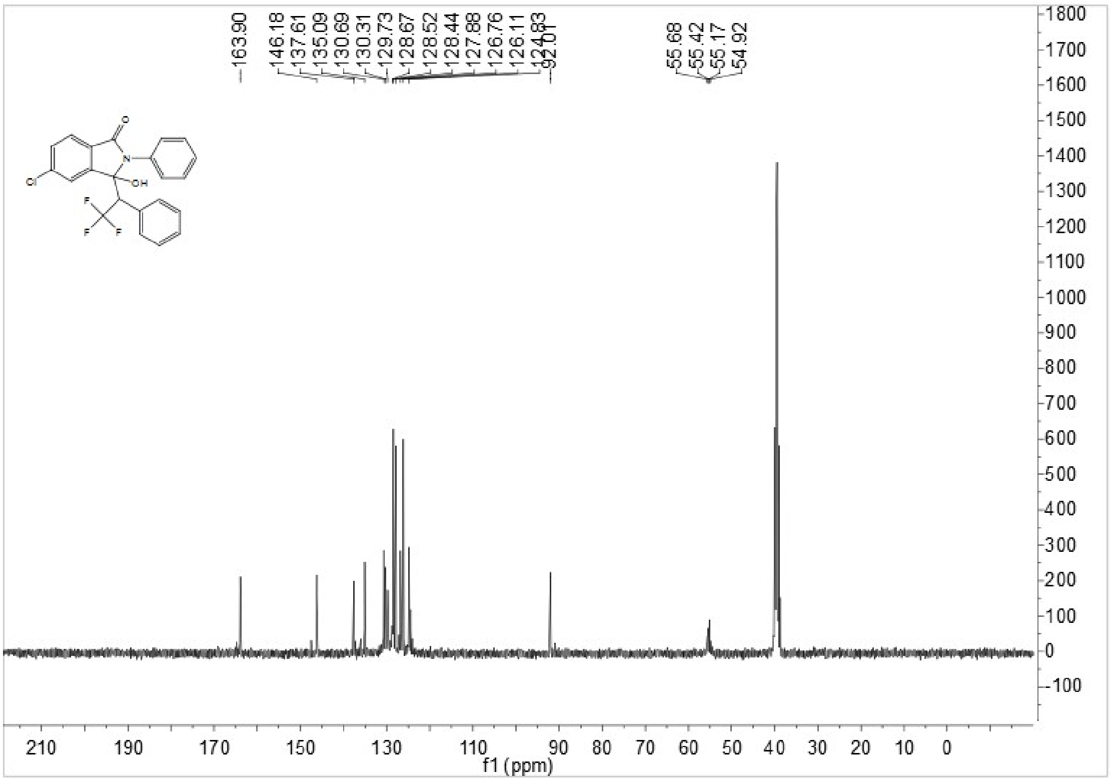
163.90
146.18
137.61
135.09
130.69
130.31
129.73
128.67
128.52
128.44
127.88
126.76
126.11
124.83
92.01
55.68
55.42
55.17
54.92
f1 (ppm)

3s

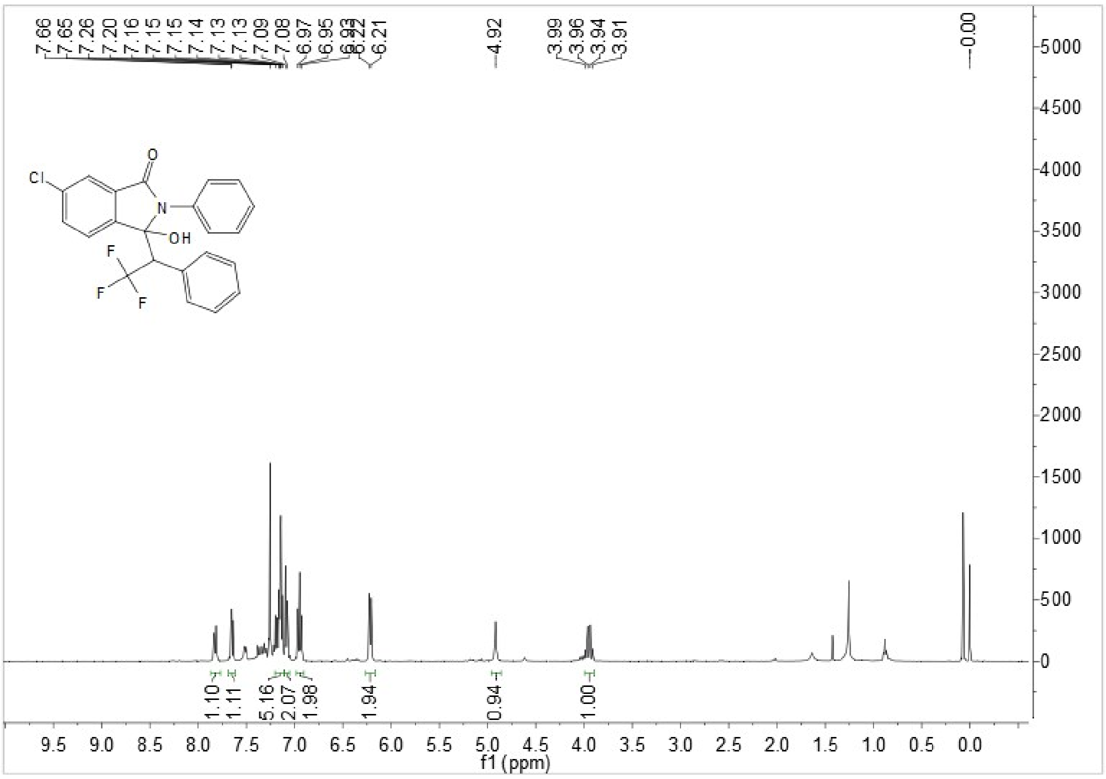
7.66
7.65
7.26
7.20
7.16
7.15
7.15
7.14
7.13
7.13
7.09
7.08
6.97
6.95
6.93
6.22
6.21
4.92
3.99
3.96
3.94
3.91
0.00
Cl
O
N
OH
F
F
F
1.10
1.11
5.16
2.07
1.98
1.94
0.94
1.00
f1 (ppm)

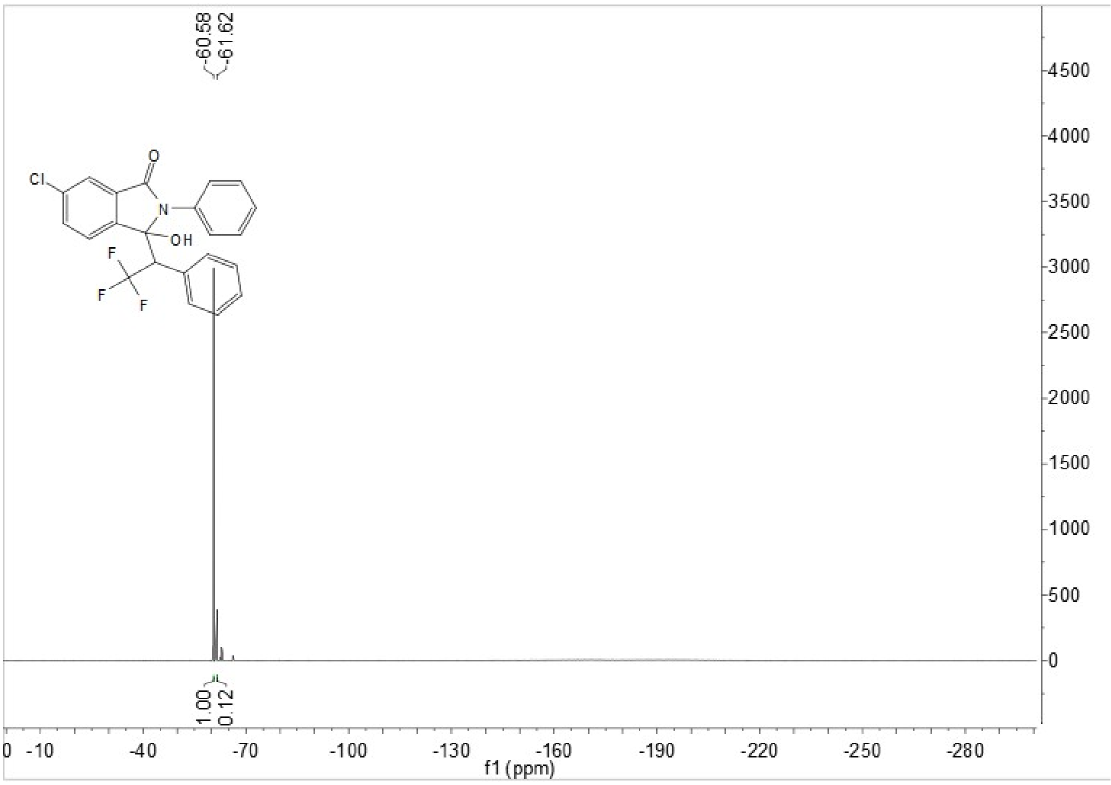
-60.58
-61.62
Cl
O
N
OH
F
F
F
1.00
0.12
f1 (ppm)

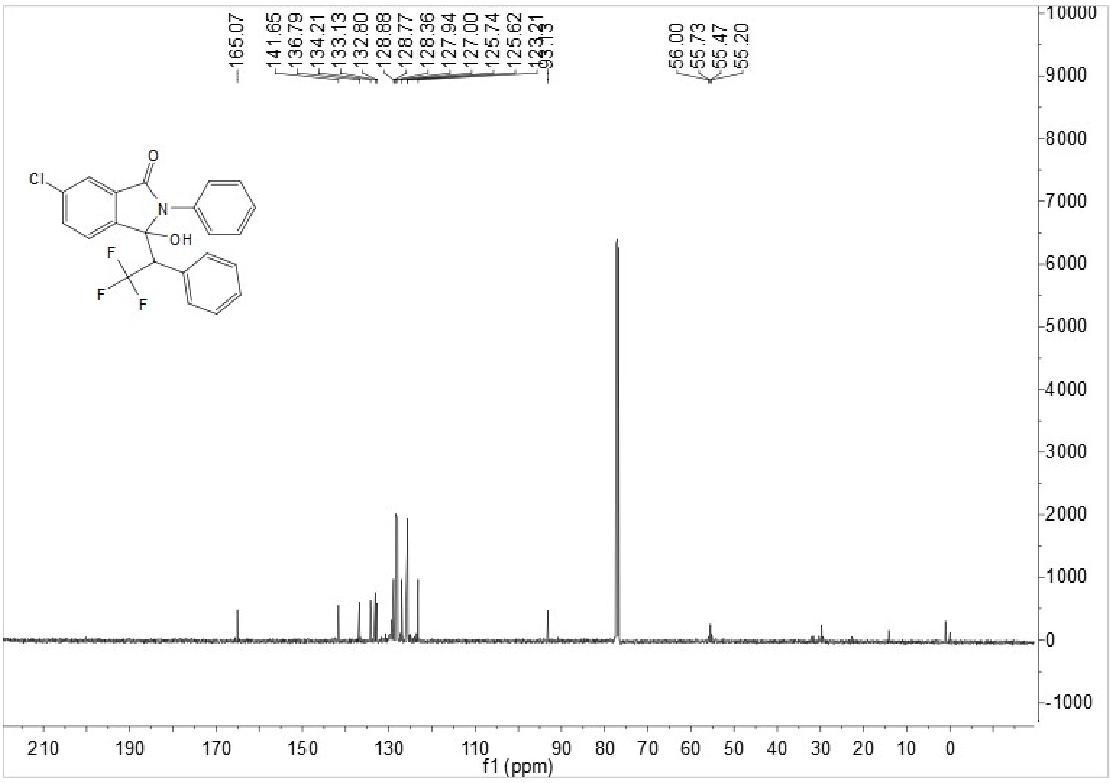
165.07
141.65
136.79
134.21
133.13
132.80
128.88
128.77
128.36
127.94
127.00
125.74
125.62
123.21
93.13
56.00
55.73
55.47
55.20
f1 (ppm)

3t

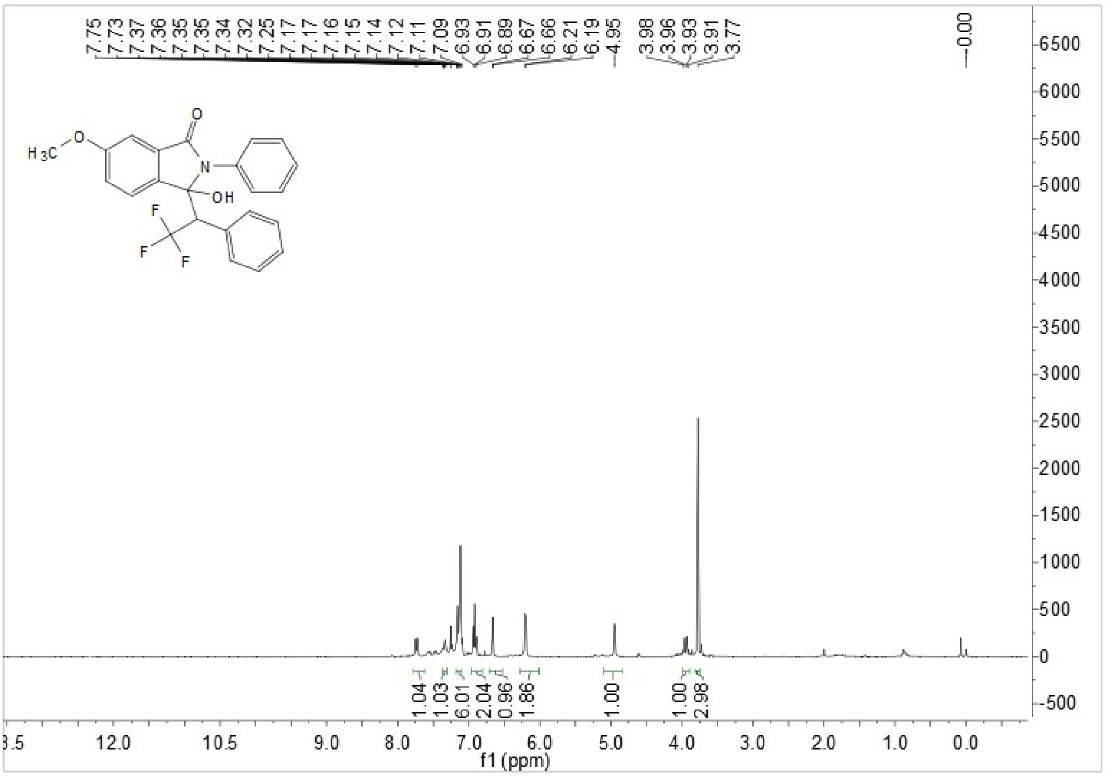
7.75
7.73
7.37
7.36
7.35
7.35
7.34
7.32
7.25
7.17
7.17
7.16
7.15
7.14
7.12
7.11
7.09
6.93
6.91
6.89
6.67
6.66
6.21
6.19
4.95
3.98
3.96
3.93
3.91
3.77
0.00
1.04
1.03
6.01
2.04
0.96
1.86
1.00
1.00
2.98
f1 (ppm)

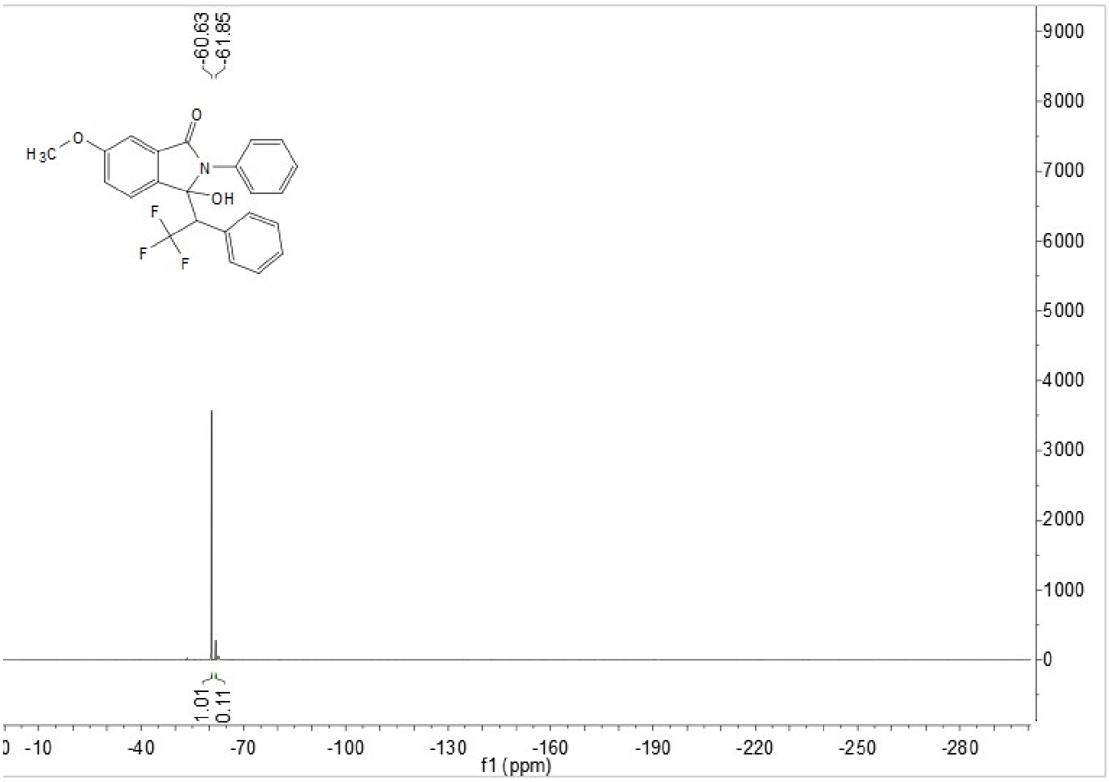
-60.63
-61.85
H3C O
O
N
OH
F
F
F
1.01
0.11
-10 -40 -70 -100 -130 -160 -190 -220 -250 -280
f1 (ppm)
9000 8000 7000 6000 5000 4000 3000 2000 1000 0

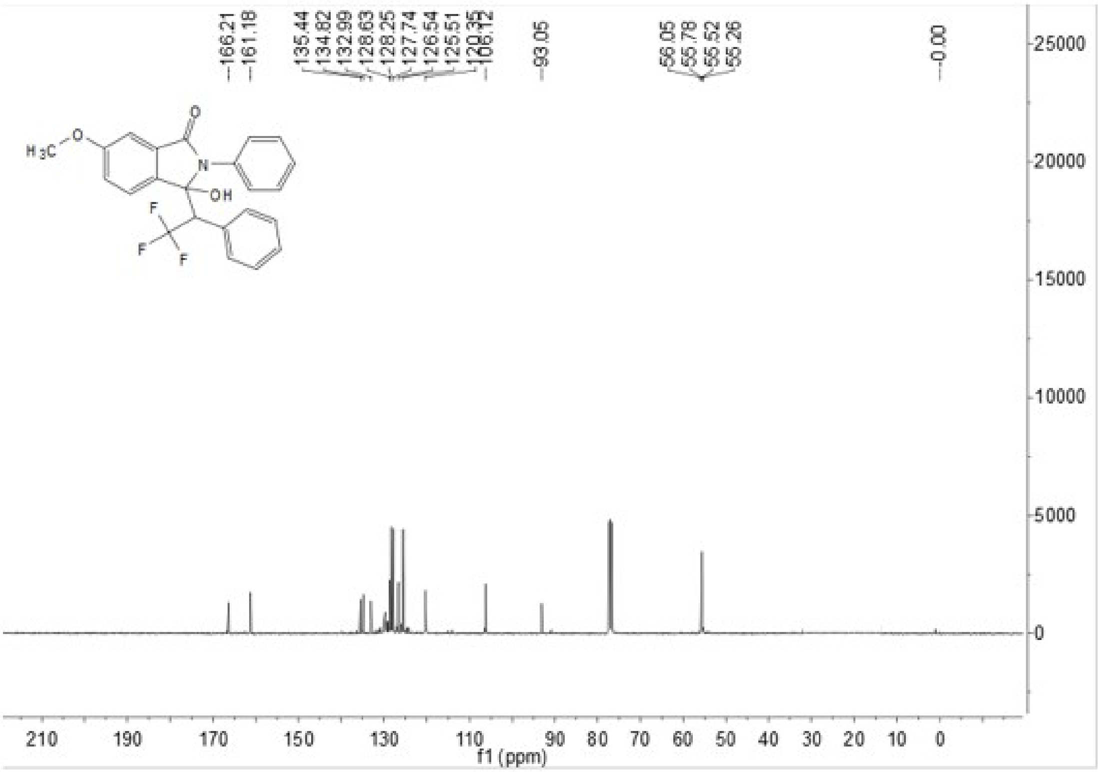
166.21
161.18
135.44
134.82
132.99
128.63
128.25
127.74
126.54
125.51
93.05
56.05
55.78
55.52
55.26
0.00
H3C O
O
N
OH
F
F
F
210 190 170 150 130 110 90 80 70 60 50 40 30 20 10 0
f1 (ppm)
25000 20000 15000 10000 5000 0

3u

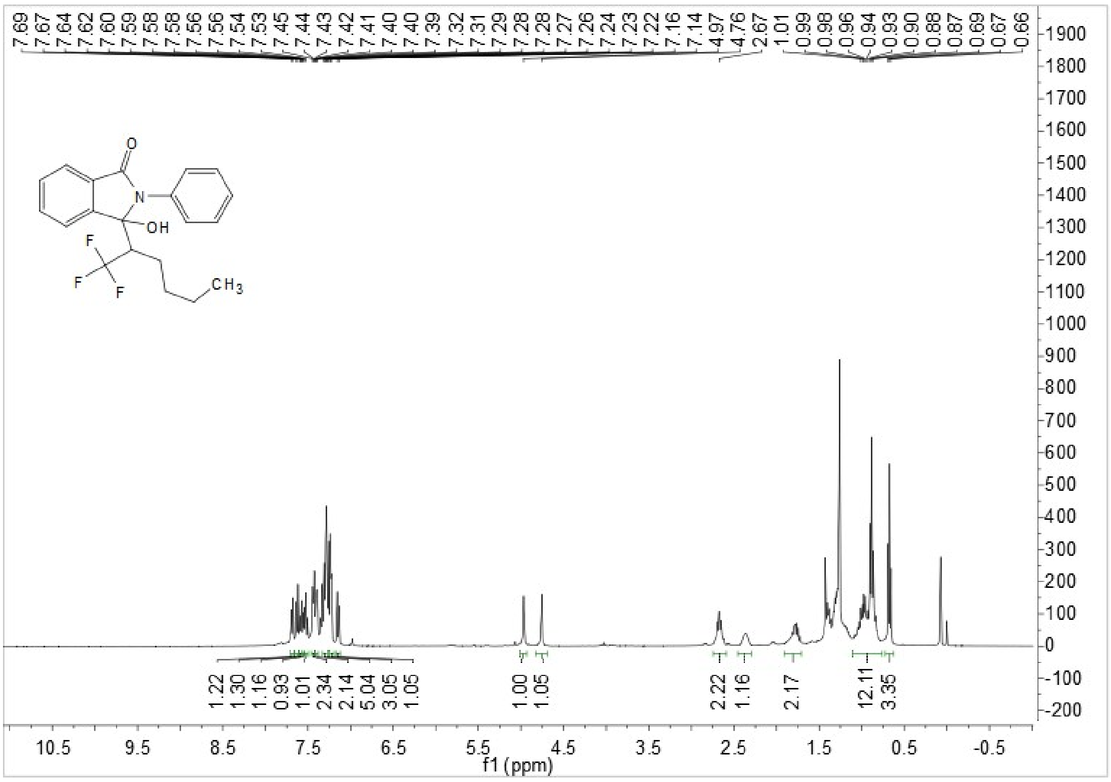

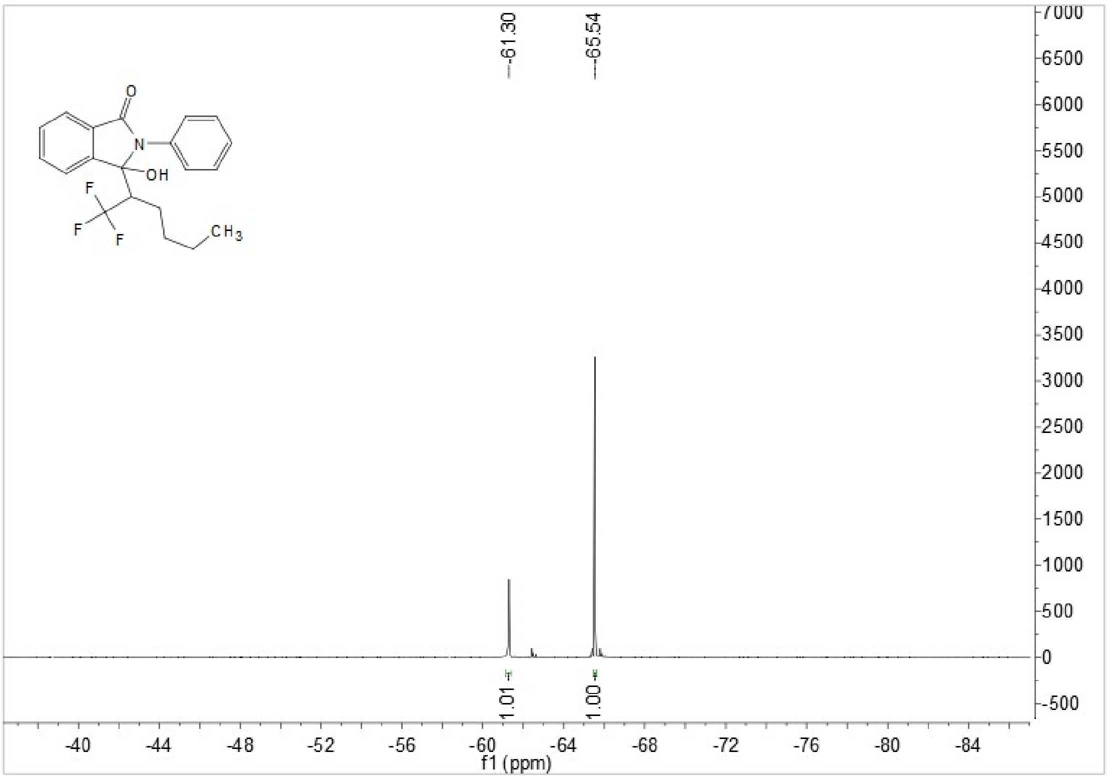

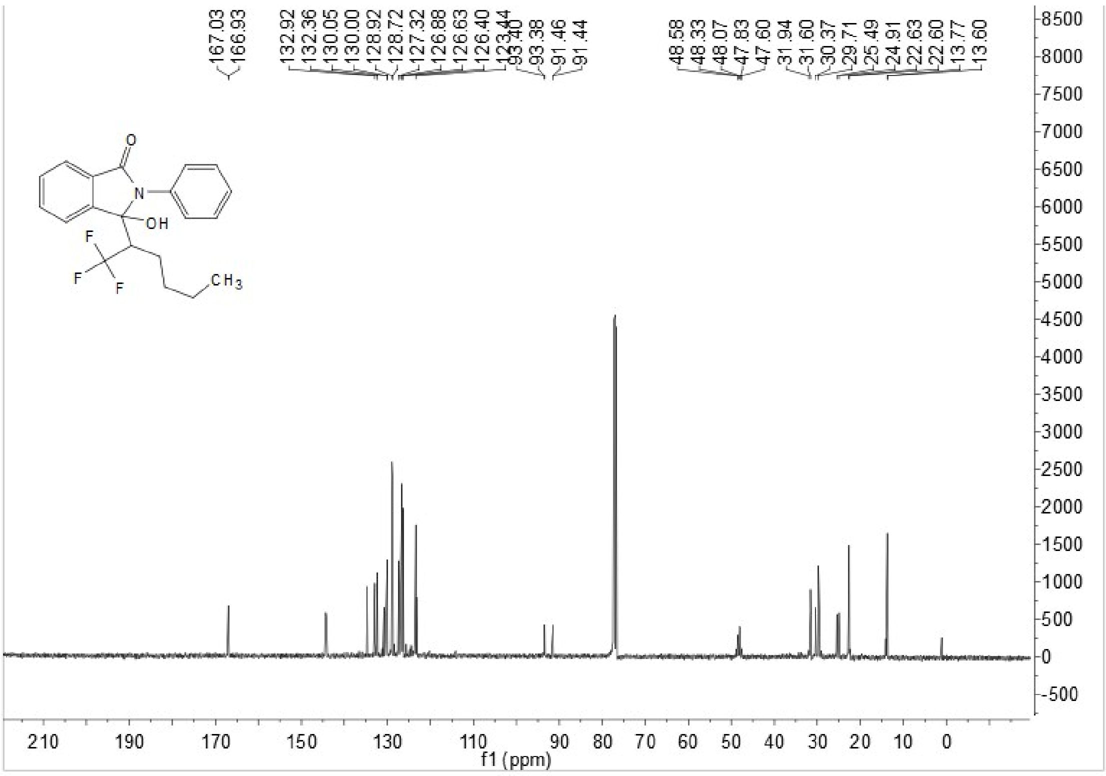

(3,3,3-trifluoroprop-1-ene-1,1-diyl)dibenzene

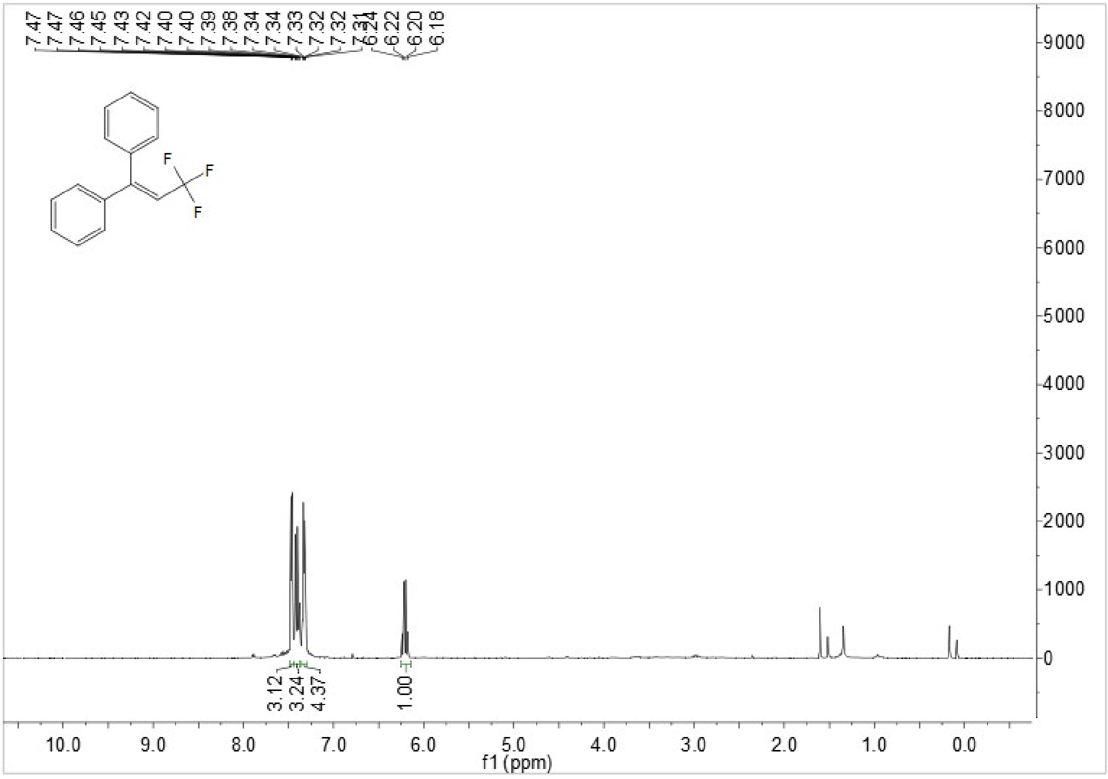

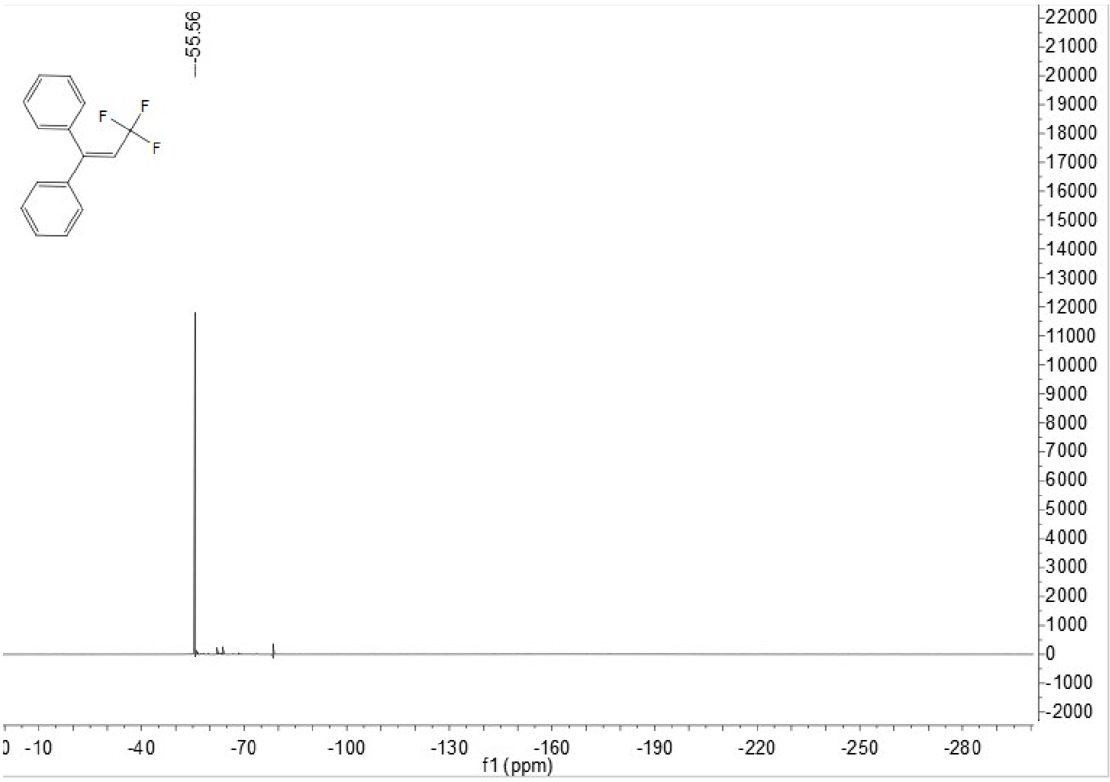

-55.56
F
F
F
f1 (ppm)

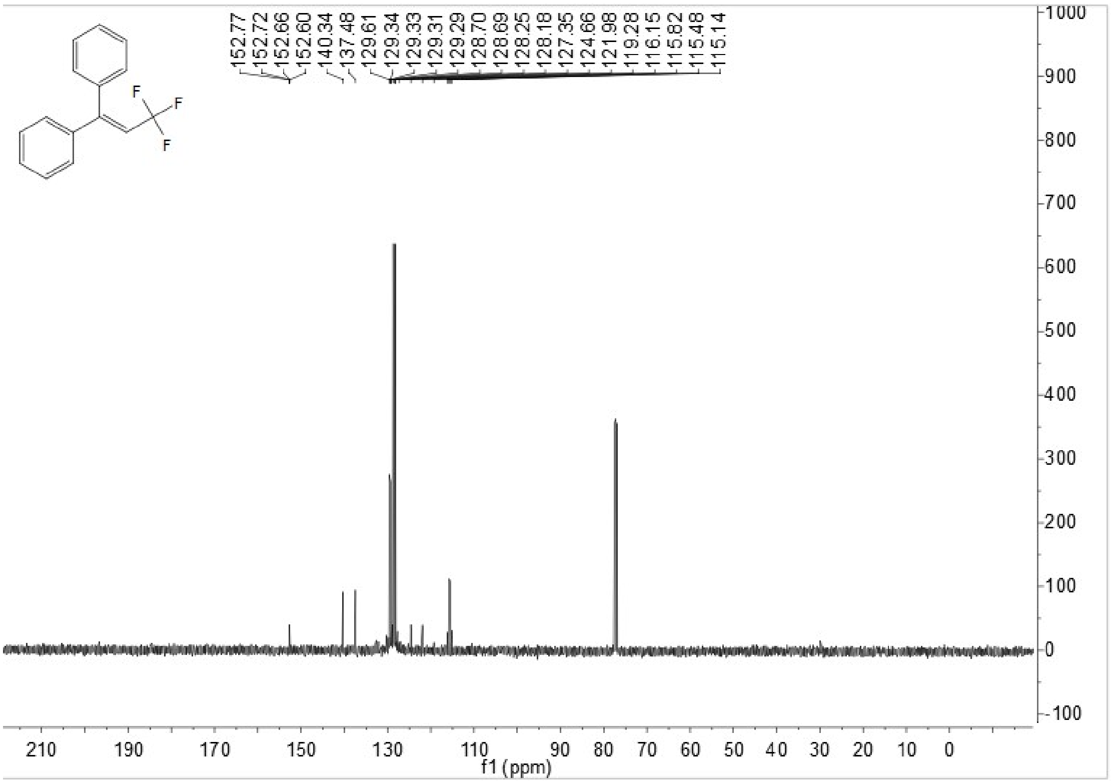

152.77
152.72
152.66
152.60
140.34
137.48
129.61
129.34
129.33
129.31
129.29
128.70
128.69
128.25
128.18
127.35
124.66
121.98
119.28
116.15
115.82
115.48
115.14
F
F
F
f1 (ppm)

4a-HRMS

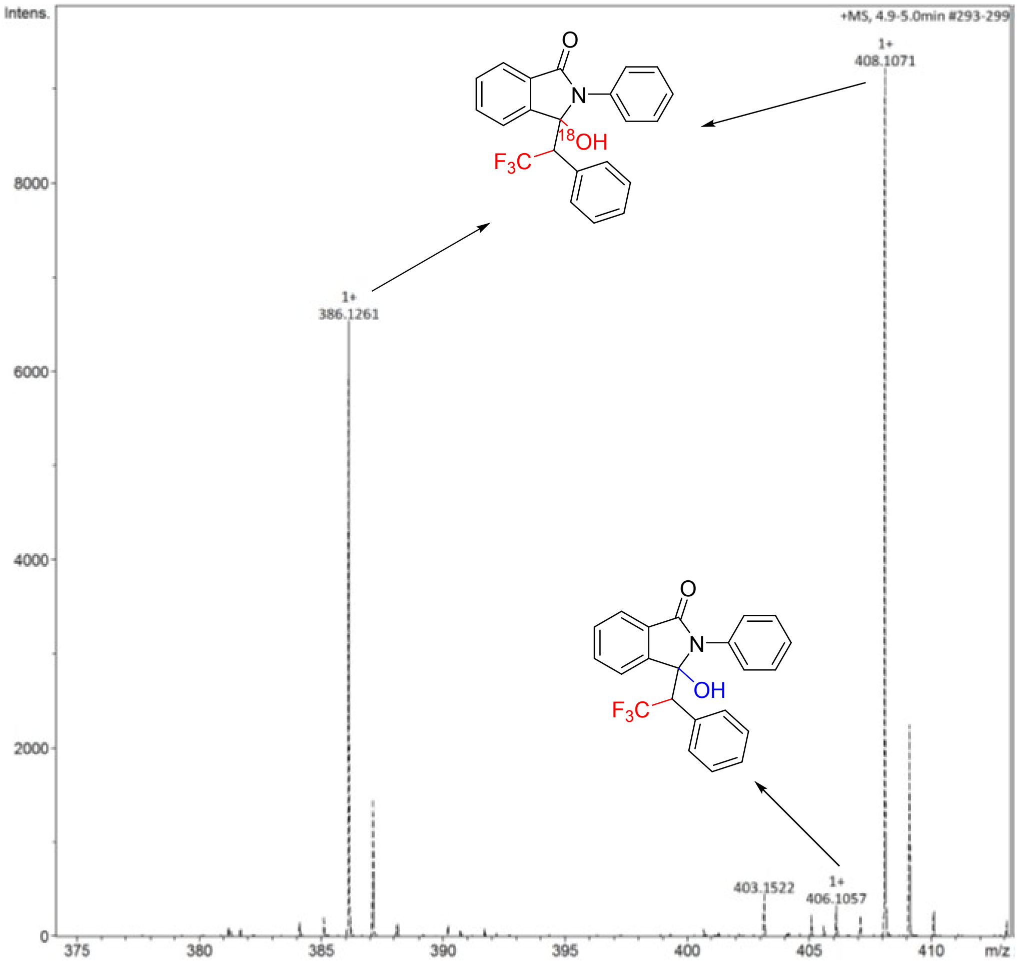
Intens.
+MS, 4.9-5.0min #293-299
1+
408.1071
1+
386.1261
403.1522
1+
406.1057
8000
6000
4000
2000
0
375
380
385
390
395
400
405
410
m/z
O
N
18OH
F3C
OH
